# Preclinical safety and hepatotoxicity evaluation of biomineralized copper sulfide nanoagents

**DOI:** 10.1186/s12951-022-01399-5

**Published:** 2022-04-12

**Authors:** Ya-Nan Xia, He Zu, Haoxiang Guo, Tianyan Jiang, Siqi Yang, Huan Yu, Shaodian Zhang, Heng Ding, Xiaoyu Li, Yangyun Wang, Yong Wang, Leshuai W. Zhang

**Affiliations:** 1 State Key Laboratory of Radiation Medicine and Protection, School of Radiation Medicine and Protection, School for Radiological and Interdisciplinary Sciences (RAD-X), Collaborative Innovation Center of Radiation Medicine of Jiangsu Higher Education Institutions, Soochow University, 199 Renai Rd, Suzhou, 215123 Jiangsu Province People’s Republic of China; 2 Department of Otolaryngology, The First Affiliated Hospital of Soochow University, Suzhou, 215123 China; 3 The Second Affiliated Hospital of Soochow University, Suzhou, 215123 China; 4 GeneScience Pharmceuticals Co., Ltd, Changchun, 130012 China

**Keywords:** Cu_2−x_S nanoagents, Biomineralization, Safety evaluation, Hepatotoxicity, Metabolism pathway

## Abstract

Albumin-biomineralized copper sulfide nanoparticles (Cu_2−x_S NPs) have attracted much attention as an emerging phototheranostic agent due to their advantages of facile preparation method and high biocompatibility. However, comprehensive preclinical safety evaluation is the only way to meet its further clinical translation. We herein evaluate detailedly the safety and hepatotoxicity of bovine serum albumin-biomineralized Cu_2−x_S (BSA@Cu_2−x_S) NPs with two different sizes in rats. Large-sized (LNPs, 17.8 nm) and small-sized (SNPs, 2.8 nm) BSA@Cu_2−x_S NPs with great near-infrared absorption and photothermal conversion efficiency are firstly obtained. Seven days after a single-dose intravenous administration, SNPs distributed throughout the body are cleared primarily through the feces, while a large amount of LNPs remained in the liver. A 14-day subacute toxicity study with a 28-day recovery period are conducted, showing long-term hepatotoxicity without recovery for LNPs but reversible toxicity for SNPs. Cellular uptake studies indicate that LNPs prefer to reside in Kupffer cells, leading to prolonged and delayed hepatotoxicity even after the cessation of NPs administration, while SNPs have much less Kupffer cell uptake. RNA-sequencing analysis for gene expression indicates that the inflammatory pathway, lipid metabolism pathway, drug metabolism-cytochrome P450 pathway, cholesterol/bile acid metabolism pathway, and copper ion transport/metabolism pathway are compromised in the liver by two sizes of BSA@Cu_2−x_S NPs, while only SNPs show a complete recovery of altered gene expression after NPs discontinuation. This study demonstrates that the translational feasibility of small-sized BSA@Cu_2−x_S NPs as excellent nanoagents with manageable hepatotoxicity.

**Graphical Abstract:**

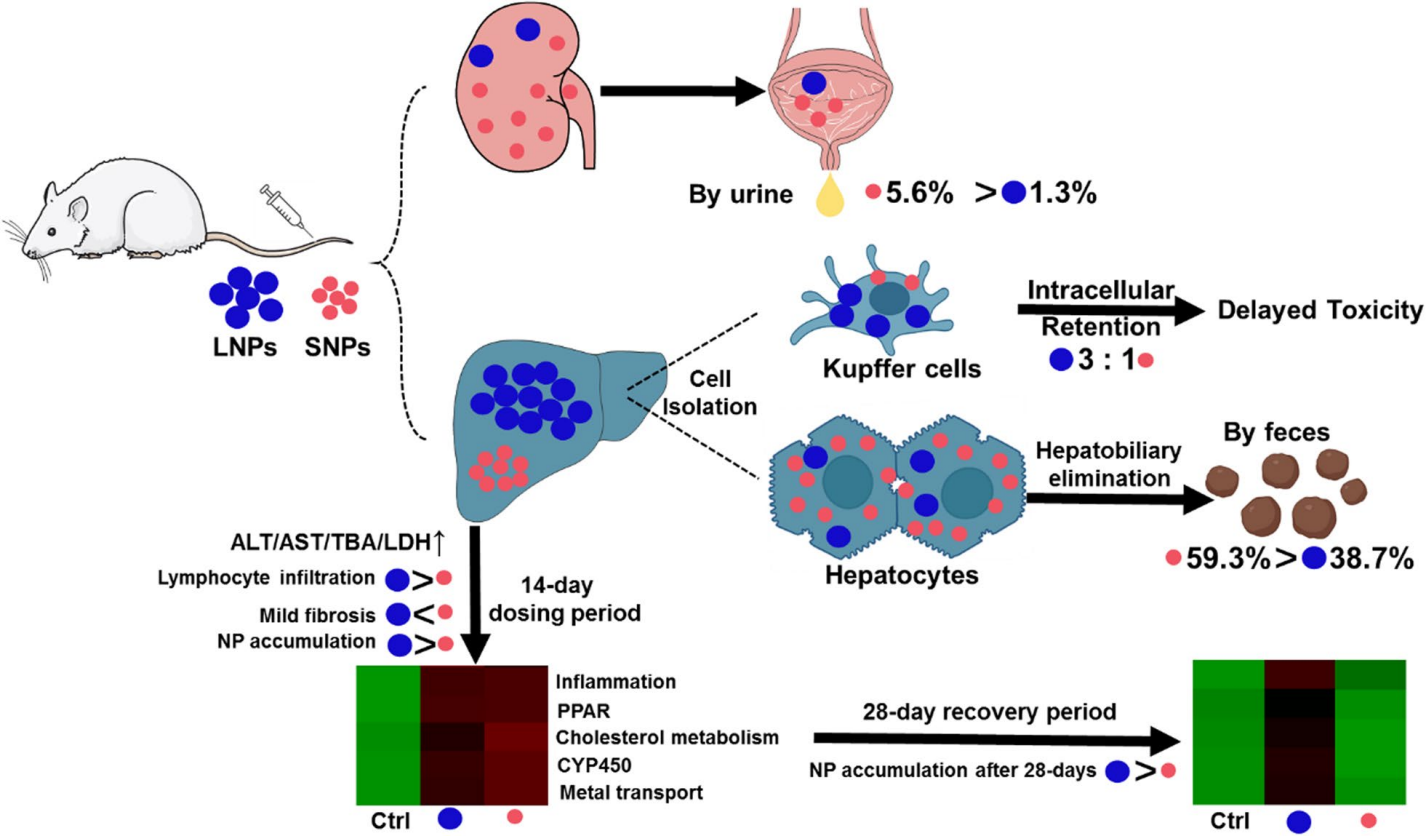

**Supplementary Information:**

The online version contains supplementary material available at 10.1186/s12951-022-01399-5.

## Introduction

Photothermal therapy (PTT) utilizes photothermal transduction agents to convert external light into heat energy, which can raise the intratumoral temperature and trigger cancer cell death [1, 2]. Copper sulfide nanoparticles (Cu_2−x_S NPs, 0 ≤ x ≤ 1) are strong NIR absorbing agents widely recognized in PTT and photoacoustic imaging [3, 4]. Compared to other photothermal agents such as organic dye, gold, silver and carbon nanostructure, Cu_2−x_S NPs have an excellent biocompatibility and photostability [4, 5]. In addition to being used as NIR-I (700–900 nm) agents, Cu_2−x_S NPs can also be as NIR-II (900–1700 nm) agents to facilitate imaging and treatment of deeper lesions, and Cu_2−x_S NPs can flexibly adjust their absorption peak in the NIR region by adjusting the particle size and stoichiometry [6–8]. To date, more than 400 research papers found in Web of Science have been published for Cu_2−x_S NPs and their PTT effects. However, the road to clinical translation of Cu_2−x_S NPs using green controllable preparation methods is still lengthy, mainly limited by detailed preclinical safety evaluation.

Based on the assembly and biomineralization process of albumin, batched albumin-related nanostructures can be facile fabricated for theranostic application of cancer. For instance, Abraxane^®^ formed by the assembly of albumin and paclitaxel has been approved by the FDA and EMA in 2005 and 2008 [9, 10]. Moreover, our group have previously reported a variety of theranostic nanoagents through the albumin-mediated biomineralization process [11]. The synthetic route by biomineralization without introducing any organic solvent or supporting agents suggests an environmentally friendly process. Albumin scaffold meanwhile ensures the chemical stability, physiological stability, dispersibility and biocompatibility of the nanoagents [12–15]. Some studies reported that albumin-biomineralized copper sulfide nanoparticles (BSA@Cu_2−x_S NPs) have excellent photothermal transduction effect, good biocompatibility and dispersibility, as well controllable size of the nanomaterials [16–23]. In 2016, we have shown that BSA@Cu_2−x_S NPs have a high photothermal conversion efficiency, good stability and tumor photothermal ablation capacity [24]. However, the systematic safety evaluation of BSA@Cu_2−x_S NPs has not been further demonstrated so far.

Since the translational potential of Cu_2−x_S NPs for PTT has been highly recognized, only a few researchers noticed safety concerns of Cu_2−x_S NPs and conducted preliminary toxicity studies. For example, one acute toxicity study showed that the maximum tolerated dose and median lethal dose of Cu_2−x_S nanoplates for mice were 8.66 and 54.5 mg/kg, respectively [25]. Rabbits were intravenously administered with ultrasmall Cu_2−x_S nanodots (< 5 nm), which can be completely cleared through feces and urine within 5 days with minimal adverse effects [26]. Due to the limited toxicity profiles of Cu_2−x_S NPs, it is emerging to study Cu_2−x_S NPs regarding distribution, metabolism and pharmacokinetics (DMPK) and long-term toxicity to delineate the size differences.

Herein, we synthesized large-sized (17.8 nm, LNPs) and small-sized (2.8 nm, SNPs) BSA@Cu_2−x_S NPs for comprehensive safety and toxicological evaluation. The criteria for size selection of LNPs and SNPs were based upon the renal filtration threshold of 6 nm; therefore, their profiles of photothermal effects, DMPK and subacute toxicity were compared. To study the DMPK of BSA@Cu_2−x_S NPs, the blood, urine, feces and major organs were collected for Cu content measurement after a single dose of LNPs and SNPs. A single injection of photothermal transduction agents is usually given to the patient to eradicate the tumor [27]. Based upon the requirement from the S3 and M3 guidelines of the international council for harmonisation of technical requirements for pharmaceuticals for human use (ICH), a subacute (14-day repeated dose) toxicity study should be conducted accordingly to support single-dose human trials. Here, rats were intravenously administered BSA@Cu_2−x_S NPs at low, medium, and high doses for 14 consecutive days, which showed variable toxicity that was size- and dose-dependent. We utilized whole transcriptome sequencing to analyze the potential molecular pathways altered by LNPs and SNPs. After the cessation of BSA@Cu_2−x_S NPs administration, the rats subjected to SNPs showed a full recovery compared with LNPs within 1 month. Through this study, we concluded that LNPs and SNPs both caused different degrees of hepatotoxicity, while the hepatotoxicity of SNPs was reversible, suggesting its advantage for clinical translation.

## Materials and methods

### Materials and reagents

Bovine serum albumin (BSA) was purchased from Sinopharm Chemical Reagent Co., Ltd. (Shanghai, China). Copper nitrate and sodium hydroxide were purchased from Aladdin Reagent Co., Ltd. (Shanghai, China). Na_2_S (anhydrous) was purchased from J&K Scientific (Shanghai, China). Copper standard solution was obtained from Beijing North Weiye Metrology Technology Institute (Beijing, China). An enhanced BCA protein assay kit was purchased from Beyotime (Shanghai, China).

### BSA@Cu_2−x_S NPs synthesis and characterization

Cu(NO_3_)_2_·3H_2_O (100 mM used to synthesize SNPs/200 mM used to synthesize LNPs, 10 mL) was slowly added to the stirred solution containing 1.0 g BSA (1.0 g) dissolved in 70 mL deionized water. The reaction mixture was alkalified to pH 12 using 2 M sodium hydroxide. Na_2_S (20 mL, 100 mM used to synthesize SNPs/200 mM used to synthesize LNPs) solution was further added to the above solution and stirred vigorously at 37 °C (used to synthesize SNPs)/55 °C (used to synthesize LNPs) for 12 h. When the reaction was complete, the reaction mixture was purified by dialysis (Mw 3 kDa) for 24 h and lyophilized. By controlling different Cu^2+^ and S^2−^ concentrations and reaction temperatures, we finally obtained large-sized (LNPs) and small-sized (SNPs) BSA@Cu_2−x_S NPs. The morphology of BSA@Cu_2−x_S NPs was observed using a Tecnai G2 spirit BioTwin transmission electron microscope (FEI, America) with a voltage of 120 kV. The hydrodynamic diameters were measured using a Malvern Zetasizer Nano-ZS90 (Malvern instruments, UK). The elements and their valence states were determined by X-ray photoelectron spectroscopy (XPS) (EXCALAB 250 XI, Thermo Scientific). Ultraviolet–visible–near infrared (UV–Vis–NIR) spectra were obtained by using a UV–Vis–NIR spectrophotometer (UV-3600, Shimadzu, Japan). The copper contents of LNPs and SNPs were quantified by ICP–OES (ICAP7200, Thermo Fisher, USA). Different concentrations of BSA@Cu_2−x_S NPs solutions (5, 10, 20, 40, 80 μg/mL Cu) were irradiated under a 1064 nm laser (1 W/cm^2^), and the photothermal effects were determined by an infrared thermal imaging instrument (FLIR, A65). The photoacoustic effects were measured by a photoacoustic tomography imaging system (MSOT inSight/inVision 256).

### Pharmacokinetics and biodistribution of BSA@Cu_2−x_S NPs in SD rats

SD rats (180–200 g) were purchased from Caven’s laboratory animal company (Changzhou, China), kept in an environment of 21 ± 1 °C and 55 ± 5% humidity and supplemented under artificial light, with a daily cycle of 12 h. The rats were supplied with standard food pellets and tap water. The experimental procedures were performed in accordance with the National Institute of Health Guide for the Care and Use of Laboratory Animals and carried out under the Animal Ethics Committee of Soochow University. All efforts are to alleviate the suffering of animals and reduce the number of animals.

For pharmacokinetic studies, SD rats were randomly divided into LNPs, SNPs and a control group (3 rats per group). Rats were administered NPs at a single dose through the tail vein with a dose equivalent to 5 mg/kg Cu (5 mg/kg). Blood samples were collected successively at 10 min, 30 min, 1 h, 2 h, 4 h, 8 h, 12 h, 24 h, 48 h, 72 h, 120 h, and 168 h after injection and digested with a mixture of HCl/HNO_3_ (3:1, v/v) to quantify Cu contents by ICP-MS (ELEMENT 2, Thermo, America). Pharmacokinetic-related parameters of LNPs and SNPs were analyzed by using a Micro IV Bolus two-compartmental model embedded in WinNonlin8.1.0. Urine and fecal samples were collected at 0–12 h, 12–24 h, 24–48 h, 48–72 h, 72–120 h, 120–168 h and digested by a mixture of HCl/HNO_3_ (3:1, v/v) for Cu content analysis using ICP-OES. For biodistribution analysis, SD rats were divided into the abovementioned three groups with three rats per group at different time points. After NPs injection, rats were sacrificed at 2 h, 6 h, 12 h, 24 h, 72 h, and 168 h, and their major organs, including the heart, liver, spleen, lung, and kidney, were collected and digested with a mixture of HCl/HNO_3_ (3:1, v/v), followed by the quantification of Cu contents using ICP-OES.

### Subacute study and recovery period design for BSA@Cu_2−x_S NPs

SD rats were administered LNPs and SNPs at different doses (2, 5, 8 mg/kg) and a recovery group at 8 mg/kg, along with a control group. These groups were briefly named L2, L5, L8, and L8-R and S2, S5, S8, S8-R and Ctrl. Specifically, rats (six rats per group) were injected with LNPs and SNPs saline solution via intravenous injection daily for 14 consecutive days and sacrificed thereafter. Rats in the recovery groups were administered BSA@Cu_2−x_S NPs at a dose of 8 mg/kg daily for 14 days but were allowed to recover for 28 days after cessation of NPs exposure and sacrificed at the end of the recovery period. Rats in the control group were administered saline solution and sacrificed immediately after the dosing and recovery period. The body weight of the rats was recorded daily until sacrifice, and at the end of the dosing period (Day 14) or recovery period (Day 14 + 28, 42), all the rats were sacrificed for blood and tissue collection. Specifically, the organs were quickly collected, washed with physiological saline, blotted with filter paper, and weighed for organ coefficient calculation.

### Serum biochemistry, hematological analysis and H&E staining

Blood was collected and inverted for mixing in a coagulant tube containing the separating gel, leaving it at 4 °C for 30 min. Blood was centrifuged at 3000 rpm/10 min for serum collection and biochemical analysis. The levels of alanine transaminase (ALT), aspartate transaminase (AST), total bile acid (TBA), albumin (ALB), and lactate dehydrogenase (LDH) were determined. Hematological parameters were measured using whole blood stored in EDTA-based anticoagulant tubes. At the end of the animal experiment, the heart, liver, spleen, lung and kidneys were dissected and preserved in formalin. The samples were paraffin embedded and cut into ~ 5 μm sections for H&E staining procedures. The histological sides were carefully and unbiasedly diagnosed by an expert pathologist.

### Cellular uptake study of LNPs and SNPs in isolated hepatocytes and Kupffer cells

Primary rat hepatocytes and Kupffer cells were isolated by a two-step procedure and purified with density gradient centrifugation. Specifically, the liver was digested by collagenase IV, and the obtained cells were centrifuged at low speed and purified by Percoll solution for dead cell/cell debris removal. The supernatant was collected, and Percoll solutions at 25% and 50% were used for Kupffer cell purification. Hepatocytes or Kupffer cells were incubated with LNPs or SNPs at 10 μg/mL in DMEM culture medium at 37 °C for 6 h. After incubation, cells were collected and washed in ice-cold PBS and digested with a mixture of HCl/HNO_3_ (3:1, v/v) to quantify Cu contents by ICP-MS. Hepatocytes were also cultured into 3D spheroids for hepatocyte uptake analysis, according to a previously reported method [28]. A metal mold for culturing 3D hepatocyte spheroids was designed by our group (data to be published), which was used to make an agarose sheet with a pit array in one well of 6-well plates. Impact hepatocyte spheroids can be formed on the bottom of each pit in the agarose sheet within 72 h after adding the cell suspension into the 6-well plate.

### RNA sequencing of livers from BSA@Cu_2−x_S NPs-treated rats

The liver tissues of the rats from the control and dosing groups (L8 or S8 group dosed at 8 mg/kg for 14 consecutive days) were outsourced to Lianchuan Bio Technology Co., Ltd. (Hangzhou, China) for RNA-sequencing analysis. Total RNA of each sample was isolated and purified using TRIzol reagent (Invitrogen, Carlsbad, CA, USA) following the supplier’s instructions. The RNA amount and purity were quantified using a NanoDrop ND-1000 (NanoDrop, Wilmington, DE, USA). RNA integrity was assessed by a Bioanalyzer 2100 (Agilent, CA, USA). The RNA was reverse-transcribed to generate cDNA by SuperScript™ II Reverse Transcriptase (Invitrogen, cat. 1896649, USA), and then the cDNA was amplified with PCR under the following conditions: initial denaturation at 95 °C for 3 min; 8 cycles of denaturation at 98 °C for 15 s, annealing at 60 °C for 15 s, and extension at 72 °C for 30 s; and then final extension at 72 °C for 5 min. The average insert size for the final cDNA library was 300 ± 50 bp. Finally, 2 × 150 bp paired-end sequencing (PE150) was performed using an Illumina NovaSeq™ 6000 (Lianchuan Bio Technology CO., Ltd., Hangzhou, China) following the manufacturer’s protocol. FASTP, HISAT2 and StringTie software were used for quality control of samples, comparison with databases, and FPKM quantification of genes, respectively. The differentially expressed mRNAs were selected with fold change > 2 or < 0.5 and with parametric F test (p value < 0.05) by R package edgeR. Finally, DAVID software was used to perform KEGG enrichment analysis on genes. Genes usually interact with each other to play roles in certain biological functions. Pathway-based analysis helps to further understand gene biological functions. KEGG is the major public pathway-related database. Pathway enrichment analysis identified significantly enriched metabolic pathways or signal transduction pathways in DEGs compared with the whole genome background.

### RT-qPCR analysis for assessing toxicity reversibility by LNPs and SNPs

The changes in mRNA expression were evaluated by RT-qPCR in the liver tissue from BSA@Cu_2−x_S NPs and the corresponding recovery groups. Total RNA was extracted from the livers of rats treated with 8 mg/kg LNPs and SNPs at the end of the dosing period (Day 14) and recovery period (Day 42) using a SteadyPure Universal RNA Extraction Kit (Accurate Biotechnology, Hunan, China) according to the supplier’s instructions. RNA was reverse transcribed into cDNA using an Evo M-MLV reverse transcription kit (Accurate Biotechnology, Changsha, China) carried out by a gradient PCR machine (Tprofessional Thermocycler, Biometra, Germany). The reaction program was set up as follows: 37 °C for 15 min, 85 °C for 5 s, and cool down to 4 °C. Real-time quantitative PCR (RT-qPCR) was performed using a SYBR Green Pro Taq HS premix kit (Accurate Biotechnology, Changsha, China) on an ABI ViiA7 PCR machine (Applied Biosystems, Life Technologies, USA). The thermocycler parameters were set up as follows: 95 °C for 30 s, 40 cycles of 95 °C for 5 s and 60 °C for 30 s. Ct values were used to analyze the difference between the dosing group and the control group. All primers used in this study are listed in Additional file 1: Table S1.

### Statistical analysis of data

Data are expressed as the mean ± standard deviation (Mean ± SD). Statistical significance was assessed using one-way ANOVA, and a probability value of p < 0.05 was considered statistically significant. *p < 0.05, **p < 0.01, ***p < 0.001.

## Results and discussion

### Synthesis and characterization of BSA@Cu_2−x_S NPs

**Fig. 1:**
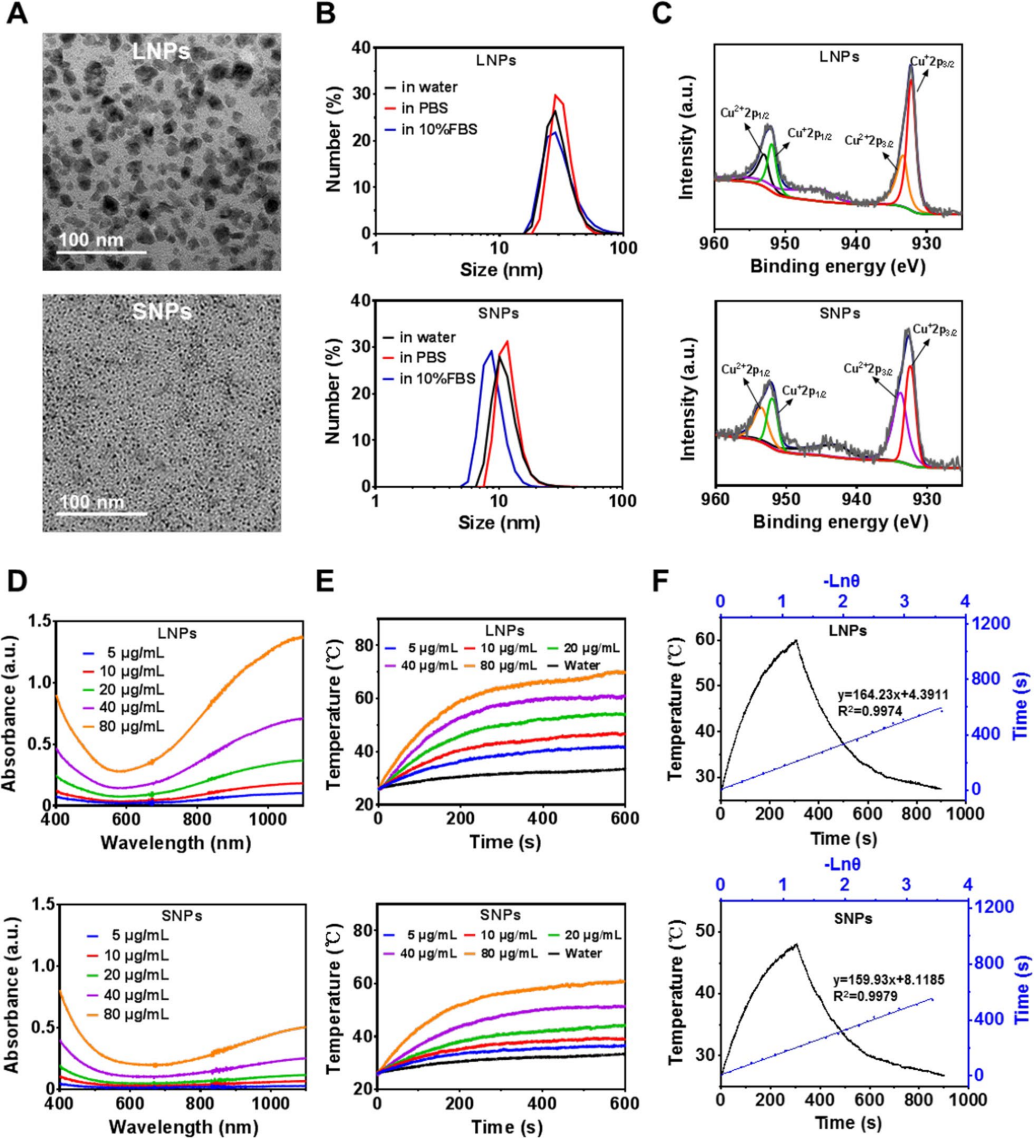
Physicochemical characterization of BSA@Cu_2−x_S NPs. It is noted that “LNPs” stands for large-sized BSA@Cu_2−x_S NPs and “SNPs” for those with small-sizes. **A** TEM images for LNPs and SNPs. **B** The hydrodynamic sizes of both NPs in water, PBS and 10% FBS by DLS measurement. **C** XPS spectra for LNPs and SNPs. **D** UV–vis–NIR spectra of both NPs at different concentrations (equivalent to 5, 10, 20, 40, 80 μg/mL Cu). **E** Photothermal heating curves of both NPs at different concentrations under 1064 nm laser irradiation with a power density of 1 W/cm^2^. **F** Photothermal heating and natural cooling curve at a dose of 40 μg/mL (black line) and linear relationship between − ln*θ* (negative natural logarithm of the temperature driving force) and time in the cooling stage (blue line)

BSA was determined as the template to synthesize BSA@Cu_2−x_S NPs due to its excellent biocompatibility and stability. The ratio between BSA and copper ions can be flexibly adjusted to obtain variable NPs sizes. To investigate the size differences for the biocompatibility and toxicity of BSA@Cu_2−x_S NPs, we synthesized large- and small-sized NPs, named LNPs and SNPs, respectively. The transmission electron microscopy (TEM) image in Fig. 1A depicts the amorphous morphology and the sizes of LNPs (17.8 nm) or SNPs (2.8 nm). The hydrodynamic sizes of LNPs and SNPs were 28.2 nm (PDI at 0.24) and 10.1 nm (PDI at 0.22) in water, respectively, with no significant size changes in other media (Fig. 1B). Zeta potential analysis shows values of − 34.9 mV and − 34.6 mV for LNPs and SNPs, indicating their similar surface properties with high stability (< − 30 mV). BSA@Cu_2−x_S NPs were stable in an aqueous solution for 7 consecutive days, with no significant change in size (Additional file 1: Fig. S1C). X-ray photoelectron spectroscopy (XPS) was utilized to explore the valence states of copper elements in BSA@Cu_2−x_S NPs, and the results revealed that Cu^+^ and Cu^2+^ were both available in LNPs and SNPs (Fig. 1C).

We further investigated the NIR absorbance and photothermal properties of BSA@Cu_2−x_S NPs and NPs of two sizes were compared. The UV–vis–NIR absorbance spectra depict that both NPs have strong absorption in the NIR range, especially in NIR-II (> 900 nm) (Fig. 1D). It was noted that LNPs had a stronger absorption in NIR-II than SNPs at the same copper concentration. The photothermal properties of BSA@Cu_2−x_S NPs were measured by photothermal heating curves and recorded by infrared thermography. The results show a concentration-dependent photothermal effect for both BSA@Cu_2−x_S NPs, although LNPs exhibit better photothermal characteristics than small-sized NPs (Fig. 1E and Additional file 1: Fig. S1A). The heating and cooling curves used for the calculation of photothermal conversion efficiency (PCE) are similar for LNPs (42.7%) and SNPs (44.9%) based upon an energy balance model previously described (Fig. 1F) [11, 29]. To investigate the photothermal stability of the BSA@Cu_2−x_S NPs, five consecutive “ON–OFF” cycles of LNPs or SNPs under a 1064 nm laser (1 W/cm^2^) were conducted. The temperature changes of all five cycles had no significant differences, suggesting the excellent stability of BSA@Cu_2−x_S NPs (Additional file 1: Fig. S1B). Other than photothermal properties, both BSA@Cu_2−x_S NPs showed a photoacoustic response (Additional file 1: Fig. S1D). In summary, the above results indicate that the photothermal heating ability and photoacoustic signal of LNPs are higher than those of SNPs, while PCEs are similar for both BSA@Cu_2−x_S NPs. It should be emphasized that the PCEs of our synthesized BSA@Cu_2−x_S NPs are higher than those of existing NIR-II agents [30].

### Pharmacokinetics and biodistribution of LNPs and SNPs in SD rats

**Fig. 2:**
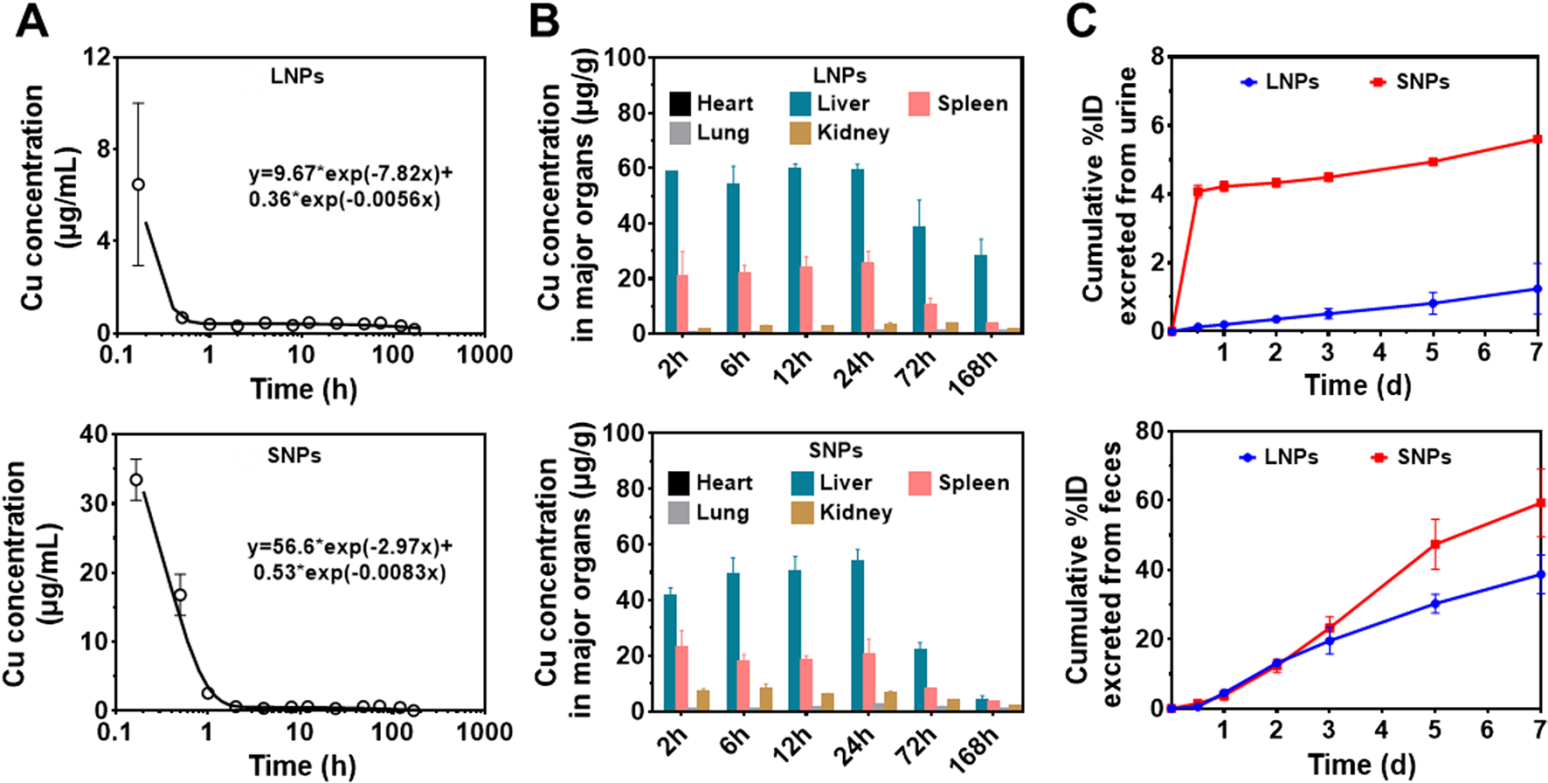
The pharmacokinetics, biodistribution and elimination of LNPs and SNPs. The rats were administered BSA@Cu_2−x_S NPs of two different sizes by a single-dose intravenous administration (5 mg/kg Cu). **A** The pharmacokinetic profile of LNPs and SNPs in the blood. **B** The biodistribution of both NPs in five major organs at different time points up to 168 h (1 week). **C** Cumulative excretion of LNPs and SNPs into urine and feces. Values are represented as the mean ± SD

To compare the size differences for the pharmacokinetic profile of BSA@Cu_2−x_S NPs in SD rats, blood was collected, and the copper element was quantified by ICP-MS at different time points after a single intravenous (IV) dose via the tail vein. The blood distribution half-life (t_1/2α_) was 5.3 min and 13.8 min for LNPs and SNPs, respectively, while the blood terminal elimination half-life (t_1/2β_) of LNPs and SNPs was 124.12 h and 83.85 h, respectively, which suggests the rapid tissue distribution and delayed metabolism/elimination of LNPs compared with SNPs (Fig. 2A). All the other pharmacokinetic parameters are listed in detail in Additional file 1: Table S2, with the lower value of the area under the blood activity time curve, the higher volume of distribution, and the higher mean residence time of LNPs, confirming their quick accumulation and slow removal in the tissues compared to SNPs.

To further understand the ADME profile of BSA@Cu_2−x_S NPs, the biodistribution of LNPs and SNPs in major organs at different time points was investigated through copper quantification. The accumulated amount of LNPs and SNPs in the major organs was liver > spleen > kidney > lung > heart. In contrast, no copper content was detected in the heart (Fig. 2B). It should be noted that LNPs accumulated in the liver was significantly higher than that of SNPs, and LNPs remained in the liver with a large amount, while SNPs were mostly removed from the liver to the basal level 168 h (7 days) after the single IV dose. The urine and feces were collected consecutively during the 7 days after administration. It seems that a minority of SNPs (5.6% of the total amount administered) were excreted rapidly from urine, and most of the SNPs (59.34%) were cleared in the feces within 1 week, while only 1.25% and 38.7% of LNPs were removed by urine and feces, respectively (Fig. 2C). These results highly illustrated that BSA@Cu_2−x_S NPs were mainly excreted through the hepatobiliary pathway, with more removal for SNPs than LNPs.

### Gross pathology findings for the subacute studies of LNPs and SNPs in SD rats

**Fig. 3:**
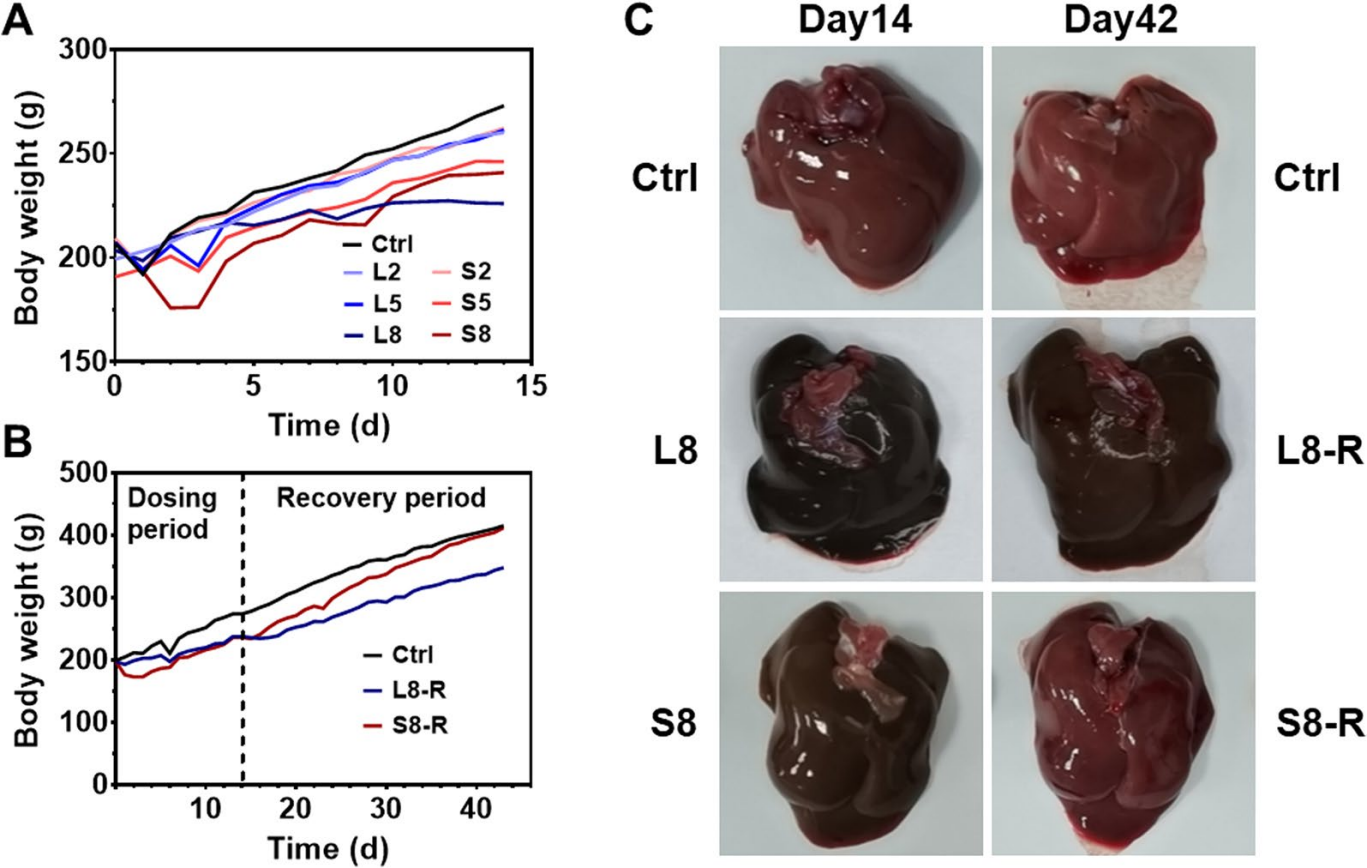
Body weight and rat livers in the subacute toxicity study by LNPs and SNPs. Rats were subjected to LNPs or SNPs daily for 14 consecutive days followed by a 28-day recovery period. **A** Body weight growth profiles of the rats from the control group and dosing groups treated with LNPs or SNPs. Rats were IV administered LNPs (L2, L5, L8) and SNPs (S2, S5, S8) at 2, 5, and 8 mg/kg for 14 consecutive days. **B** Body weight growth profiles of the rats from the control group and recovery groups. Here, rats were subjected to LNPs (L8-R) or SNPs (S8-R) at 8 mg/kg daily for 14 days followed by a 28-day recovery period. **C** The liver morphology of the rats from the control group (Ctrl), dosing groups (L8 or S8) and recovery groups (L8-R or S8-R)

The dosage regimen for photothermal therapy of NIR agents currently available is usually designed by a single-dose administration in clinical practice [27]. According to ICH M3 and S3 guidelines, the rats were IV administered BSA@Cu_2−x_S NPs daily for 14 consecutive days, aiming to delineate the general toxicity profile that can support the investigative new drug application (IND) of copper sulfide-based NPs. Our preliminary studies have shown that the maximum tolerated dose of BSA@Cu_2−x_S NPs was 8 mg/kg, showing fatigue, anorexia, hypoactivity and blood trails under the nostrils of the treated rats. As a result, we selected 2, 5 and 8 mg/kg as the low (LNPs and SNPs at 2 mg/kg, abbreviated as L2 and S2, respectively), medium (L5 and S5) and high doses (L8 and S8), respectively. The body weight (BW) was recorded during the subacute toxicity study, showing a relative decrease in BW in a dose-dependent manner (Fig. 3A). A marked decrease in BW within three days was found in the S8 group, but this stress response seemed to be tolerated 3 days later by BW recovery. In comparison, the BW in the L8 group remained suppressed during the entire repeated dosing period.

BSA@Cu_2−x_S NPs administration was discontinued on day 14 followed by an extra 28-day recovery period (L8-R or S8-R group, total 42 days of observation) to investigate the reversibility of the NPs. Interestingly, rat BW in the S8-R group rapidly increased after the cessation of NPs injection and fully recovered to the same weight as the control group at day 42, while the rat BW in the L8-R group only showed a mild increase when LNPs were discontinued (Fig. 3B).

The weights of the internal organs were also measured, and the organ coefficient data showed no relative weight increase in the liver, kidney, lung or heart, while the spleen weight significantly increased and could be recovered in the S8-R group but not in the L8-R group, again supporting the toxicity reversibility of small-sized BSA@Cu_2−x_S NPs (Additional file 1: Fig. S2). Since the liver was determined to be the main organ for NPs distribution, the livers were collected and observed for gross pathology findings. As shown in Fig. 3C, the liver color of the rats treated with LNPs (L8) and SNPs (S8) for 14 days changed from normal bright red to dark green (darker in the L8 group than in the S8 group), which was close to the color of the BSA@Cu_2−x_S NPs, implying a large amount of NPs accumulation in the liver. However, it is optimistic to find that the rat liver in S8-R group was recovered to a normal and bright red color after 28 days of the recovery period, highly suggesting the efficient clearance of SNPs and reversible health status of SNPs-treated liver, in comparison, the liver color of L8-R group remained dark, mostly because of the slow clearance of LNPs in the liver.

### Hematology and blood chemistry studies for LNPs and SNPs

**Fig. 4:**
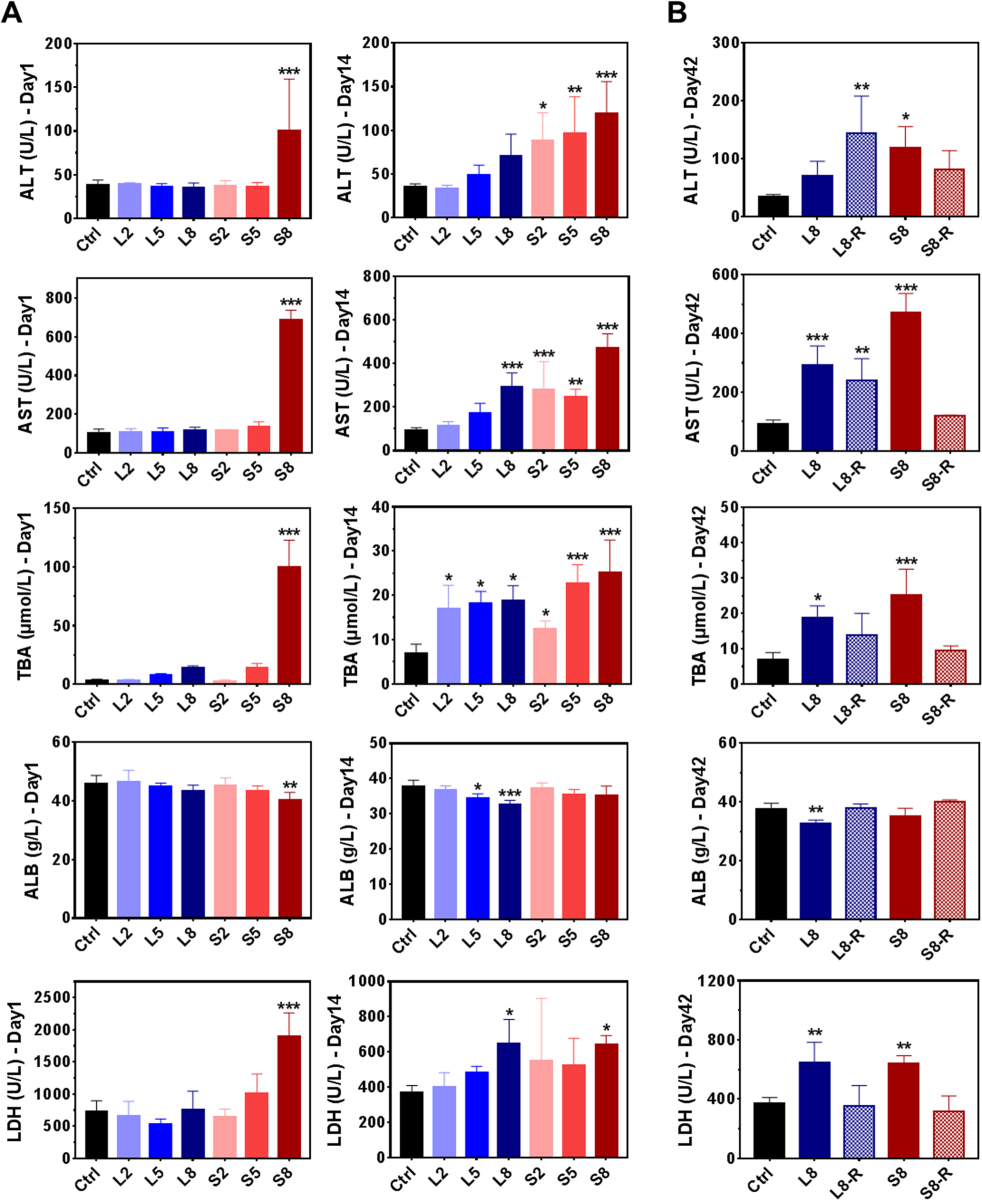
Serum biochemical parameters of the rats treated with LNPs and SNPs at the end of the dosing period and recovery period. Rats were dosed with LNPs and SNPs daily for 14 consecutive days followed by a 28-day recovery period, and blood was collected for serum biochemistry analysis, including ALT, AST, TBA, ALB and LDH, which are related to hepatotoxicity. **A** The serum levels of indicators for liver injury at Day 1 and Day 14 (the last day of the dosing period). **B** The serum levels of the 8 mg/kg dosing groups at day 14 (L8 and S8) and recovery groups at the end of the recovery period (L8-R and S8-R, “R” indicates the recovery group 28 days after the discontinuation of NPs administration). Values are represented as the mean ± SD. Statistical significance was assessed using one-way ANOVA (significance versus control: *p < 0.05, **p < 0.01, ***p < 0.001). *ALT* alanine aminotransferase, *AST* aspartate aminotransferase, *TBA* total bile acid, *ALB* albumin, *LDH* lactic dehydrogenase

Intravenous blood was collected for hematological and biochemical tests from the rats treated daily with BSA@Cu_2−x_S NPs for 14 days. Other than the fluctuation of white blood cells from LNPs-treated rats showing statistically significant differences from controls, most hematological indicators showed no distinct changes, and they were all within the normal ranges for both NPs (Additional file 1: Fig. S3). In comparison, blood biochemical studies showed a differential profile for LNPs and SNPs that were highly dependent upon administration duration. Specifically, the serum levels of ALT, AST, TBA and LDH in the S8 group were increased, and the ALB level decreased significantly at day 1, suggesting the rapid stress response of rat liver when subjected to a high dose of SNPs. After 14 consecutive days of administration, the levels of ALT, AST, TBA and LDH all increased in a dose-dependent manner by both LNPs and SNPs, while the ALB level was decreased by LNPs only, suggesting the functional alteration of the liver uniquely by LNPs (Fig. 4A).

Toxicity reversibility is a highly recognized study that must be submitted to regulatory agencies to demonstrate that drug toxicity can be reversed and managed by the discontinuation of the drug. NPs administration was discontinued for surveillance of blood chemistry parameters at the end of the 28-day recovery period. The ALT level after 14 days of LNPs treatment at 8 mg/kg (70.8 U/L for L8) continued to increase to 143.9 U/L after recovery for 28 days (L8-R group), while the ALT value decreased from 119.0 to 81.3 U/L for small-sized NPs (Fig. 4B). With a similar trend, the AST level in the L8-R group remained 2.6-fold higher than that in the control group after the recovery period, while the AST value in the S8-R group decreased from a significantly high level (a 5.1-fold increase over the control for the S8 group) to almost the control value. The failure of recovery of total bile acid (TBA) also occurred with LNPs but not SNPs. These optimistic data strongly indicate that SNPs toxicity can be fully reversed by NPs discontinuation, while LNPs exhibit delayed toxicity even after the cessation of administration. It should be noted that neither LNP nor SNP would cause renal function injury by comparing the serum level of CREA and UA in control and dosing groups during the 14 consecutive days of administration (Additional file 1: Fig. S4).

### Histopathological features of the liver by LNPs and SNPs

**Fig. 5:**
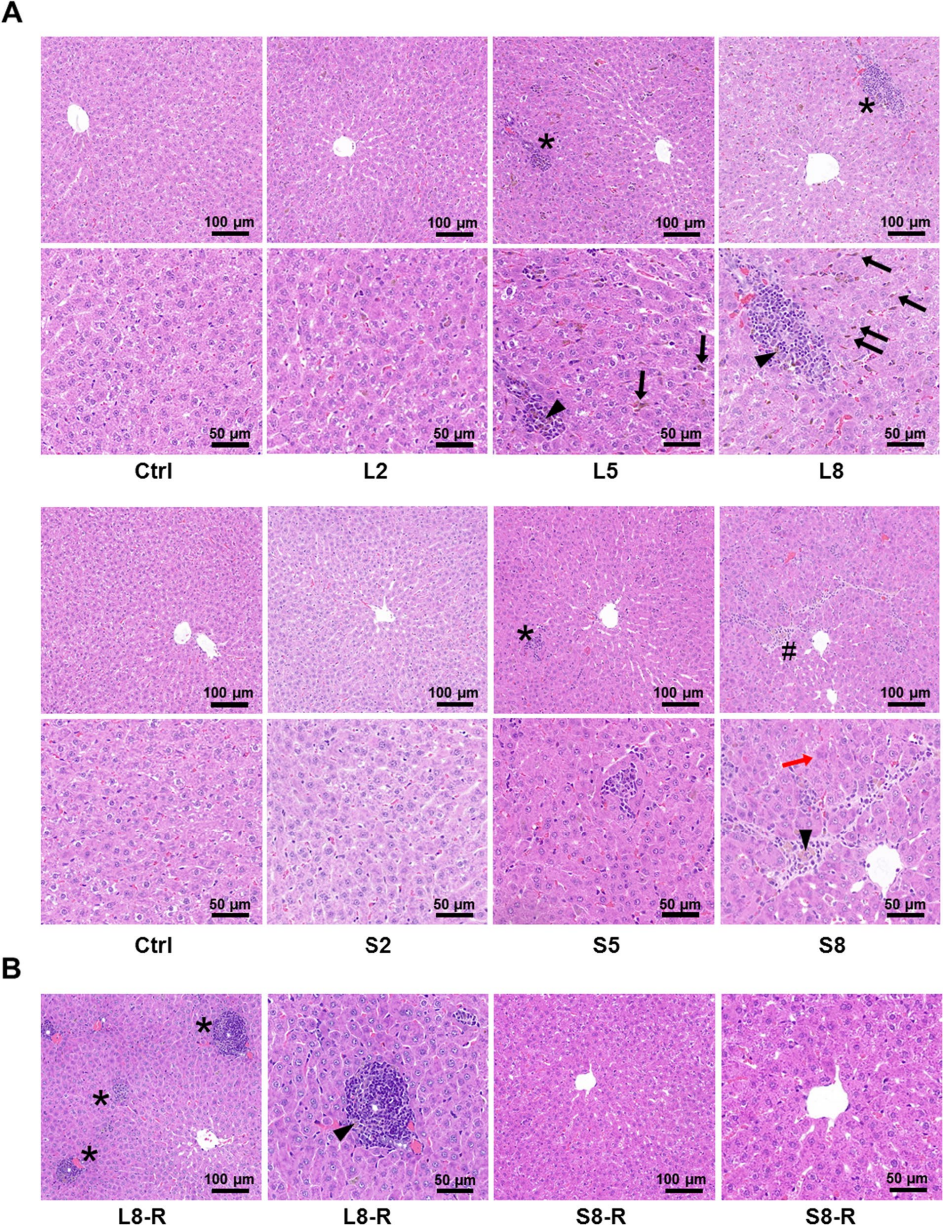
Histopathological review of rat livers by LNPs and SNPs during the dosing and recovery periods. **A** H&E staining of the livers at the end of the dosing period. Rats were subjected to LNPs (L2, L5, L8) or SNPs (S2, S5, S8) at 2, 5, and 8 mg/kg for 14 consecutive days. Hepatic sinusoid expansion and hepatocyte polarity disorder were common in all treated samples. Focal lymphocyte infiltration (*) with brown-colored NPs deposition (arrowhead) was found to be more severe, with larger areas in LNPs livers than those by SNPs. LNPs can be distinctly observed with brown color in the individual Kupffer cells at the end of the dosing period (→). Slight lymphocyte infiltration along with mild fibrosis (#) and focal necrosis (red arrow) was found in the SNPs-treated groups. **B** H&E staining of rat livers at the end of the recovery period. LNPs- and SNPs-treated groups were allowed to recover for 28 days after the discontinuation of BSA@Cu_2−x_S NPs and were named the L8-R and S8-R groups. Severe LNPs-induced lymphocyte infiltration in the liver remained at the end of the recovery period, while the liver fully recovered after SNPs discontinuation

To further delineate the hepatotoxicity of large-sized (LNPs) and small-sized (SNPs) BSA@Cu_2−x_S NPs, the livers from the dosing and recovery groups were H&E stained and observed. In the control samples, the hepatocytes were radially arranged connecting the portal veins, while this cord shape became obscure by the low dose of LNPs (L2). At higher doses of LNPs (L5), several pathological alterations were evident, including hepatic sinusoid expansion, hepatocyte polarity disorder and focal lymphocyte infiltration (*). It is noted that the area for cell infiltration was enlarged in the L8 group, suggesting severe inflammation by the high dose of LNPs (Fig. 5A). LNPs at 5 and 8 mg/kg (L5 and L8) were found to be deposited in Kupffer cells, showing brown staining of the cells (arrows). LNPs may also deposit (arrowhead) in the cells of the inflammatory site, with the amounts of NPs highly correlating with the degree of lymphocyte infiltration.

In comparison, the pathological changes in the liver caused by SNPs showed a distinguished pattern. The low dose of SNPs (S2) introduced slight and focal hepatocyte swelling, while the hepatic cell cords remained organized. With the increase in the dose at 5 mg/kg (S5), cell infiltration sites with a small cluster of lymphocytes (*) were sporadically observed. Focal necrosis became evident (red arrow) in the highest dosing group (S8), along with the formation of fibrous connective tissue (#) (Fig. 5A). SNPs deposition in the liver was minimal in the low (S2) and medium (S5) dosing groups, while SNPs dispersively accumulated (arrowhead) in the fibrotic sites of the livers when rats were treated at a high dose (S8). Therefore, unlike LNPs, which promote a high degree of inflammatory infiltration and intra-Kupffer accumulation of NPs, SNPs are prone to hepatocyte necrosis and hepatic fibrosis.

The liver from rats treated with BSA@Cu_2−x_S NPs showed an optimistic recovery in SNPs compared with LNPs. Specifically, the lymphocyte infiltration area became further enlarged despite the discontinuation of LNPs, and LNPs (shown as brown deposition) remained accumulated within the inflammatory area during the 28-day recovery period (Fig. 5B). In comparison, it is interesting that hepatocyte necrosis and fibrosis caused by SNPs at 8 mg/kg were fully repaired at the end of the recovery period, showing the normal microstructure of the liver (Fig. 5B). In addition, no SNPs deposition was found in the histological sections of the S8-R group.

Based upon the above observation, dose-dependent liver injury was found in both LNPs and SNPs after 14 consecutive days of administration, and the histopathological profile of LNPs appeared to be more severe than that of SNPs. The damaged sites could have a full recovery in the livers of SNPs-treated rats, while the livers of LNPs-treated rats remained inflammatory with NPs retention. In comparison, the other major organs did not have distinct toxicological changes other than reversible spleen toxicity (Additional file 1: Fig. S5).

### Uptake studies of liver and cells by LNPs and SNPs

**Fig. 6:**
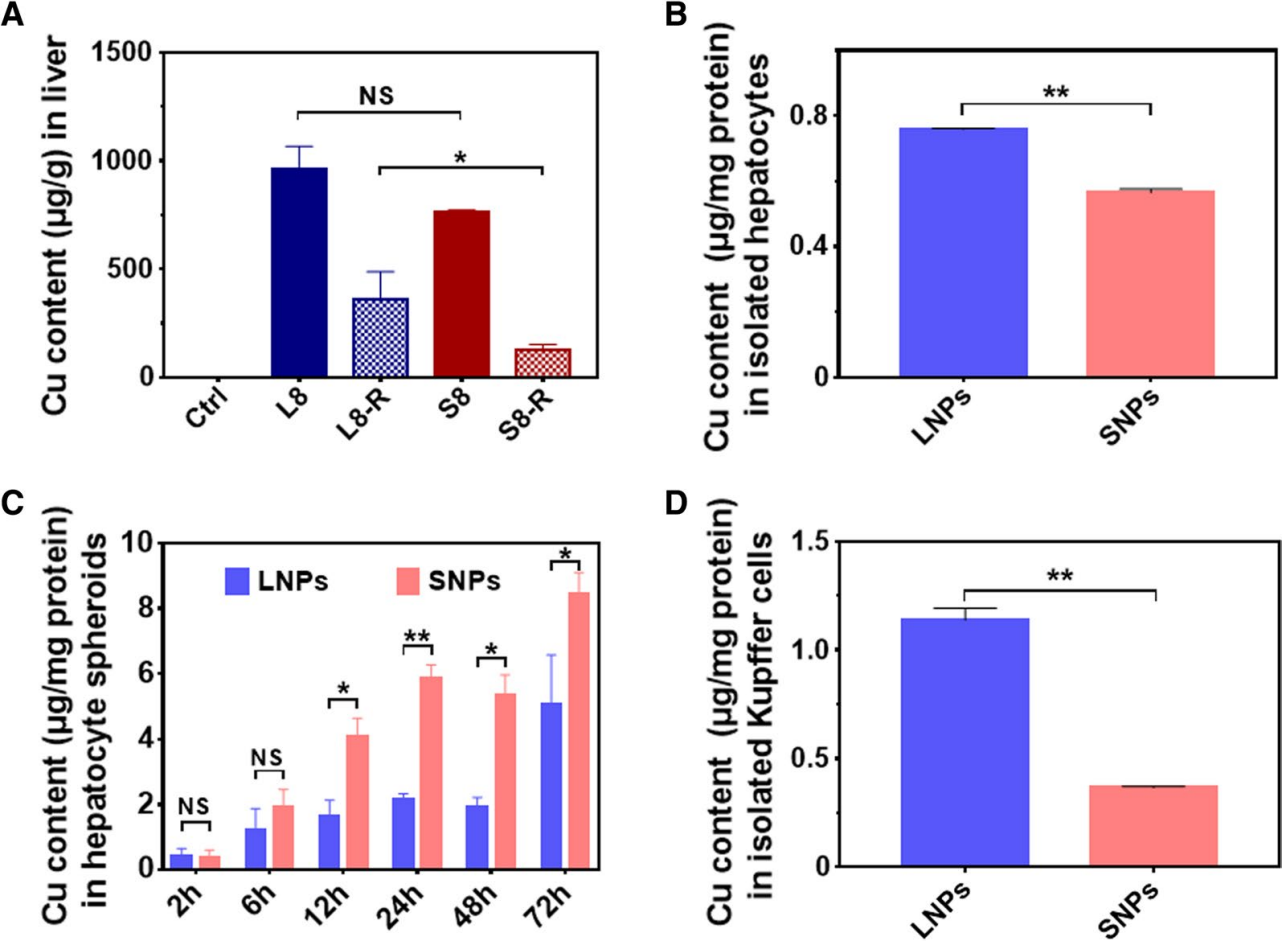
BSA@Cu_2−x_S NPs accumulation in the liver and cellular uptake study of NPs in isolated hepatic cells. **A** NPs accumulation in rat livers treated with 8 mg/kg LNPs and SNPs after the dosing and recovery period. **B** Freshly isolated hepatocytes were incubated with 10 μg/mL LNPs and SNPs for 6 h. **C** Uptake of LNPs and SNPs in rat primary hepatocyte spheroids at different time points. **D** Freshly isolated Kupffer cells were incubated with 10 μg/mL LNPs and SNPs for 6 h. Cu content was quantified by ICP-MS. Values are represented as the mean ± SD. Statistical significance was assessed using one-way ANOVA (NS: no significance, *p < 0.05, **p < 0.01)

Previous DMPK and toxicity studies demonstrated that hepatotoxicity may be associated with the retention of BSA@Cu_2−x_S NPs in the liver. Therefore, we quantified the copper contents of the livers from the dosing group (L8 and S8) and recovery group (L8-R and S8-R) by ICP-OES. The copper element was accumulated in the rat livers of the S8 groups at a high level of 760 μg/g after SNPs treatment for 14 consecutive days, while the copper content was significantly reduced to 128 μg/g (S8-R), namely, a 6.0-fold reduction at the end of the recovery period (Fig. 6A). In contrast, large-sized NPs were accumulated in the liver with a higher amount (960 μg/g) than SNPs after 2 weeks of the dosing period, and the remaining Cu element in the L8-R group after 28 days of recovery was 358 μg/g, which was 2.8-fold higher than that in the S8-R group.

The high uptake and slow clearance of copper elements by LNPs in the liver along with pathological findings imply the relative selectivity of LNPs by Kupffer cells, which are known as a specific type of macrophage responsible for the cellular uptake of exogenous substances/particles and hepatic inflammation [31, 32]. To further prove this hypothesis, primary rat hepatocytes and Kupffer cells were isolated and purified from rat liver and incubated with LNPs or SNPs for 6 h, aiming to investigate their uptake differences. After incubation of BSA@Cu_2−x_S NPs with rat hepatocytes, the intracellular Cu content in LNPs (0.76 μg/mg cells)-treated cell samples was comparable to that of cells treated with SNPs (0.56 μg/mg cells), as shown in Fig. 6B. To recapitulate the actual microstructure in the liver, primary rat hepatocytes were cultured into three-dimensional hepatocyte spheroids according to our in-house method. Approximately 1000 hepatocyte spheroids equivalent to 1 million cells were obtained for incubation with LNPs and SNPs at different time points followed by ICP-MS detection of Cu content. As shown in Fig. 6C, the uptake of BSA@Cu_2−x_S NPs by hepatocyte spheroids increased in a time-dependent manner, and the amount of SNPs uptake by hepatocyte spheroids was generally > two folds (2.8-fold at 24 h) that of LNPs, highly indicating the preferred SNPs uptake by hepatocytes. In contrast, Kupffer cells were prone to the uptake of larger-sized NPs, showing a much higher (3.1-fold) Cu content in LNPs (1.14 μg/mg cells) than SNPs (0.37 μg/mg cells) (Fig. 6D). Therefore, we herein suggest that hepatocytes in the liver may take up small-sized BSA@Cu_2−x_S NPs, potentially introducing rapid clearance, while Kupffer cells selectively take up large-sized NPs and retain them for a long period.

### RNA sequencing analysis

**Fig. 7:**
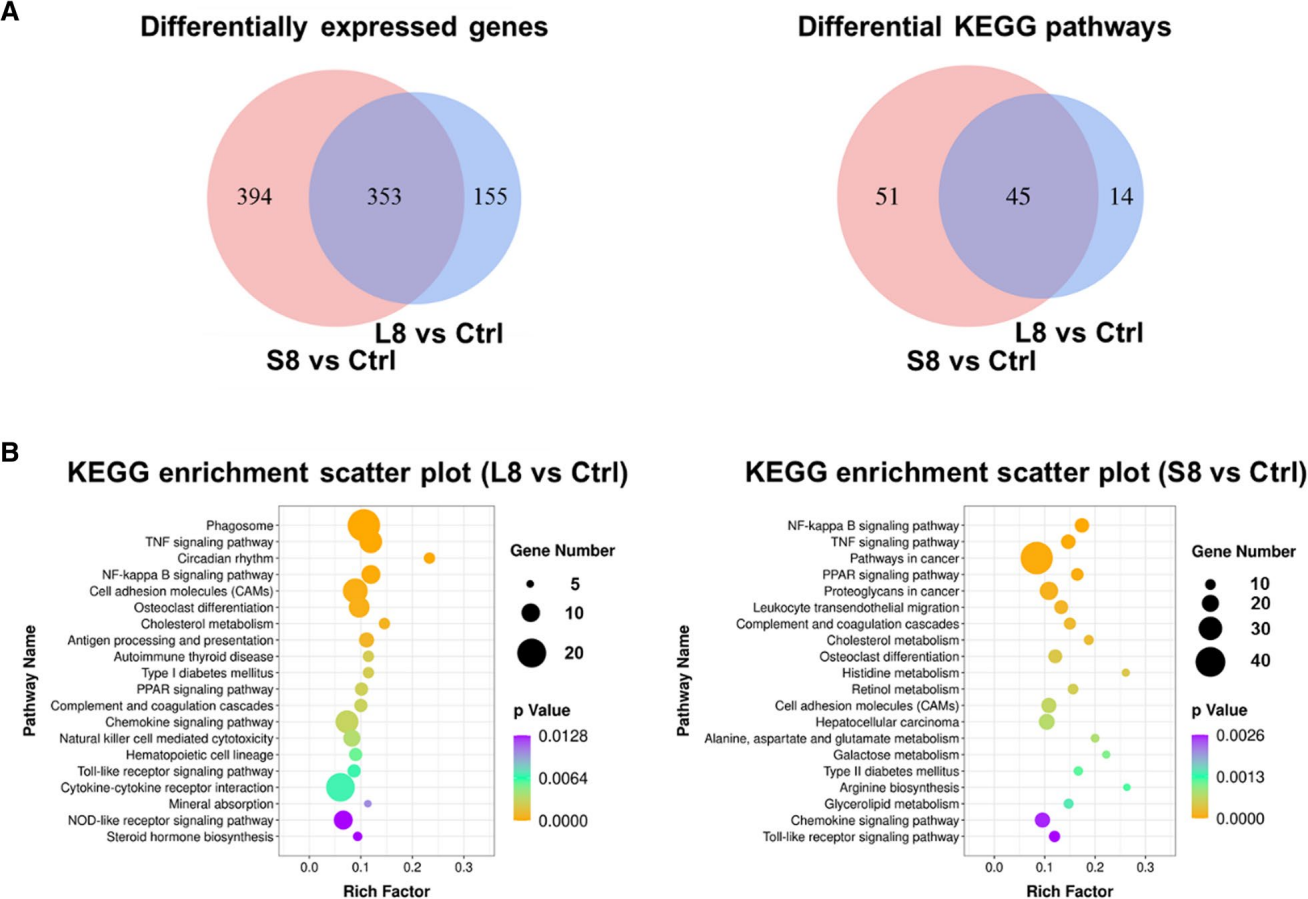
The differentially expressed genes (DEGs) and differential KEGG enrichment analysis between the control and LNPs/SNPs dosing groups (L8 and S8). **A** The Venn diagram showing the number of shared DEGs and differential KEGG pathways in the L8 and S8 groups compared to the control group. **B** Scatter plot of KEGG enrichment analysis of DEGs between the control and LNPs and SNPs dosing groups (L8 and S8). The top 20 KEGG pathway enrichments with DEGs are listed here. The rich factor (X-axis) indicates the ratio of DEGs enriched in a pathway to the total genes in that pathway. The diameter of the dots represents the absolute number of DEGs, and the color indicates the p value. L8 and S8: Rats were IV administered LNPs and SNPs, respectively, at a dose of 8 mg/kg daily for 14 consecutive days

To further illustrate the signaling pathways involved in the toxicity of BSA@Cu_2−x_S NPs and to compare the effects of sizes, differentially expressed genes (DEGs) were quantified by high-throughput RNA sequencing using the livers of the rats subjected to repeated dosing for 14 consecutive days. Pairwise comparison of the libraries between control (nontreated) and BSA@Cu_2−x_S NPs was performed after the calculation of gene expression abundance using the RSEM package. A total of 330 genes were upregulated and 178 genes were downregulated by LNPs at 8 mg/kg, while the number of upregulated or downregulated genes by SNPs was 464 and 283, respectively. Among these genes, 353 were shared by LNPs and SNPs, and SNPs showed unique features with more nonoverlapping genes and signaling pathways than LNPs (Fig. 7A). For more details on the expression levels of DEGs, please refer to Additional file 1: Table S3.

To further explore the signaling pathways changed by BSA@Cu_2−x_S NPs that were different in size, the Kyoto Encyclopedia of Genes and Genomes (KEGG) database was used to map and enrich these genes into bundles of pathways, and the top 20 KEGG pathways that significantly influenced LNPs and SNPs are listed (ranked by p-value). It seems that the inflammation modules (NF-kappa B signaling pathway and TNF signaling pathway, etc.) and lipid/drug metabolism-related pathways (such as the PPAR signaling pathway and cholesterol metabolism pathway, etc.) accounted for the major profile of BSA@Cu_2−x_S NPs-induced liver toxicity (Fig. 7B). It should also be noted that, unlike the SNPs, LNPs showed a significant alteration of phagosomal genes (total 18 genes), highlighting the unique behavior of active cellular uptake in the liver by LNPs that may be primarily due to the function of Kupffer cells.

### Reversibility of toxicity responses at the molecular level by qPCR

Based upon the results from the RNA sequencing and KEGG pathway analysis, we selected the representative genes in the abovementioned four pathways highly relevant to liver toxicity and metabolism to investigate whether these pathways/genes can be recovered to the basal level after 28 days of cessation of NPs administration. Additionally, since copper metabolism may be highly involved in the toxicity of BSA@Cu_2−x_S NPs, we also grouped the genes known for the binding and transportation of copper elements to investigate their reversibility.

**Fig. 8:**
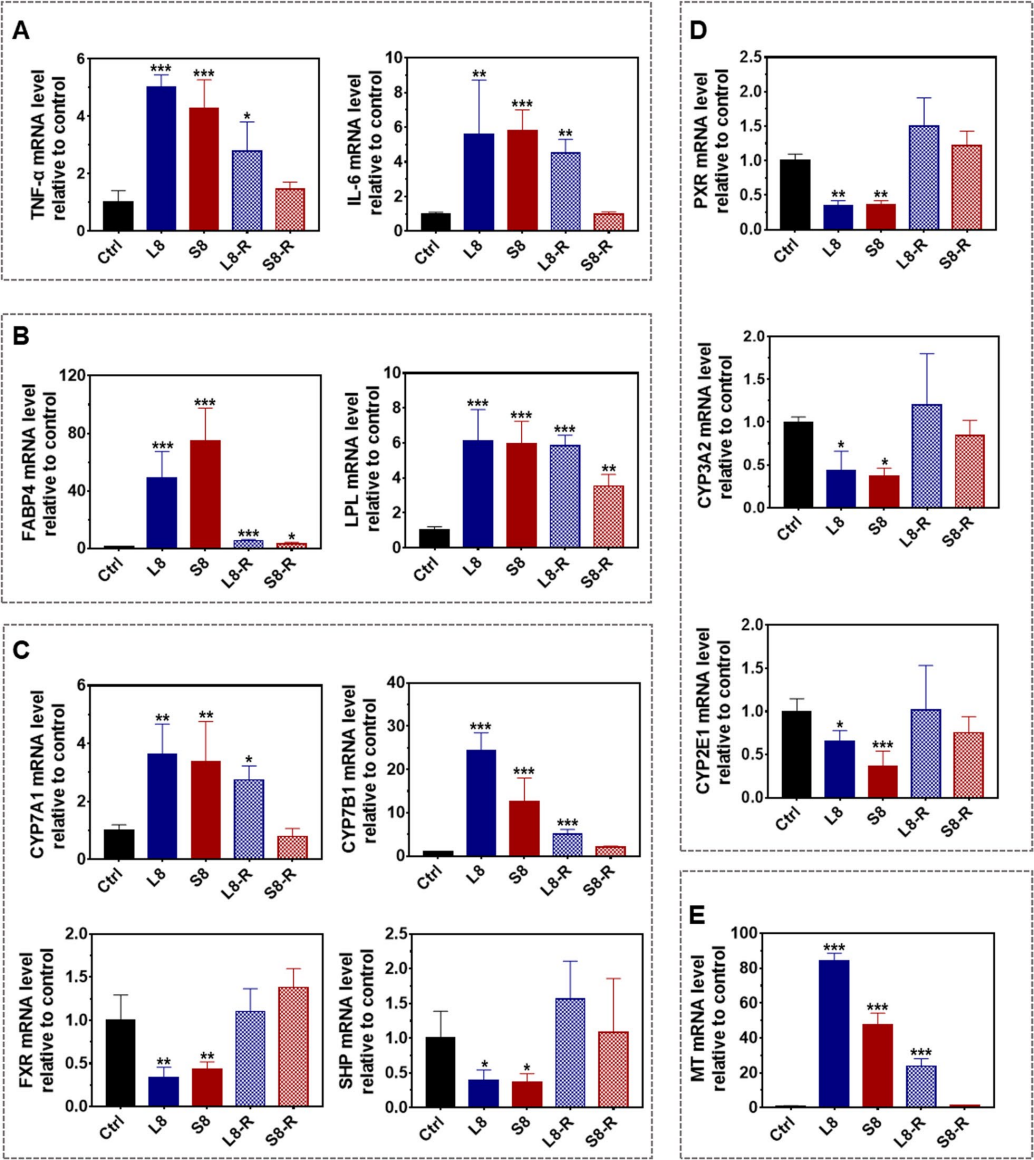
The mRNA expression of genes in the altered signaling pathways at the end of the dosing and recovery period. The rats were treated with 8 mg/kg LNPs and SNPs for 14 days and allowed to recover for 28 days, and the livers were collected for mRNA quantification by RT-qPCR. **A** Inflammatory pathways related genes: TNF-α and IL-6. **B** PPAR signaling pathway related genes: FABP4 and LPL. **C** Cholesterol and bile acid metabolism pathway-related genes: CYP7A1, CYP7B1, FXR and SHP. **D** Drug metabolism-cytochrome P450 pathway-related genes: PXR, CYP3A2 and CYP2E1. **E** Copper ion transport- and metabolism-related gene: metallothionein (MT). Values are represented as the mean ± SD. Statistical significance was assessed using one-way ANOVA (significance versus control: *p < 0.05, **p < 0.01, ***p < 0.001)

Inflammatory genes such as TNF-α and IL-6 in the rat livers were significantly upregulated by SNPs, while these two genes were fully recovered to the background level after the discontinuation of SNPs. However, these two inflammatory factors were continuously expressed after the recovery period of LNPs (L8-R), showing a comparable level to that of the treated samples (L8) (Fig. 8A). We also investigated the RNA level of IL-1β, suggesting the primary response of inflammation, but did not find an increase in this gene by either LNPs or SNPs (Additional file 1: Fig. S6A). Interestingly, IL-1β was significantly increased after the discontinuation of LNPs (L8-R), but it remained silent in the S8-R group, implying a delayed toxicity effect of LNPs mostly due to their accumulation in Kupffer cells. With a similar trend, the levels of FABP4 and LPL, two key genes in PPAR pathways related to cellular intermediary metabolism and inflammation, were highly expressed but slid back with a more attenuation effect by SNPs. Specifically, the FABP4 level was massively elevated (49- and 74-fold by LNPs and SNPs, respectively) but was reduced to a low level with SNPs samples closer to the background. The mRNA of LPL by LNPs remained unchanged after the recovery period, but LPL had an ~ 50% cut after a 28-day cessation of SNPs (Fig. 8B).

CYP7A1 (main pathway) and CYP7B1 (alternative pathway) are two enzymes that metabolize cholesterol into bile acids. FXR is a nuclear receptor that maintains bile acid homeostasis, and when bile acids increase, FXR is activated and negatively affects CYP7A1 through SHP to reduce the synthesis of bile acids [33]. As expected, our results revealed that BSA@Cu_2−x_S NPs had an inhibitory effect on FXR after 14 consecutive days of dosing and introduced SHP downregulation, which in turn upregulated CYP7A1/CYP7B1 (Fig. 8C). Again, SNPs showed much better reversibility of CYP7A1/CYP7B1 by approaching the background level, while their expression remained after the cessation of LNPs (Fig. 8C). Alternatively, drug metabolism-related enzymes, including PXR, CYP3A2 and CYP2E1, were decreased by both BSA@Cu_2−x_S NPs, with a similar trend of recovery after discontinuation of NPs (Fig. 8D). We also investigated the dosing and recovery profiles of the major transporter genes by BSA@Cu_2−x_S NPs. The gene expression of the bile acid efflux transporter proteins BSEP and MRP2 located in the apical membrane did not change significantly, and the level of NTCP located in the basolateral membrane did not change (Additional file 1: Fig. S6B). In comparison, the gene expression of bile acid efflux transporter proteins MRP3 and MRP4 located in the basolateral membrane was upregulated, which could be major contributors to the increased blood level of bile acid, as shown in Fig. 4A. These two proteins recovered to the basal level with no significant difference for either NPs.

Copper metabolism- and transportation-related genes were selected to explore their unique role in BSA@Cu_2−x_S NPs toxicity. Metallothioneins (MTs) are small cysteine-rich proteins for metal binding that play a critical role in metal transport, storage and detoxification [34, 35]. The MT expression level was markedly increased 84-fold and 47-fold by LNPs and SNPs, respectively, in the rat liver at the end of the 14-day dosing period (Fig. 8E). However, the MT level in the L8-R group remained at a high level after the recovery period, implying copper accumulation from LNPs in the liver. In contrast, MT expression completely returned to a background level after the discontinuation of SNPs, indicating that trace levels of copper had a minimal adverse effect on the rat liver after the recovery period. Other than MTs, BSA@Cu_2−x_S NPs of both sizes did not affect the expression of the ceruloplasmin (CP) and ATP7B genes, which suggests that excessive copper elements in the NPs did not activate copper transporters, and the mechanisms can be delineated in our future work (Additional file 1: Fig. S6C).

In conclusion, our qPCR analysis of five groups of genes indicated that LNPs and SNPs had differential adverse effects on inflammation, lipid metabolism, cholesterol and bile acid metabolism, drug metabolism, and metal homeostasis. More importantly, small-sized BSA@Cu_2−x_S NPs showed a much better recovery or reversible toxicity than large-sized NPs. The liver can be restored to a normal level concerning lipid/drug metabolism/transport, inflammation homeostasis and other typical functions after the discontinuation of SNPs, while the liver failed to recover after the cessation of LNPs, causing prolonged and delayed liver damage that may be likely due to the accumulated and imbalanced level of copper in Kupffer cells in the liver.

## Conclusion and perspectives

The enormous development of biomineralization methodologies enables NPs synthesis in aqueous solution. This method utilizes relatively biocompatible materials to obtain NPs without introducing any commonly used solvent with toxicity potential that needs to comply with ICH guidelines. Copper sulfide NPs with enhanced stability and photothermal conversion efficiency were obtained by our lab by sequentially mixing copper nitrate, BSA and sodium sulfide as the starting materials. This is a simplified process without introducing solvents and additives during synthesis, yielding nanoagents with minimal toxicity impurities. Although biomineralization-derived copper sulfide NPs have been recognized with strong translational potential over the past decade, safety evaluations to investigate the potential adverse effects of NPs have not been thoroughly performed. Here, we report the pharmacokinetics, distribution, metabolism and elimination of BSA@Cu_2−x_S NPs by a single-dose IV administration, delineating the hepatic accumulation of BSA@Cu_2−x_S NPs of two sizes based upon the threshold of glomerular filtration slit. The rapid clearance of small-sized NPs (SNPs) from the liver into the feces as the major elimination route was evident, while large-sized NPs (LNPs) were characterized by a slow elimination profile based upon PK and clearance data. To further promote the clinical translation of copper sulfide NPs by complying with ICH M3 regulatory guidance, we conducted a standard subacute (14-day consecutive administration) toxicity study followed by a 28-day recovery period. Our transcriptomic analysis and qPCR validation studies again highlighted the toxicity reversibility of the SNPs due to the complete recovery of pathway modules for inflammation, PPAR, drug/cholesterol metabolism and metal transport, while LNPs failed to recover from copper accumulation and toxicity. Cellular uptake studies in isolated hepatocytes and Kupffer cells from the rat liver illustrated the preferred storage of LNPs in Kupffer cells, which could be the primary attribute for the delayed and nonreversible toxicity uniquely induced by large-sized NPs.

In conclusion, our study proved that small-sized BSA@Cu_2−x_S NPs synthesized by a biomineralization process have advantages over large-sized equivalent due to their rapid clearance from the liver based on DMPK data and reversibility of liver functions. Although the risk of hepatotoxicity is documented in this work by the 14-day subacute study, it is reversible when SNPs are discontinued and should be manageable due to a single-dose administration in clinical practice. Overall, the DMPK and toxicity studies along with the transcriptomic analysis together provide solid evidence that ultrasmall copper sulfide NPs could be translated into clinical practice for photothermal therapy.

## Supplementary Information

**Additional file 1: Figure S1.** Physicochemical characterization of BSA@Cu_2−x_S NPs regarding to their photothermal and photoacoustic effects along with size and photothermal stability. **Figure S2.** The gross findings and organ coefficient analysis by LNPs and SNPs. **Figure S3.** Hematological parameters of the rats in control and dosing groups (2, 5 and 8 mg/kg for 14 days) administered with LNPs and SNPs at the end of dosing period. **Figure S4.** The serum CREA and UA levels as indicators for kidney injury in control rats and dosing groups at Day 1, 3, 7 and 14 when consecutively treated with LNPs and SNPs for 14 days. **Figure S5.** H&E staining of heart, liver, spleen, lung, and kidney for the rats in control and dosing groups administered with LNPs and SNPs. **Figure S6.** The mRNA level of IL-1β, PPAR-α, PPAR-γ, NTCP, BSEP, MRP2, MRP3, MRP4, ATP7B and Ceruloplasmin (CP) in the liver of the rats dosed with 8 mg/kg LNPs and SNPs after the dosing period and recovery period. **Table S1.** Primer pairs used for real-time quantitative PCR. **Table S2.** Pharmacokinetic parameters of LNPs and SNPs by a single-dose intravenous injection in the SD rats. **Table S3. **The differentially expressed genes (DEGs) between control and L- or S-BSA@Cu_2−x_S NPs dosing groups (L8 and S8).

## Data Availability

All data generated in this study are included in this publication.

## Abbreviations

BSA@Cu_2__−__x_S NPs: Bovine serum albumin-biomineralized copper sulfide nanoparticles
LNPs: L-BSA@Cu_2__−__x_S NPs
SNPs: S-BSA@Cu_2__−__x_S NPs
PTT: Photothermal therapy
NIR: Near infrared region
DMPK: Distribution, metabolism and pharmacokinetics
IND: Investigative new drug application
DEGs: Differentially expressed genes
BW: Body weight
TEM: Transmission electron microscope
XPS: X-ray photoelectron spectroscopy
UV–Vis–NIR: Ultraviolet–visible–near infrared
ALT: Alanine transaminase
AST: Aspartate transaminase
TBA: Total bile acid
ALB: Albumin
LDH: Lactate dehydrogenase
WBC: White blood cell
RBC: Red blood cell
HGB: Hemoglobin
HCT: Hematocrit
MCV: Mean cell volume
MCH: Mean cell hemoglobin
MCHC: Mean cell hemoglobin concentration
CHCM: Cellular hemoglobin concentration mean
CH: Cellular hemoglobin
RDW: Red cell distribution width
PLT: Platelet count
MPV: Mean platelet volume
t_1/2α_: Blood distribution half-life
t_1/2β_: Blood terminal elimination half-life
AUC: Area under the blood level-time curve
Vc: Volume of distribution in center compartment
CL: Total body clearance
MRT: Mean residence time
