# Supplementary material for: Preclinical safety and hepatotoxicity evaluation of biomineralized copper sulfide nanoagents
Source: J Nanobiotechnology. 2022 Apr 12;20:185. doi: 10.1186/s12951-022-01399-5 (PMC9004045; doi:10.1186/s12951-022-01399-5)
Supplement: Supplementary file 1 — Additional file 1: Figure S1. Physicochemical characterization of BSA@Cu2−xS NPs regarding to their photothermal and photoacoustic effects along with size and photothermal stability. Figure S2. The gross findings and organ coefficient analysis by LNPs and SNPs. Figure S3. Hematological parameters of the rats in control and dosing groups (2, 5 and 8 mg/kg for 14 days) administered with LNPs and SNPs at the end of dosing period. Figure S4. The serum CREA and UA levels as indicators for kidney injury in control rats and dosing groups at Day 1, 3, 7 and 14 when consecutively treated with LNPs and SNPs for 14 days. Figure S5. H&E staining of heart, liver, spleen, lung, and kidney for the rats in control and dosing groups administered with LNPs and SNPs. Figure S6. The mRNA level of IL-1β, PPAR-α, PPAR-γ, NTCP, BSEP, MRP2, MRP3, MRP4, ATP7B and Ceruloplasmin (CP) in the liver of the rats dosed with 8 mg/kg LNPs and SNPs after the dosing period and recovery period. Table S1. Primer pairs used for real-time quantitative PCR. Table S2. Pharmacokinetic parameters of LNPs and SNPs by a single-dose intravenous injection in the SD rats. Table S3. The differentially expressed genes (DEGs) between control and L- or S-BSA@Cu2−xS NPs dosing groups (L8 and S8). [file 12951_2022_1399_MOESM1_ESM.docx]

Supporting information


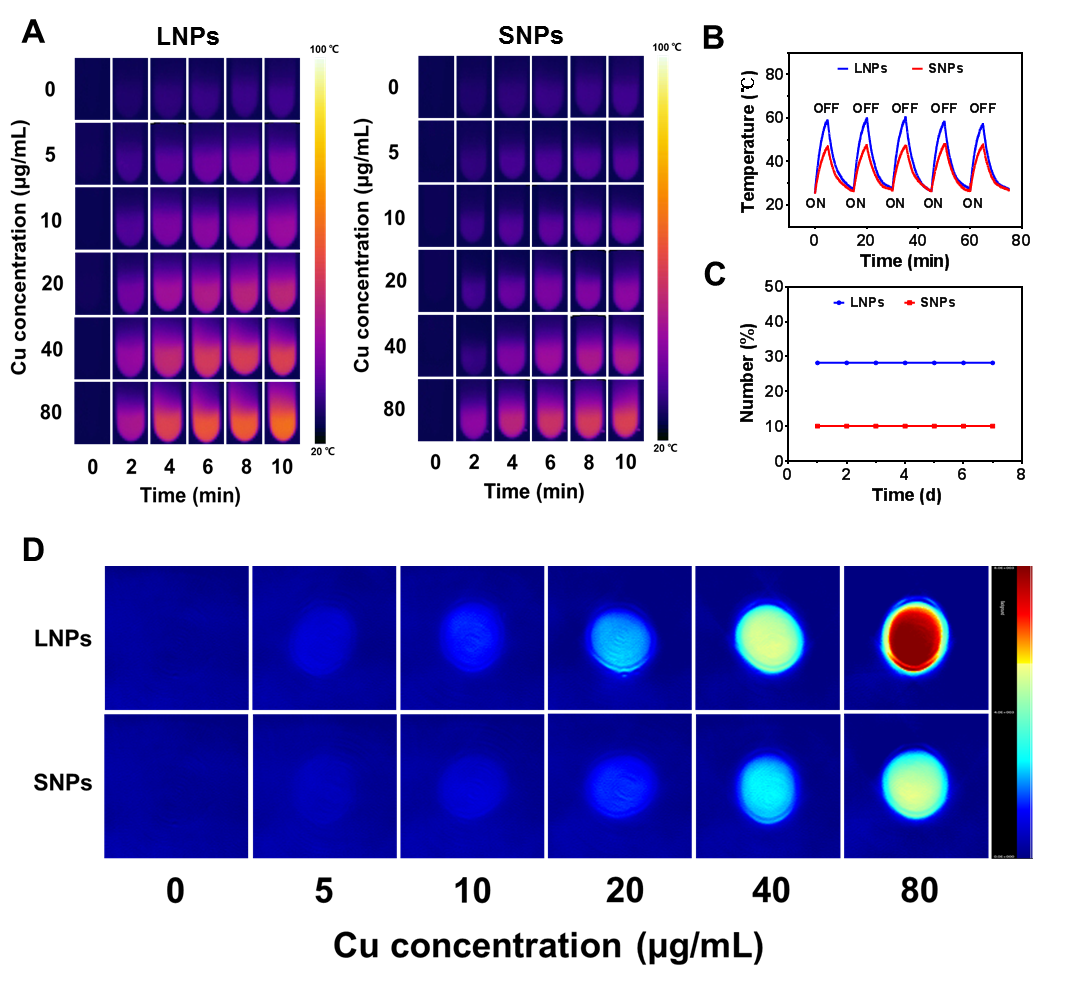


**Figure S1.** Physicochemical characterization of BSA@Cu_2-x_S NPs regarding to their photothermal and photoacoustic effects along with size and photothermal stability. (A) Real-time infrared thermal imaging of LNPs and SNPs (5, 10, 20, 40, 80 μg/mL of Cu) by NIR-II irradiation (1064 nm, 1 W/cm^2^ for 10 min). (B) Photothermal heating and natural cooling cycles of LNPs and SNPs (40 μg/mL of Cu) by laser irradiation. (C) Hydrodynamic sizes of LNPs and SNPs in deionized water for seven days. (D) Photoacoustic imaging of LNPs and SNPs in deionized water with different concentrations.


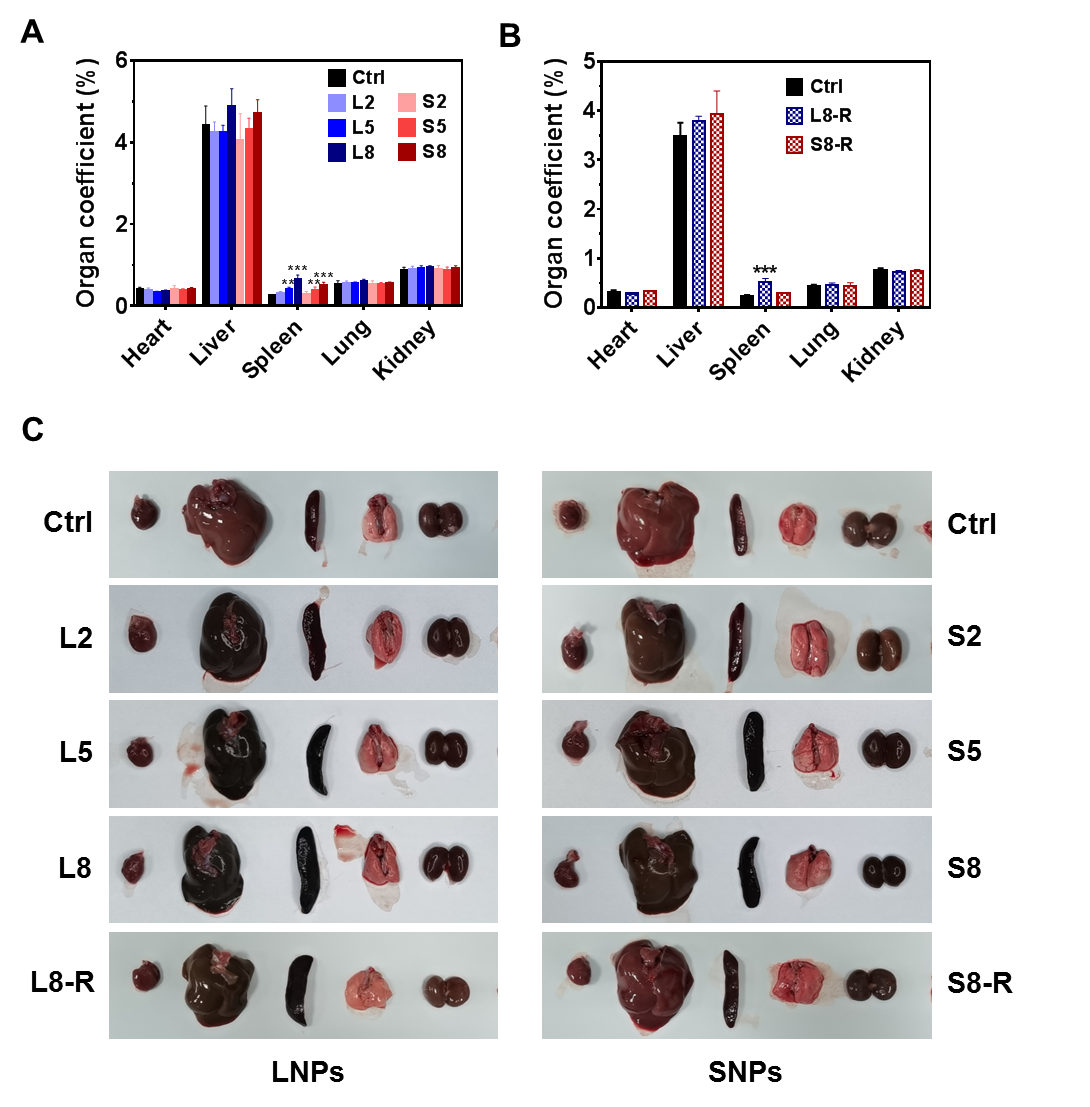


**Figure S2.** The gross findings and organ coefficient analysis by LNPs and SNPs. (A) The organ coefficients (The ratio of organ weight to body weight) of the rats in control and dosing groups at the end of 14 consecutive days of treatment. (B) The organ coefficients of the rats in control and recovery groups. (C) The gross finding of the rat organs in control, dosing groups and recovery groups. Notes: L2, L5, L8 and S2, S5, S8 stand for LNPs and SNPs at 2, 5, 8 mg/kg with IV administration for 14 consecutive days; L8-R and S8-R stand for rats subjected to LNPs and SNPs at 8 mg/kg daily for 14 days followed by a 28 days of recovery period. Values are represented as mean ± SD. Statistical significance was assessed using one-way ANOVA (significance versus control: **P* < 0.05, ***P* < 0.01, ****P* < 0.001).


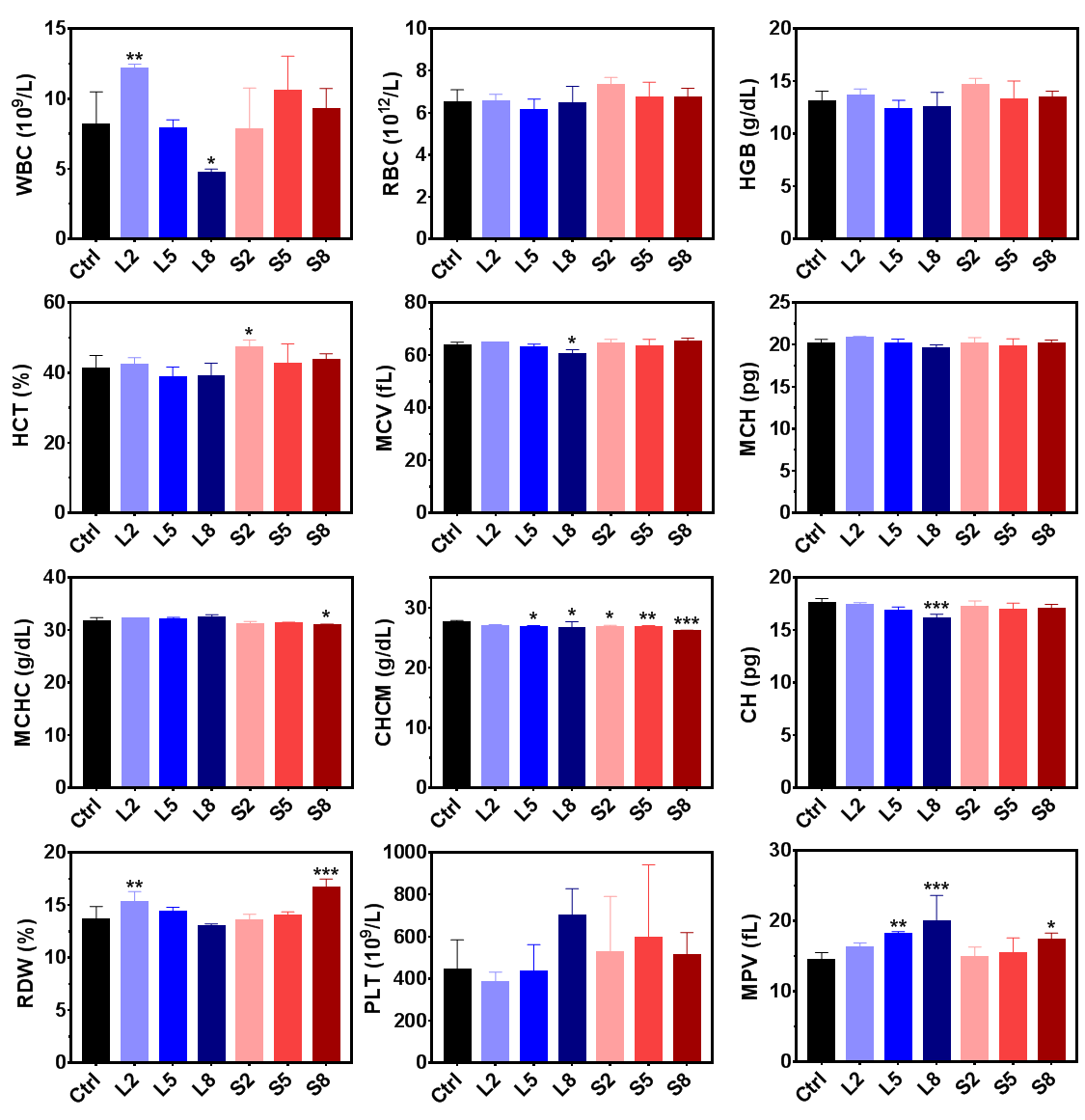


**Figure S3.** Hematological parameters of the rats in control and dosing groups (2, 5 and 8 mg/kg for 14 days) administered with LNPs and SNPs at the end of dosing period. Values are represented as mean ± SD. Statistical significance was assessed using one-way ANOVA (significance versus control: **P* < 0.05, ***P* < 0.01, ****P* < 0.001). WBC: white blood cell, RBC: red blood cell, HGB: hemoglobin, HCT: hematocrit, MCV: mean cell volume, MCH: mean cell hemoglobin, MCHC: mean cell hemoglobin concentration, CHCM: cellular hemoglobin concentration mean, CH: cellular hemoglobin, RDW: red cell distribution width, PLT: platelet count, MPV: mean platelet volume.


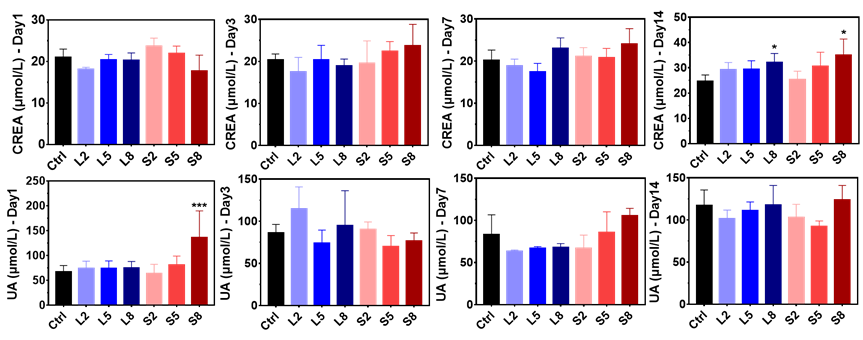


**Figure S4.** The serum CREA and UA levels as indicators for kidney injury in control rats and dosing groups at Day 1, 3, 7 and 14 when consecutively treated with LNPs and SNPs for 14 days. Values are represented as mean ± SD. Statistical significance was assessed using one-way ANOVA (significance versus control: **P* < 0.05, ****P* < 0.001). CREA: creatinine, UA: uric acid.


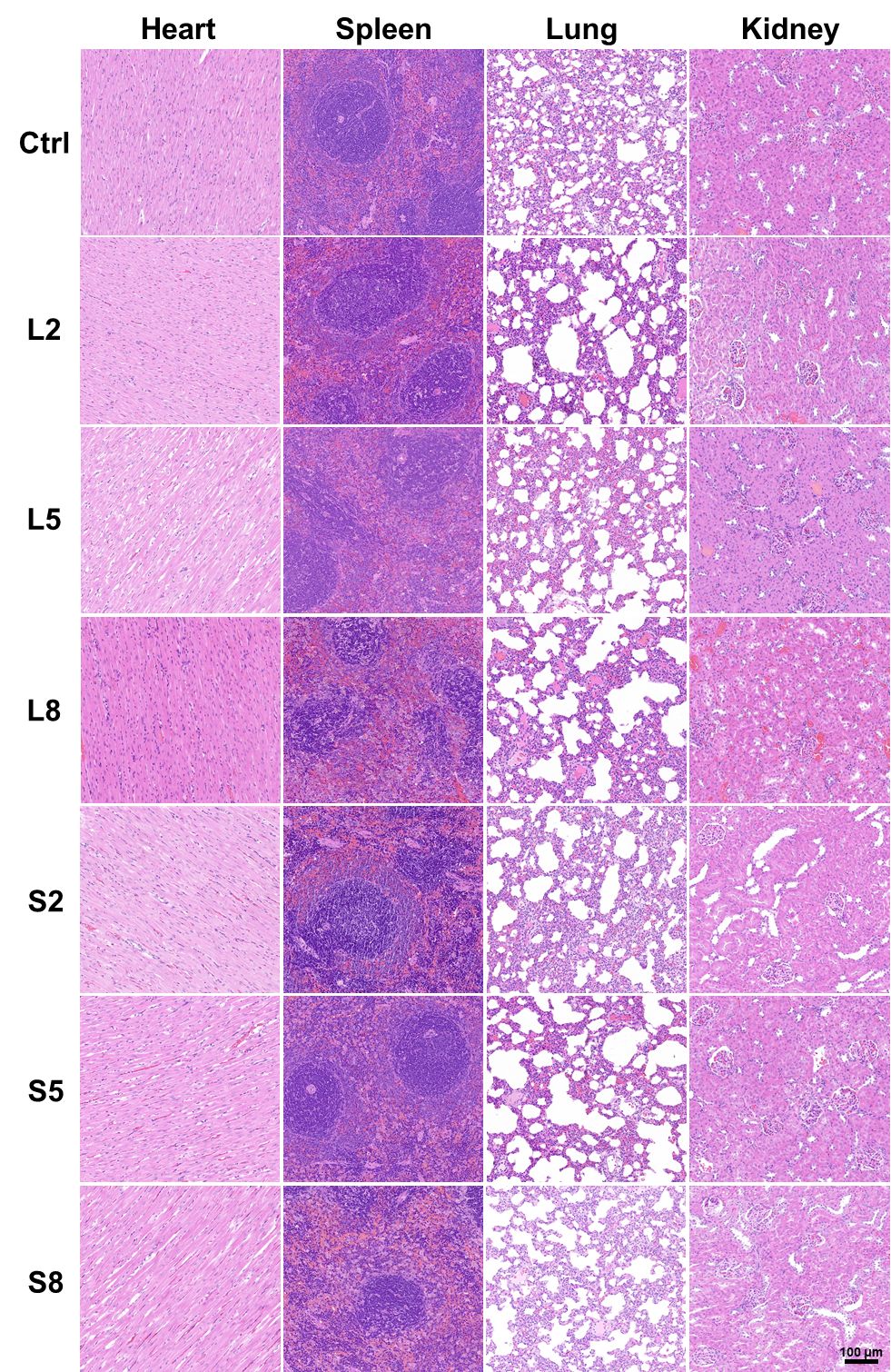


**Figure S5.** H&E staining of heart, liver, spleen, lung, and kidney for the rats in control and dosing groups administered with LNPs and SNPs. Rats were IV administered with LNPs and SNPs at 2, 5 and 8 mg/kg daily for 14 days and major organs other than livers were collected and stained for observation. The spleens of the rats in the mid (5 mg/kg) and high (8 mg/kg) doses of LNPs exhibited white marrow hyperplasia, thickening of the lymphatic sheath around the arteries, active lymph nodes (germinal center), extramedullary hematopoiesis, and trabecular hyperplasia. SNPs have less pathological changes in general. In comparison, the hearts, lungs, and kidneys had no distinctly pathological changes and structural abnormalities for both NPs.


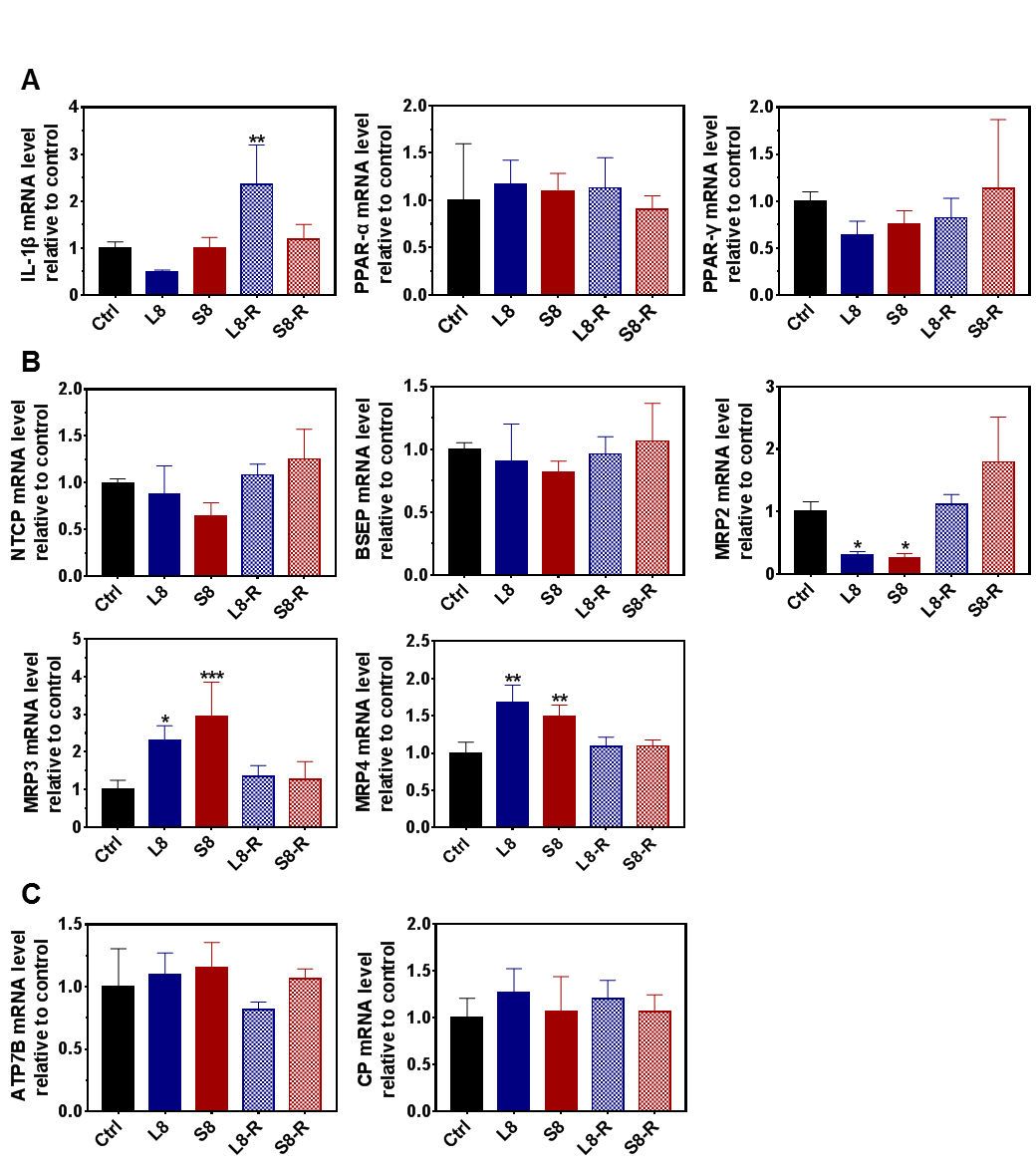


**Figure S6.** The mRNA level of IL-1β, PPAR-α, PPAR-γ, NTCP, BSEP, MRP2, MRP3, MRP4, ATP7B and Ceruloplasmin (CP) in the liver of the rats dosed with 8 mg/kg LNPs and SNPs after the dosing period and recovery period. Values are represented as mean ± SD. Statistical significance was assessed using one-way ANOVA (significance versus control: **P* < 0.05, ***P* < 0.01, ****P* < 0.001).

**Table S1.** Primer pairs used for real-time quantitative PCR.

| Primer | Forward | Reverse |
| --- | --- | --- |
| TNF-α | GCTCCCTCTCATCAGTTCC | CTCCTCTGCTTGGTGGTTTG |
| IL-6 | ACCACCCACAACAGACCAGT | ACAGTGCATCATTCGCTGTTC |
| IL-1β | TGACTCGTGGGATGATGACG | CTGGAGACTGCCCATTCTCG |
| FABP4 (Fabp4) | AGAAGTGGGAGTTGGCTTCG | ACTCTCTGACCGGATGACGA |
| LPL (Lpl) | AGTCCCAGCTTTGTCATCGA | GCCCGACTTCTTCAGAGACT |
| CYP7A1 (Cyp7a1) | TTTGGGGAATTGCCGTGTTG | CGGAATCAACCCGTTCTCCA |
| CYP7B1 (Cyp7b1) | GCGTGACGAAATTGACAGCTT | ATCCTCTTGCACTTCACGGA |
| FXR (Nr1h4) | CAAGTGACCTCCACGACCAA | AAGGAACATGGCCTCGACTG |
| SHP (Nr0b2) | TACGCATACCTGAAAGGCAC | GGACTTCACACAATGCCCAGT |
| PXR (Nr1i2) | ACTTCAAGGATTTCCGGTGACT | GTAGGTTGACACATCGGCCA |
| CYP3A2 (Cyp3a2) | CAAATCCACTGATAGCTGGT | TCAGAGGTATCTGTGTTTCCT |
| CYP2E1 (Cyp2e1) | GGGTGGACTTTGCTGGCCGA | TCGTGGAGCTGCCCTGGGTC |
| MT (Mt) | TAAGCTACAGTCTCTCGCGG | AGGTGCAGGAACCACCAGTA |
| NTCP (Slc10a1) | AAAGGGGTGATCGTTGCCTT | GGTCATCACGATGCTGAGGT |
| BSEP (Abcb11) | AAGCTGGCAAAGGGTTGTTG | GCATACGCCTCCCATAAGCA |
| MRP2 (Abcc2) | TGACAACCTGAGCATAGGGC | CCTGTGAGCGATGGTGATGA |
| MRP3 (Abcc3) | CTGAGATCCCAAGACCAGCG | AGTCAGGTTGGAGTCCCAGA |
| MRP4 (Abcc4) | CCTGACCGCACTCATCAAGT | ACAGGACAGGTTCCTGAGGT |
| GAPDH | GGCACAGTCAAGGCTGAGAATG | ATGGTGGTGAAGACGCCAGTA |
|  |  |  |

**Table S2.** Pharmacokinetic parameters of LNPs and SNPs by a single-dose intravenous injection in the SD rats.

|  | t_1/2α_ (h) | t_1/2β_ (h) | AUC (h*ug/mL) | Vc (mL) | CL (mL/h) | MRT (h) |
| --- | --- | --- | --- | --- | --- | --- |
| LNPs | 0.089 | 124.12 | 65.23 | 114.35 | 17.57 | 175.68 |
| SNPs | 0.23 | 83.85 | 83.3 | 19.63 | 13.46 | 93.34 |

Notes: t_1/2α_, blood distribution half-life; t_1/2β_, blood terminal elimination half-life; AUC, area under the blood level-time curve; Vc, volume of distribution in center compartment; CL, total body clearance; MRT, mean residence time.

**Table S3.** The differentially expressed genes (DEGs) between control and L- or S- BSA@Cu_2-x_S NPs dosing groups (L8 and S8).

| **L8 vs Control** | | | | |
| --- | --- | --- | --- | --- |
| **Gene_ID** | **Gene_name** | **FPKM.L8** | **FPKM.Control** | **Regulation** |
| ENSRNOG00000013973 | Lcn2 | 5724.71 | 11.48 | up |
| ENSRNOG00000007886 | Orm1 | 14854.02 | 350.21 | up |
| ENSRNOG00000002802 | Cxcl1 | 61.24 | 2.27 | up |
| ENSRNOG00000029668 | Wfdc21 | 1483.88 | 66.65 | up |
| ENSRNOG00000057125 | Ddr1 | 6.24 | 0.27 | up |
| ENSRNOG00000010805 | Fabp4 | 53.30 | 2.57 | up |
| ENSRNOG00000034290 | Ccl21 | 56.51 | 2.94 | up |
| ENSRNOG00000038960 | RGD1309362 | 67.69 | 3.64 | up |
| ENSRNOG00000013464 | Spink1 | 29.60 | 1.51 | up |
| ENSRNOG00000049893 | LOC100910934 | 47.05 | 2.67 | up |
| ENSRNOG00000050647 | Hspa1b | 53.32 | 3.10 | up |
| ENSRNOG00000008816 | Gpnmb | 89.42 | 5.39 | up |
| ENSRNOG00000048951 | LOC100364500 | 18.52 | 1.08 | up |
| ENSRNOG00000011081 | Serpina7 | 30.16 | 2.02 | up |
| ENSRNOG00000005825 | Lyz2 | 228.99 | 15.84 | up |
| ENSRNOG00000019050 | Ifit1 | 43.20 | 3.07 | up |
| ENSRNOG00000014532 | Lbp | 428.56 | 33.41 | up |
| ENSRNOG00000010262 | Hdc | 10.70 | 0.80 | up |
| ENSRNOG00000011483 | S100a9 | 25.01 | 1.87 | up |
| ENSRNOG00000046950 | LOC100912481 | 18.01 | 1.43 | up |
| ENSRNOG00000060518 | AABR07015057 | 403.31 | 34.52 | up |
| ENSRNOG00000009488 | Cyp7a1 | 42.58 | 36.89 | up |
| ENSRNOG00000009730 | Cyp7b1 | 34.90 | 3.05 | up |
| ENSRNOG00000016957 | Igfbp2 | 12.20 | 1.02 | up |
| ENSRNOG00000009131 | Zc3h12a | 7.91 | 0.67 | up |
| ENSRNOG00000023397 | LOC100911104 | 15.73 | 1.33 | up |
| ENSRNOG00000058589 | AABR07046778 | 347.62 | 32.74 | up |
| ENSRNOG00000047276 | AABR07044711 | 9.16 | 0.83 | up |
| ENSRNOG00000009734 | Akr1b8 | 5.16 | 0.47 | up |
| ENSRNOG00000061438 | LOC100360491 | 19.68 | 1.95 | up |
| ENSRNOG00000001187 | Oasl | 10.31 | 1.06 | up |
| ENSRNOG00000039249 | AABR07035539 | 51.24 | 5.27 | up |
| ENSRNOG00000013250 | Pdcd5 | 29.79 | 3.07 | up |
| ENSRNOG00000033776 | LOC100359668 | 37.32 | 4.08 | up |
| ENSRNOG00000057153 | Pla1a | 20.79 | 2.38 | up |
| ENSRNOG00000036703 | Itgax | 2.83 | 0.31 | up |
| ENSRNOG00000054495 | Cldn2 | 8.70 | 1.01 | up |
| ENSRNOG00000055067 | 5S_rRNA | 175.42 | 20.36 | up |
| ENSRNOG00000021802 | Isg15 | 22.77 | 2.86 | up |
| ENSRNOG00000050183 | RT1-CE1 | 3.96 | 0.47 | up |
| ENSRNOG00000033235 | Relb | 4.38 | 0.52 | up |
| ENSRNOG00000010645 | Lgals3 | 65.94 | 8.46 | up |
| ENSRNOG00000032240 | Gbp5 | 7.30 | 0.92 | up |
| ENSRNOG00000018076 | Fmo5 | 70.40 | 9.22 | up |
| ENSRNOG00000014461 | Galns | 1.74 | 0.23 | up |
| ENSRNOG00000006094 | Cd44 | 4.06 | 0.54 | up |
| ENSRNOG00000002667 | Lamc2 | 2.36 | 0.31 | up |
| ENSRNOG00000033220 | Oas1f | 3.59 | 0.51 | up |
| ENSRNOG00000001963 | Mx2 | 39.73 | 6.00 | up |
| ENSRNOG00000033625 | AABR07027015 | 33.21 | 4.82 | up |
| ENSRNOG00000016581 | Serpinb1a | 7.68 | 1.11 | up |
| ENSRNOG00000043098 | Mt2A | 1543.06 | 239.54 | up |
| ENSRNOG00000014117 | Hmox1 | 98.66 | 15.80 | up |
| ENSRNOG00000050210 | RT1-CE10 | 29.45 | 4.69 | up |
| ENSRNOG00000022256 | Cxcl10 | 14.19 | 2.25 | up |
| ENSRNOG00000024689 | Hopx | 6.48 | 1.00 | up |
| ENSRNOG00000002946 | Socs3 | 11.63 | 1.87 | up |
| ENSRNOG00000017445 | Tubb2b | 6.62 | 1.07 | up |
| ENSRNOG00000001480 | Ncf1 | 8.52 | 1.42 | up |
| ENSRNOG00000021062 | Fxyd5 | 12.18 | 2.01 | up |
| ENSRNOG00000028016 | AABR07021402 | 3.28 | 0.52 | up |
| ENSRNOG00000038047 | Mt1 | 537.65 | 93.46 | up |
| ENSRNOG00000015674 | Acap1 | 3.12 | 0.52 | up |
| ENSRNOG00000013668 | Capg | 17.45 | 3.00 | up |
| ENSRNOG00000054515 | Fgd6 | 1.32 | 0.22 | up |
| ENSRNOG00000028814 | Oasl2 | 9.60 | 1.66 | up |
| ENSRNOG00000015366 | Neurl3 | 5.23 | 0.89 | up |
| ENSRNOG00000020560 | AABR07005821 | 4.79 | 0.84 | up |
| ENSRNOG00000056617 | Zswim8 | 3.86 | 0.71 | up |
| ENSRNOG00000032917 | Zfand2a | 14.84 | 2.81 | up |
| ENSRNOG00000022839 | Ifit3 | 9.97 | 1.87 | up |
| ENSRNOG00000002175 | Clock | 3.30 | 0.62 | up |
| ENSRNOG00000037198 | Usp18 | 26.70 | 5.11 | up |
| ENSRNOG00000015948 | Slc1a5 | 2.54 | 0.47 | up |
| ENSRNOG00000002776 | Sell | 3.34 | 0.60 | up |
| ENSRNOG00000009329 | Nr1d1 | 14.73 | 2.85 | up |
| ENSRNOG00000019728 | Itgam | 1.59 | 0.30 | up |
| ENSRNOG00000017414 | Irf7 | 45.85 | 8.97 | up |
| ENSRNOG00000059140 | Myo1g | 3.66 | 0.70 | up |
| ENSRNOG00000012702 | Nfya | 4.27 | 0.82 | up |
| ENSRNOG00000020136 | Tgm1 | 2.77 | 0.52 | up |
| ENSRNOG00000011984 | Cxcl14 | 4.85 | 0.91 | up |
| ENSRNOG00000011459 | Rhbdf2 | 7.41 | 1.45 | up |
| ENSRNOG00000047076 | Oas1g | 4.74 | 0.90 | up |
| ENSRNOG00000030387 | Kng1 | 1334.24 | 269.81 | up |
| ENSRNOG00000047606 | Bcl2a1 | 17.14 | 3.39 | up |
| ENSRNOG00000004554 | Dcn | 15.96 | 3.20 | up |
| ENSRNOG00000011329 | Pkm | 25.37 | 5.15 | up |
| ENSRNOG00000039390 | Slc37a2 | 1.62 | 0.32 | up |
| ENSRNOG00000049517 | Tnfaip3 | 6.20 | 1.26 | up |
| ENSRNOG00000025001 | Pcolce | 16.99 | 3.50 | up |
| ENSRNOG00000012630 | Rhoc | 11.90 | 2.48 | up |
| ENSRNOG00000051404 | AABR07014242 | 11.54 | 2.29 | up |
| ENSRNOG00000003954 | Il2rg | 3.37 | 0.69 | up |
| ENSRNOG00000013895 | Npdc1 | 14.42 | 3.03 | up |
| ENSRNOG00000054765 | Renbp | 5.02 | 1.03 | up |
| ENSRNOG00000017878 | Aldh1a7 | 7.03 | 1.48 | up |
| ENSRNOG00000059406 | Ier5 | 13.99 | 3.02 | up |
| ENSRNOG00000019430 | Coro1a | 26.38 | 5.79 | up |
| ENSRNOG00000020300 | Lsp1 | 18.21 | 4.00 | up |
| ENSRNOG00000002730 | Rgs5 | 12.67 | 2.73 | up |
| ENSRNOG00000012294 | Heph | 1.07 | 0.22 | up |
| ENSRNOG00000004040 | Abca8 | 5.43 | 1.21 | up |
| ENSRNOG00000031211 | Acsm5 | 17.23 | 3.91 | up |
| ENSRNOG00000018669 | Jak3 | 6.08 | 1.38 | up |
| ENSRNOG00000013463 | Kcnj8 | 8.88 | 2.03 | up |
| ENSRNOG00000017496 | Cnp | 19.49 | 4.52 | up |
| ENSRNOG00000052444 | Samd9 | 2.72 | 0.62 | up |
| ENSRNOG00000034038 | AABR07005844 | 5.20 | 1.19 | up |
| ENSRNOG00000004226 | Irak3 | 9.58 | 2.24 | up |
| ENSRNOG00000003866 | Cxcr4 | 4.19 | 0.97 | up |
| ENSRNOG00000058866 | Myo5a | 0.75 | 0.17 | up |
| ENSRNOG00000026702 | Jaml | 1.89 | 0.44 | up |
| ENSRNOG00000033192 | Osmr | 3.85 | 0.91 | up |
| ENSRNOG00000015644 | Ugcg | 7.16 | 1.74 | up |
| ENSRNOG00000060329 | Emb | 4.27 | 0.98 | up |
| ENSRNOG00000050251 | MGC105649 | 3.89 | 0.90 | up |
| ENSRNOG00000012109 | Otulinl | 4.61 | 1.10 | up |
| ENSRNOG00000011821 | S100a4 | 13.22 | 3.19 | up |
| ENSRNOG00000046601 | Unc5cl | 4.15 | 1.04 | up |
| ENSRNOG00000009822 | Tlr2 | 2.74 | 0.67 | up |
| ENSRNOG00000010208 | Timp1 | 12.02 | 2.98 | up |
| ENSRNOG00000000187 | Csf2rb | 6.17 | 1.58 | up |
| ENSRNOG00000010319 | Lcp1 | 20.16 | 5.22 | up |
| ENSRNOG00000050000 | AABR07034739 | 14.08 | 3.41 | up |
| ENSRNOG00000005731 | Birc3 | 7.00 | 1.79 | up |
| ENSRNOG00000011913 | Cp | 1642.51 | 430.00 | up |
| ENSRNOG00000005341 | Upp2 | 4.51 | 1.15 | up |
| ENSRNOG00000059857 | Rnd1 | 4.60 | 1.16 | up |
| ENSRNOG00000020465 | Ripk3 | 5.40 | 1.39 | up |
| ENSRNOG00000010362 | Anxa2 | 36.27 | 9.46 | up |
| ENSRNOG00000001959 | Mx1 | 5.66 | 1.47 | up |
| ENSRNOG00000023969 | Herc6 | 6.22 | 1.62 | up |
| ENSRNOG00000007650 | Cd63 | 132.71 | 34.93 | up |
| ENSRNOG00000016420 | Serpinb6b | 4.09 | 1.01 | up |
| ENSRNOG00000008144 | Irf1 | 18.92 | 4.99 | up |
| ENSRNOG00000043044 | Cnn2 | 10.44 | 2.67 | up |
| ENSRNOG00000019365 | Ablim3 | 4.19 | 1.08 | up |
| ENSRNOG00000005479 | Slc1a2 | 13.34 | 3.47 | up |
| ENSRNOG00000001431 | Rasa4 | 2.62 | 0.67 | up |
| ENSRNOG00000038955 | MGC105567 | 3.19 | 0.82 | up |
| ENSRNOG00000018257 | Hpx | 17297.52 | 4643.73 | up |
| ENSRNOG00000021412 | Slfn13 | 5.68 | 1.52 | up |
| ENSRNOG00000001270 | Hvcn1 | 3.23 | 0.82 | up |
| ENSRNOG00000040108 | RGD1565355 | 4.12 | 1.13 | up |
| ENSRNOG00000023226 | S100a10 | 31.36 | 8.55 | up |
| ENSRNOG00000008425 | Nav1 | 0.79 | 0.21 | up |
| ENSRNOG00000016117 | Myof | 0.90 | 0.24 | up |
| ENSRNOG00000014964 | Hp | 21863.58 | 6121.94 | up |
| ENSRNOG00000004192 | Arhgap30 | 3.53 | 0.97 | up |
| ENSRNOG00000013102 | Entpd2 | 2.90 | 0.78 | up |
| ENSRNOG00000015941 | Fkbp10 | 1.65 | 0.44 | up |
| ENSRNOG00000005811 | LOC688655 | 60.99 | 17.26 | up |
| ENSRNOG00000019737 | Sema4a | 4.73 | 1.33 | up |
| ENSRNOG00000037371 | Xaf1 | 9.15 | 2.53 | up |
| ENSRNOG00000043451 | Spp1 | 5.03 | 1.40 | up |
| ENSRNOG00000030027 | Fbxw17 | 2.16 | 0.57 | up |
| ENSRNOG00000011078 | LOC100912604 | 11.48 | 3.23 | up |
| ENSRNOG00000020309 | Gfra3 | 6.38 | 1.81 | up |
| ENSRNOG00000001369 | Oas1a | 17.62 | 5.06 | up |
| ENSRNOG00000007690 | Cmpk2 | 2.22 | 0.62 | up |
| ENSRNOG00000009331 | Hck | 11.07 | 3.17 | up |
| ENSRNOG00000033051 | Slc22a15 | 2.36 | 0.67 | up |
| ENSRNOG00000031706 | RGD1563601 | 8.93 | 2.50 | up |
| ENSRNOG00000061403 | AABR07039446 | 1.60 | 0.45 | up |
| ENSRNOG00000004972 | Upp1 | 3.56 | 1.01 | up |
| ENSRNOG00000037113 | Slfn2 | 18.95 | 5.50 | up |
| ENSRNOG00000048914 | Traf1 | 1.99 | 0.56 | up |
| ENSRNOG00000018092 | Cd83 | 2.72 | 0.77 | up |
| ENSRNOG00000018824 | Slc7a5 | 3.07 | 0.89 | up |
| ENSRNOG00000055010 | Axin2 | 5.07 | 1.48 | up |
| ENSRNOG00000021104 | Emp3 | 11.25 | 3.23 | up |
| ENSRNOG00000018877 | Zfp629 | 1.60 | 0.46 | up |
| ENSRNOG00000018659 | Csf1 | 6.95 | 2.04 | up |
| ENSRNOG00000014476 | Evl | 4.87 | 1.40 | up |
| ENSRNOG00000013736 | C9 | 1677.31 | 499.72 | up |
| ENSRNOG00000019219 | Vamp1 | 2.85 | 0.82 | up |
| ENSRNOG00000037690 | Sertad3 | 3.66 | 1.05 | up |
| ENSRNOG00000025164 | Bhlha15 | 3.61 | 1.06 | up |
| ENSRNOG00000012640 | Dpp7 | 13.97 | 4.14 | up |
| ENSRNOG00000001252 | Chst12 | 2.30 | 0.64 | up |
| ENSRNOG00000059586 | AABR07015080 | 13.26 | 3.79 | up |
| ENSRNOG00000020845 | Tyrobp | 44.84 | 13.31 | up |
| ENSRNOG00000006108 | Gngt2 | 17.12 | 4.97 | up |
| ENSRNOG00000003538 | Adamts4 | 0.80 | 0.23 | up |
| ENSRNOG00000047045 | LOC108348111 | 14.06 | 4.16 | up |
| ENSRNOG00000007539 | Rsad2 | 2.32 | 0.68 | up |
| ENSRNOG00000008369 | Gimap4 | 4.20 | 1.21 | up |
| ENSRNOG00000055956 | AABR07015078 | 1358.51 | 410.65 | up |
| ENSRNOG00000010941 | Tifa | 3.54 | 1.04 | up |
| ENSRNOG00000004424 | RGD1563962 | 2.17 | 0.63 | up |
| ENSRNOG00000010219 | Ralgds | 3.89 | 1.16 | up |
| ENSRNOG00000037409 | Scimp | 3.03 | 0.85 | up |
| ENSRNOG00000017403 | Apobr | 1.34 | 0.39 | up |
| ENSRNOG00000010794 | Dennd3 | 1.01 | 0.29 | up |
| ENSRNOG00000020038 | Chpf | 2.38 | 0.70 | up |
| ENSRNOG00000024846 | Ier5l | 2.24 | 0.64 | up |
| ENSRNOG00000007300 | C1qtnf6 | 1.22 | 0.35 | up |
| ENSRNOG00000014948 | Osgin1 | 119.78 | 36.55 | up |
| ENSRNOG00000025810 | Grcc10 | 26.36 | 7.97 | up |
| ENSRNOG00000004821 | Sntb1 | 2.13 | 0.63 | up |
| ENSRNOG00000004111 | Soat1 | 2.78 | 0.81 | up |
| ENSRNOG00000004249 | Tlr7 | 0.99 | 0.29 | up |
| ENSRNOG00000047213 | Gnpda1 | 6.27 | 1.89 | up |
| ENSRNOG00000043182 | Septin6 | 1.23 | 0.36 | up |
| ENSRNOG00000042499 | LOC100364435 | 207.83 | 63.90 | up |
| ENSRNOG00000012543 | Mcm3 | 2.54 | 0.76 | up |
| ENSRNOG00000009471 | Epsti1 | 3.82 | 1.13 | up |
| ENSRNOG00000004048 | Lrrk2 | 0.54 | 0.16 | up |
| ENSRNOG00000005807 | Ptpn7 | 1.02 | 0.30 | up |
| ENSRNOG00000029938 | Pik3c2b | 0.62 | 0.18 | up |
| ENSRNOG00000046005 | Scd2 | 8.91 | 2.76 | up |
| ENSRNOG00000026235 | Hk3 | 4.42 | 1.36 | up |
| ENSRNOG00000021161 | Fermt3 | 12.76 | 3.98 | up |
| ENSRNOG00000011973 | Il7 | 4.24 | 1.26 | up |
| ENSRNOG00000009389 | Ripk2 | 5.15 | 1.60 | up |
| ENSRNOG00000013805 | Tnip2 | 4.97 | 1.53 | up |
| ENSRNOG00000017429 | Lat | 4.03 | 1.22 | up |
| ENSRNOG00000017512 | Aldh3b1 | 1.57 | 0.47 | up |
| ENSRNOG00000021102 | Scn1b | 8.21 | 2.56 | up |
| ENSRNOG00000048402 | Igh-6 | 20.04 | 6.35 | up |
| ENSRNOG00000029386 | RT1-N2 | 13.13 | 4.17 | up |
| ENSRNOG00000047911 | Miip | 3.28 | 1.01 | up |
| ENSRNOG00000008409 | Myo1f | 4.69 | 1.49 | up |
| ENSRNOG00000054764 | Flt3 | 1.06 | 0.32 | up |
| ENSRNOG00000002396 | Serpinb8 | 1.79 | 0.57 | up |
| ENSRNOG00000015670 | Stx7 | 6.66 | 2.14 | up |
| ENSRNOG00000033215 | RT1-Db1 | 16.20 | 5.25 | up |
| ENSRNOG00000038881 | Hcls1 | 13.99 | 4.52 | up |
| ENSRNOG00000012960 | Uap1l1 | 5.01 | 1.61 | up |
| ENSRNOG00000046050 | Dennd1c | 1.56 | 0.48 | up |
| ENSRNOG00000000787 | AABR07044364 | 3.67 | 1.11 | up |
| ENSRNOG00000019810 | Des | 4.84 | 1.55 | up |
| ENSRNOG00000055687 | AABR07035273 | 0.62 | 0.19 | up |
| ENSRNOG00000002470 | Ifi47 | 18.14 | 5.93 | up |
| ENSRNOG00000011406 | Ccl4 | 6.79 | 2.14 | up |
| ENSRNOG00000043416 | Bcl3 | 16.78 | 5.50 | up |
| ENSRNOG00000016756 | Ptgir | 0.97 | 0.30 | up |
| ENSRNOG00000050148 | AABR07002973 | 12.33 | 3.90 | up |
| ENSRNOG00000029682 | Clic1 | 21.71 | 7.11 | up |
| ENSRNOG00000060052 | Tdrd15 | 1.18 | 0.38 | up |
| ENSRNOG00000059984 | Dll1 | 2.73 | 0.89 | up |
| ENSRNOG00000000655 | Ptprc | 5.98 | 1.97 | up |
| ENSRNOG00000008706 | Tbx3 | 5.19 | 1.72 | up |
| ENSRNOG00000021750 | Id1 | 8.16 | 2.65 | up |
| ENSRNOG00000047746 | AABR07000398 | 467.81 | 156.68 | up |
| ENSRNOG00000019550 | Slc11a2 | 14.11 | 4.72 | up |
| ENSRNOG00000000699 | Selplg | 8.72 | 2.88 | up |
| ENSRNOG00000003217 | Lgals3bp | 37.67 | 12.68 | up |
| ENSRNOG00000016257 | Cotl1 | 25.63 | 8.61 | up |
| ENSRNOG00000033809 | Mlh1 | 4.13 | 1.38 | up |
| ENSRNOG00000014336 | Mcm5 | 2.38 | 0.78 | up |
| ENSRNOG00000015582 | Me2 | 3.09 | 1.03 | up |
| ENSRNOG00000018729 | Rad9a | 3.48 | 1.13 | up |
| ENSRNOG00000017874 | Cd53 | 14.00 | 4.73 | up |
| ENSRNOG00000015347 | Trim45 | 1.39 | 0.45 | up |
| ENSRNOG00000046600 | AABR07015066 | 807.05 | 275.24 | up |
| ENSRNOG00000016547 | Rgs19 | 3.33 | 1.10 | up |
| ENSRNOG00000048771 | RGD1559482 | 4.85 | 1.63 | up |
| ENSRNOG00000039848 | Ak6 | 14.15 | 4.83 | up |
| ENSRNOG00000048973 | NEWGENE_1306399 | 1.81 | 0.61 | up |
| ENSRNOG00000004322 | Sh3kbp1 | 1.29 | 0.43 | up |
| ENSRNOG00000025764 | AC128848 | 694.30 | 239.31 | up |
| ENSRNOG00000002217 | Plac8 | 32.76 | 11.22 | up |
| ENSRNOG00000013014 | Cyba | 32.54 | 11.17 | up |
| ENSRNOG00000051006 | Ufsp1 | 5.96 | 1.97 | up |
| ENSRNOG00000004873 | Prkch | 3.00 | 1.01 | up |
| ENSRNOG00000060896 | AABR07063424 | 424.75 | 147.05 | up |
| ENSRNOG00000048411 | Uhrf1 | 1.12 | 0.37 | up |
| ENSRNOG00000046707 | AABR07063425 | 369.25 | 127.17 | up |
| ENSRNOG00000012862 | Spsb4 | 1.22 | 0.40 | up |
| ENSRNOG00000008046 | Tmem30b | 1.55 | 0.52 | up |
| ENSRNOG00000024363 | Sertad1 | 6.84 | 2.33 | up |
| ENSRNOG00000050885 | LOC100910526 | 2.03 | 0.69 | up |
| ENSRNOG00000011647 | S100a6 | 17.94 | 6.15 | up |
| ENSRNOG00000012181 | Lpl | 6.00 | 2.09 | up |
| ENSRNOG00000000485 | Bak1 | 6.94 | 2.40 | up |
| ENSRNOG00000009369 | Tor4a | 1.75 | 0.59 | up |
| ENSRNOG00000050430 | Vav1 | 3.07 | 1.05 | up |
| ENSRNOG00000031406 | Hps3 | 9.04 | 3.15 | up |
| ENSRNOG00000062247 | AABR07031521 | 36.69 | 12.87 | up |
| ENSRNOG00000008658 | Mitf | 0.86 | 0.29 | up |
| ENSRNOG00000058081 | AABR07069733 | 1.32 | 0.45 | up |
| ENSRNOG00000021724 | Ptprcap | 4.62 | 1.57 | up |
| ENSRNOG00000017869 | Irf8 | 20.65 | 7.29 | up |
| ENSRNOG00000022533 | Micall2 | 0.95 | 0.31 | up |
| ENSRNOG00000019854 | Napsa | 7.02 | 2.47 | up |
| ENSRNOG00000047351 | AABR07015081 | 96.91 | 33.27 | up |
| ENSRNOG00000047657 | C4a | 112.59 | 40.21 | up |
| ENSRNOG00000049047 | LOC690468 | 18.15 | 6.20 | up |
| ENSRNOG00000030930 | Samsn1 | 2.96 | 1.02 | up |
| ENSRNOG00000025584 | Agap2 | 0.90 | 0.31 | up |
| ENSRNOG00000033747 | Sp110 | 12.05 | 4.28 | up |
| ENSRNOG00000021510 | Tbc1d10c | 1.70 | 0.57 | up |
| ENSRNOG00000046447 | LOC100911485 | 4.34 | 1.53 | up |
| ENSRNOG00000050158 | LOC100911692 | 1.99 | 0.68 | up |
| ENSRNOG00000017680 | Dennd2d | 1.92 | 0.66 | up |
| ENSRNOG00000019311 | Nfkb2 | 9.02 | 3.22 | up |
| ENSRNOG00000008481 | Reep1 | 0.89 | 0.30 | up |
| ENSRNOG00000046535 | Ppm1m | 1.36 | 0.46 | up |
| ENSRNOG00000010549 | Tspo | 16.64 | 5.93 | up |
| ENSRNOG00000021628 | Wdr89 | 2.68 | 0.93 | up |
| ENSRNOG00000000596 | Fyn | 4.01 | 1.43 | up |
| ENSRNOG00000047800 | C5ar1 | 3.50 | 1.23 | up |
| ENSRNOG00000013526 | Rassf4 | 5.00 | 1.78 | up |
| ENSRNOG00000002462 | B9d1 | 4.15 | 1.45 | up |
| ENSRNOG00000016378 | Map3k8 | 2.39 | 0.85 | up |
| ENSRNOG00000043059 | Aplf | 2.09 | 0.73 | up |
| ENSRNOG00000050156 | AABR07063425 | 174.68 | 62.07 | up |
| ENSRNOG00000032605 | Rpl32 | 17.78 | 6.36 | up |
| ENSRNOG00000019943 | Slc7a6 | 1.13 | 0.39 | up |
| ENSRNOG00000015668 | Ccl19 | 3.85 | 1.32 | up |
| ENSRNOG00000011015 | Hivep2 | 0.65 | 0.23 | up |
| ENSRNOG00000000451 | RT1-Ba | 43.08 | 15.72 | up |
| ENSRNOG00000019058 | Gstm3l | 11.87 | 4.26 | up |
| ENSRNOG00000007490 | Gabrr2 | 1.56 | 0.55 | up |
| ENSRNOG00000021355 | Ca6 | 2.93 | 1.02 | up |
| ENSRNOG00000013269 | Tnfsf10 | 9.79 | 3.58 | up |
| ENSRNOG00000003486 | Mnda | 7.40 | 2.68 | up |
| ENSRNOG00000000768 | Ubd | 76.01 | 28.02 | up |
| ENSRNOG00000031058 | Was | 3.84 | 1.38 | up |
| ENSRNOG00000000926 | Mrps17 | 12.99 | 4.74 | up |
| ENSRNOG00000030530 | Gzmm | 5.02 | 1.79 | up |
| ENSRNOG00000008736 | Slamf8 | 1.82 | 0.65 | up |
| ENSRNOG00000056265 | LOC103689920 | 4.86 | 1.79 | up |
| ENSRNOG00000018237 | Gstp1 | 43.20 | 15.91 | up |
| ENSRNOG00000030154 | Cyp4a2 | 114.89 | 42.61 | up |
| ENSRNOG00000011541 | Cygb | 7.69 | 2.82 | up |
| ENSRNOG00000025731 | Ptma | 35.05 | 12.93 | up |
| ENSRNOG00000014653 | Arl11 | 3.09 | 1.10 | up |
| ENSRNOG00000010799 | Noct | 0.65 | 11.03 | down |
| ENSRNOG00000014387 | Chac1 | 1.55 | 23.36 | down |
| ENSRNOG00000010253 | Cd163 | 0.60 | 7.14 | down |
| ENSRNOG00000013137 | Clec4f | 5.81 | 65.82 | down |
| ENSRNOG00000013552 | Scd | 51.69 | 524.16 | down |
| ENSRNOG00000019358 | Esr1 | 0.40 | 3.97 | down |
| ENSRNOG00000051171 | G6pc | 61.70 | 535.68 | down |
| ENSRNOG00000038132 | Vsig4 | 2.13 | 17.14 | down |
| ENSRNOG00000007607 | Nr4a1 | 2.07 | 14.61 | down |
| ENSRNOG00000020272 | 5330417C22Rik | 0.68 | 4.83 | down |
| ENSRNOG00000017063 | Fcna | 2.81 | 18.75 | down |
| ENSRNOG00000001388 | Sds | 13.38 | 82.38 | down |
| ENSRNOG00000014338 | Slc25a25 | 28.83 | 163.71 | down |
| ENSRNOG00000056135 | Tsc22d3 | 17.05 | 92.49 | down |
| ENSRNOG00000020799 | LOC103690054 | 2.68 | 14.21 | down |
| ENSRNOG00000049192 | Hmgn5 | 1.18 | 6.19 | down |
| ENSRNOG00000005420 | Abcg8 | 0.35 | 1.85 | down |
| ENSRNOG00000017693 | Slc2a5 | 0.66 | 3.45 | down |
| ENSRNOG00000047977 | Tcim | 5.62 | 28.02 | down |
| ENSRNOG00000003634 | Zfp354a | 5.33 | 26.12 | down |
| ENSRNOG00000001189 | Sik1 | 3.22 | 15.72 | down |
| ENSRNOG00000051081 | AABR07048439 | 208.74 | 991.75 | down |
| ENSRNOG00000058780 | Igfbp1 | 115.22 | 535.14 | down |
| ENSRNOG00000042620 | Marveld1 | 6.40 | 29.05 | down |
| ENSRNOG00000002345 | Rasgef1b | 3.47 | 15.90 | down |
| ENSRNOG00000057557 | Prlr | 0.36 | 1.71 | down |
| ENSRNOG00000015036 | Ccn2 | 1.11 | 5.07 | down |
| ENSRNOG00000033348 | Duox1 | 0.37 | 1.69 | down |
| ENSRNOG00000018005 | Duoxa1 | 0.75 | 3.46 | down |
| ENSRNOG00000020503 | Cbln3 | 0.83 | 3.82 | down |
| ENSRNOG00000008050 | Stac3 | 33.29 | 142.29 | down |
| ENSRNOG00000023465 | Depp1 | 18.91 | 80.01 | down |
| ENSRNOG00000014350 | Ccn1 | 5.04 | 21.36 | down |
| ENSRNOG00000000547 | Tspyl4 | 0.58 | 2.58 | down |
| ENSRNOG00000003244 | Ltc4s | 4.81 | 20.47 | down |
| ENSRNOG00000012274 | Ddi2 | 6.23 | 25.54 | down |
| ENSRNOG00000027808 | Lilra5 | 0.53 | 2.33 | down |
| ENSRNOG00000055909 | Apoa4 | 173.97 | 704.07 | down |
| ENSRNOG00000006859 | Insig1 | 98.23 | 386.13 | down |
| ENSRNOG00000007830 | Apold1 | 1.51 | 5.92 | down |
| ENSRNOG00000014456 | Coq10b | 10.57 | 40.70 | down |
| ENSRNOG00000014008 | Mfsd2a | 16.71 | 64.09 | down |
| ENSRNOG00000009867 | Tgfb3 | 0.59 | 2.36 | down |
| ENSRNOG00000054181 | LOC100361547 | 0.96 | 3.82 | down |
| ENSRNOG00000059061 | Uqcr10 | 21.79 | 82.79 | down |
| ENSRNOG00000021243 | Siglec1 | 0.42 | 1.63 | down |
| ENSRNOG00000051860 | Rnase4 | 14.59 | 54.59 | down |
| ENSRNOG00000011420 | Mtmr7 | 0.91 | 3.49 | down |
| ENSRNOG00000006305 | Slc38a2 | 15.72 | 57.87 | down |
| ENSRNOG00000020836 | Rorc | 7.44 | 27.02 | down |
| ENSRNOG00000020254 | Per2 | 0.95 | 3.42 | down |
| ENSRNOG00000051227 | AABR07048487 | 31.32 | 111.39 | down |
| ENSRNOG00000025648 | Dhrs7l1 | 89.93 | 313.88 | down |
| ENSRNOG00000054286 | Rrm2 | 1.74 | 6.20 | down |
| ENSRNOG00000016348 | Tat | 233.29 | 788.83 | down |
| ENSRNOG00000039278 | Mcart1 | 1.18 | 4.04 | down |
| ENSRNOG00000007964 | Tp53inp1 | 2.37 | 8.22 | down |
| ENSRNOG00000019318 | Syt3 | 0.31 | 1.11 | down |
| ENSRNOG00000036318 | AC119336 | 47.49 | 161.28 | down |
| ENSRNOG00000023257 | Adamts9 | 0.31 | 1.06 | down |
| ENSRNOG00000047520 | AABR07048475 | 12.21 | 41.48 | down |
| ENSRNOG00000030719 | Csmd1 | 0.18 | 0.62 | down |
| ENSRNOG00000004502 | Hal | 27.99 | 92.23 | down |
| ENSRNOG00000007410 | Dab1 | 0.56 | 1.89 | down |
| ENSRNOG00000023509 | Irs2 | 2.67 | 8.82 | down |
| ENSRNOG00000001113 | Mmd2 | 0.83 | 2.84 | down |
| ENSRNOG00000007370 | Rnf144a | 0.28 | 0.95 | down |
| ENSRNOG00000045743 | Etnppl | 8.69 | 28.20 | down |
| ENSRNOG00000004500 | Myc | 5.72 | 18.60 | down |
| ENSRNOG00000025558 | Palm2 | 0.36 | 1.25 | down |
| ENSRNOG00000027024 | Rgs16 | 3.20 | 10.39 | down |
| ENSRNOG00000031136 | Ntng1 | 0.26 | 0.91 | down |
| ENSRNOG00000014508 | Mgll | 6.18 | 19.76 | down |
| ENSRNOG00000005250 | Abcg5 | 1.58 | 5.05 | down |
| ENSRNOG00000048194 | LOC100912380 | 16.59 | 52.52 | down |
| ENSRNOG00000015904 | Wfdc1 | 1.28 | 4.21 | down |
| ENSRNOG00000002212 | Hsd17b13 | 43.28 | 134.52 | down |
| ENSRNOG00000050539 | Fbln5 | 0.90 | 2.88 | down |
| ENSRNOG00000011019 | Faahl | 11.35 | 34.87 | down |
| ENSRNOG00000018166 | Prkab2 | 0.25 | 0.81 | down |
| ENSRNOG00000007387 | Per1 | 3.56 | 10.80 | down |
| ENSRNOG00000023657 | Gprin3 | 1.08 | 3.34 | down |
| ENSRNOG00000001607 | Adamts1 | 2.97 | 8.80 | down |
| ENSRNOG00000049590 | RT1-M2 | 0.88 | 2.79 | down |
| ENSRNOG00000014426 | Lox | 0.79 | 2.37 | down |
| ENSRNOG00000021405 | Cyp2c7 | 238.69 | 702.24 | down |
| ENSRNOG00000057832 | Rnf125 | 11.14 | 32.75 | down |
| ENSRNOG00000009316 | Bmp10 | 0.92 | 2.87 | down |
| ENSRNOG00000020035 | Cyp17a1 | 1.41 | 4.24 | down |
| ENSRNOG00000059330 | AABR07004549 | 274.71 | 801.53 | down |
| ENSRNOG00000012404 | Thrsp | 146.20 | 425.13 | down |
| ENSRNOG00000015858 | Hyal1 | 5.48 | 16.05 | down |
| ENSRNOG00000005826 | RGD1562420 | 2.28 | 7.03 | down |
| ENSRNOG00000017899 | Akr7a3 | 21.71 | 62.87 | down |
| ENSRNOG00000003977 | Dusp1 | 75.87 | 217.89 | down |
| ENSRNOG00000020716 | Axl | 1.93 | 5.50 | down |
| ENSRNOG00000058424 | AABR07057150 | 3.75 | 11.05 | down |
| ENSRNOG00000042721 | Gimd1 | 3.23 | 9.26 | down |
| ENSRNOG00000004362 | Rps6ka5 | 0.21 | 0.62 | down |
| ENSRNOG00000022268 | Pnpla3 | 2.65 | 7.46 | down |
| ENSRNOG00000030776 | Sytl2 | 0.67 | 1.97 | down |
| ENSRNOG00000020704 | Tkfc | 35.95 | 98.68 | down |
| ENSRNOG00000046727 | Abcc2 | 34.72 | 95.12 | down |
| ENSRNOG00000005758 | Btbd11 | 0.54 | 1.52 | down |
| ENSRNOG00000045989 | Hba-a3 | 3.94 | 11.28 | down |
| ENSRNOG00000008450 | LOC100359539 | 0.85 | 2.38 | down |
| ENSRNOG00000061180 | AABR07026377 | 1.27 | 3.54 | down |
| ENSRNOG00000037188 | Mug1 | 563.50 | 1510.00 | down |
| ENSRNOG00000024620 | Mamdc2 | 0.50 | 1.43 | down |
| ENSRNOG00000036397 | AABR07027722 | 80.63 | 215.82 | down |
| ENSRNOG00000032374 | Paqr9 | 31.84 | 84.19 | down |
| ENSRNOG00000022837 | Slc25a40 | 0.60 | 1.69 | down |
| ENSRNOG00000004403 | Slc25a32 | 5.34 | 14.11 | down |
| ENSRNOG00000019716 | Ntf3 | 0.92 | 2.57 | down |
| ENSRNOG00000030285 | Epha3 | 0.21 | 0.59 | down |
| ENSRNOG00000047457 | AABR07071765 | 2.04 | 5.38 | down |
| ENSRNOG00000004327 | Ddc | 7.12 | 18.40 | down |
| ENSRNOG00000011200 | Bhmt | 617.09 | 1581.68 | down |
| ENSRNOG00000000561 | Pald1 | 7.55 | 19.39 | down |
| ENSRNOG00000061821 | AC109891 | 1.30 | 3.38 | down |
| ENSRNOG00000006622 | Cry1 | 1.90 | 4.88 | down |
| ENSRNOG00000017601 | Srd5a1 | 47.75 | 120.96 | down |
| ENSRNOG00000049303 | Marco | 9.89 | 25.15 | down |
| ENSRNOG00000028616 | Pck1 | 523.94 | 1316.65 | down |
| ENSRNOG00000053452 | LOC100361457 | 14.96 | 37.64 | down |
| ENSRNOG00000046468 | Ptgfr | 1.31 | 3.34 | down |
| ENSRNOG00000042785 | Sesn2 | 7.81 | 19.59 | down |
| ENSRNOG00000018322 | Picalm | 9.59 | 23.91 | down |
| ENSRNOG00000018373 | Tln2 | 0.30 | 0.79 | down |
| ENSRNOG00000012807 | C1qa | 29.99 | 74.49 | down |
| ENSRNOG00000020467 | Nrep | 35.03 | 87.13 | down |
| ENSRNOG00000004100 | Trib1 | 8.54 | 21.22 | down |
| ENSRNOG00000006663 | Usp2 | 2.59 | 6.45 | down |
| ENSRNOG00000046643 | Cyp3a9 | 4.87 | 12.03 | down |
| ENSRNOG00000049900 | Irf2bp2 | 16.56 | 40.58 | down |
| ENSRNOG00000002947 | Dpt | 72.66 | 177.20 | down |
| ENSRNOG00000002035 | Paqr3 | 4.02 | 9.88 | down |
| ENSRNOG00000052810 | Cyp2c11 | 544.96 | 1325.12 | down |
| ENSRNOG00000005077 | AABR07015719 | 2.89 | 7.42 | down |
| ENSRNOG00000014367 | Ephb6 | 1.10 | 2.71 | down |
| ENSRNOG00000027784 | Tsku | 79.08 | 190.69 | down |
| ENSRNOG00000016275 | Ttr | 4674.30 | 11266.72 | down |
| ENSRNOG00000058388 | Zfp36 | 48.14 | 116.08 | down |
| ENSRNOG00000061519 | Asap2 | 0.99 | 2.44 | down |
| ENSRNOG00000003300 | Btg2 | 18.08 | 43.55 | down |
| ENSRNOG00000045742 | Cyb5d1 | 1.56 | 3.93 | down |
| ENSRNOG00000045686 | Nfs1 | 15.54 | 37.23 | down |
| ENSRNOG00000029862 | Spc24 | 2.29 | 5.68 | down |
| ENSRNOG00000004921 | Nusap1 | 0.43 | 1.06 | down |
| ENSRNOG00000024201 | Fer1l5 | 0.72 | 1.73 | down |
| ENSRNOG00000019319 | Fchsd2 | 4.10 | 9.78 | down |
| ENSRNOG00000007483 | Ccnf | 0.87 | 2.11 | down |
| ENSRNOG00000011815 | Sgk1 | 11.15 | 26.43 | down |
| ENSRNOG00000004198 | Stxbp6 | 1.01 | 2.54 | down |
| ENSRNOG00000047613 | AABR07048463 | 114.33 | 270.05 | down |
| ENSRNOG00000016866 | Fhl2 | 1.31 | 3.24 | down |
| ENSRNOG00000051854 | Enpep | 9.29 | 21.79 | down |
| ENSRNOG00000016037 | Mafb | 15.59 | 36.57 | down |
| ENSRNOG00000001009 | Bri3 | 8.11 | 19.06 | down |
| ENSRNOG00000047986 | Sult2a1 | 71.02 | 165.50 | down |
| ENSRNOG00000024650 | Ckap2 | 0.94 | 2.26 | down |
| ENSRNOG00000040289 | Xkr9 | 3.65 | 8.52 | down |
| ENSRNOG00000051232 | Gbe1 | 37.51 | 86.61 | down |
| ENSRNOG00000025088 | Tmc3 | 1.33 | 3.12 | down |
| ENSRNOG00000002593 | Esrrg | 1.16 | 2.71 | down |
| ENSRNOG00000055451 | Gcnt4 | 0.73 | 1.77 | down |
| ENSRNOG00000016444 | AABR07067506 | 8.98 | 20.92 | down |
| ENSRNOG00000011690 | Rmdn3 | 4.13 | 9.50 | down |
| ENSRNOG00000009715 | Me1 | 7.89 | 17.97 | down |
| ENSRNOG00000016975 | Pxmp4 | 20.93 | 47.49 | down |
| ENSRNOG00000017914 | Cavin3 | 22.30 | 50.60 | down |
| ENSRNOG00000061910 | Igfbp3 | 18.97 | 42.95 | down |
| ENSRNOG00000059519 | Bbox1 | 14.76 | 33.45 | down |
| ENSRNOG00000007197 | Nr1h4 | 31.18 | 39.66 | down |
| ENSRNOG00000007229 | Nr0b2 | 14.21 | 27.81 | down |
| ENSRNOG00000002906 | Nr1i2 | 43.79 | 55.82 | down |
| ENSRNOG00000002948 | Abcc3 | 0.35 | 0.28 | down |
| ENSRNOG00000010064 | Abcc4 | 1.40 | 1.37 | down |

| **S8 vs Control** | | | | |
| --- | --- | --- | --- | --- |
| **Gene_ID** | **Gene_name** | **FPKM.S8** | **FPKM.Control** | **Regulation** |
| ENSRNOG00000013973 | Lcn2 | 6910.03 | 11.48 | up |
| ENSRNOG00000013464 | Spink1 | 115.98 | 1.51 | up |
| ENSRNOG00000002802 | Cxcl1 | 160.41 | 2.27 | up |
| ENSRNOG00000007886 | Orm1 | 13322.79 | 350.21 | up |
| ENSRNOG00000014532 | Lbp | 1148.31 | 33.41 | up |
| ENSRNOG00000029668 | Wfdc21 | 2119.87 | 66.65 | up |
| ENSRNOG00000050647 | Hspa1b | 81.70 | 3.10 | up |
| ENSRNOG00000009734 | Akr1b8 | 11.77 | 0.47 | up |
| ENSRNOG00000010805 | Fabp4 | 57.57 | 2.57 | up |
| ENSRNOG00000011483 | S100a9 | 42.57 | 1.87 | up |
| ENSRNOG00000012862 | Spsb4 | 8.78 | 0.40 | up |
| ENSRNOG00000058083 | Metazoa_SRP | 1247.80 | 61.91 | up |
| ENSRNOG00000008816 | Gpnmb | 103.63 | 5.39 | up |
| ENSRNOG00000009131 | Zc3h12a | 11.55 | 0.67 | up |
| ENSRNOG00000053717 | Metazoa_SRP | 63.31 | 3.94 | up |
| ENSRNOG00000011984 | Cxcl14 | 13.28 | 0.91 | up |
| ENSRNOG00000033776 | LOC100359668 | 57.87 | 4.08 | up |
| ENSRNOG00000021802 | Isg15 | 40.01 | 2.86 | up |
| ENSRNOG00000043098 | Mt2A | 3240.92 | 239.54 | up |
| ENSRNOG00000020136 | Tgm1 | 7.36 | 0.52 | up |
| ENSRNOG00000046950 | LOC100912481 | 18.75 | 1.43 | up |
| ENSRNOG00000023397 | LOC100911104 | 17.56 | 1.33 | up |
| ENSRNOG00000022256 | Cxcl10 | 27.73 | 2.25 | up |
| ENSRNOG00000001187 | Oasl | 12.79 | 1.06 | up |
| ENSRNOG00000014117 | Hmox1 | 184.93 | 15.80 | up |
| ENSRNOG00000058589 | AABR07046778 | 383.44 | 32.74 | up |
| ENSRNOG00000047276 | AABR07044711 | 9.93 | 0.83 | up |
| ENSRNOG00000059857 | Rnd1 | 13.66 | 1.16 | up |
| ENSRNOG00000054515 | Fgd6 | 2.57 | 0.22 | up |
| ENSRNOG00000002667 | Lamc2 | 3.57 | 0.31 | up |
| ENSRNOG00000033625 | AABR07027015 | 54.89 | 4.82 | up |
| ENSRNOG00000061096 | Rn7sl1 | 776.16 | 73.49 | up |
| ENSRNOG00000015366 | Neurl3 | 9.63 | 0.89 | up |
| ENSRNOG00000033235 | Relb | 5.70 | 0.52 | up |
| ENSRNOG00000057153 | Pla1a | 24.69 | 2.38 | up |
| ENSRNOG00000001963 | Mx2 | 60.17 | 6.00 | up |
| ENSRNOG00000043451 | Spp1 | 14.15 | 1.40 | up |
| ENSRNOG00000005825 | Lyz2 | 155.33 | 15.84 | up |
| ENSRNOG00000004554 | Dcn | 31.32 | 3.20 | up |
| ENSRNOG00000010645 | Lgals3 | 82.37 | 8.46 | up |
| ENSRNOG00000038960 | RGD1309362 | 35.22 | 3.64 | up |
| ENSRNOG00000009488 | Cyp7a1 | 65.45 | 36.89 | up |
| ENSRNOG00000009730 | Cyp7b1 | 29.35 | 3.05 | up |
| ENSRNOG00000019050 | Ifit1 | 28.97 | 3.07 | up |
| ENSRNOG00000033220 | Oas1f | 4.97 | 0.51 | up |
| ENSRNOG00000030387 | Kng1 | 2461.61 | 269.81 | up |
| ENSRNOG00000038047 | Mt1 | 851.87 | 93.46 | up |
| ENSRNOG00000020560 | AABR07005821 | 7.53 | 0.84 | up |
| ENSRNOG00000039249 | AABR07035539 | 47.04 | 5.27 | up |
| ENSRNOG00000049517 | Tnfaip3 | 11.11 | 1.26 | up |
| ENSRNOG00000048951 | LOC100364500 | 9.65 | 1.08 | up |
| ENSRNOG00000004226 | Irak3 | 18.46 | 2.24 | up |
| ENSRNOG00000015948 | Slc1a5 | 4.00 | 0.47 | up |
| ENSRNOG00000018076 | Fmo5 | 73.95 | 9.22 | up |
| ENSRNOG00000013463 | Kcnj8 | 16.49 | 2.03 | up |
| ENSRNOG00000017414 | Irf7 | 71.81 | 8.97 | up |
| ENSRNOG00000037198 | Usp18 | 40.92 | 5.11 | up |
| ENSRNOG00000006094 | Cd44 | 4.34 | 0.54 | up |
| ENSRNOG00000055067 | 5S_rRNA | 160.03 | 20.36 | up |
| ENSRNOG00000020038 | Chpf | 5.53 | 0.70 | up |
| ENSRNOG00000033192 | Osmr | 7.12 | 0.91 | up |
| ENSRNOG00000032240 | Gbp5 | 7.18 | 0.92 | up |
| ENSRNOG00000022839 | Ifit3 | 14.46 | 1.87 | up |
| ENSRNOG00000060518 | AABR07015057 | 259.63 | 34.52 | up |
| ENSRNOG00000028016 | AABR07021402 | 4.20 | 0.52 | up |
| ENSRNOG00000016581 | Serpinb1a | 8.53 | 1.11 | up |
| ENSRNOG00000019500 | Cyp1a1 | 4.31 | 0.58 | up |
| ENSRNOG00000036703 | Itgax | 2.30 | 0.31 | up |
| ENSRNOG00000019048 | Sod2 | 348.99 | 50.32 | up |
| ENSRNOG00000032917 | Zfand2a | 19.52 | 2.81 | up |
| ENSRNOG00000003954 | Il2rg | 4.96 | 0.69 | up |
| ENSRNOG00000028814 | Oasl2 | 11.54 | 1.66 | up |
| ENSRNOG00000034290 | Ccl21 | 20.60 | 2.94 | up |
| ENSRNOG00000025001 | Pcolce | 23.68 | 3.50 | up |
| ENSRNOG00000005479 | Slc1a2 | 23.70 | 3.47 | up |
| ENSRNOG00000002946 | Socs3 | 12.98 | 1.87 | up |
| ENSRNOG00000002542 | Heatr6 | 12.28 | 1.84 | up |
| ENSRNOG00000034038 | AABR07005844 | 8.00 | 1.19 | up |
| ENSRNOG00000028896 | A2m | 22.67 | 3.52 | up |
| ENSRNOG00000010262 | Hdc | 5.23 | 0.80 | up |
| ENSRNOG00000050000 | AABR07034739 | 22.73 | 3.41 | up |
| ENSRNOG00000010302 | LOC103694863 | 5.89 | 0.89 | up |
| ENSRNOG00000001959 | Mx1 | 9.19 | 1.47 | up |
| ENSRNOG00000045772 | LOC100911545 | 23.85 | 3.88 | up |
| ENSRNOG00000019365 | Ablim3 | 6.81 | 1.08 | up |
| ENSRNOG00000049893 | LOC100910934 | 16.69 | 2.67 | up |
| ENSRNOG00000024689 | Hopx | 6.50 | 1.00 | up |
| ENSRNOG00000021027 | Dbp | 9.68 | 1.56 | up |
| ENSRNOG00000003866 | Cxcr4 | 6.08 | 0.97 | up |
| ENSRNOG00000021062 | Fxyd5 | 12.61 | 2.01 | up |
| ENSRNOG00000050251 | MGC105649 | 5.80 | 0.90 | up |
| ENSRNOG00000034190 | Ighm | 14.25 | 2.37 | up |
| ENSRNOG00000043416 | Bcl3 | 33.12 | 5.50 | up |
| ENSRNOG00000039390 | Slc37a2 | 1.98 | 0.32 | up |
| ENSRNOG00000011459 | Rhbdf2 | 8.75 | 1.45 | up |
| ENSRNOG00000007539 | Rsad2 | 4.14 | 0.68 | up |
| ENSRNOG00000056617 | Zswim8 | 4.26 | 0.71 | up |
| ENSRNOG00000017878 | Aldh1a7 | 8.93 | 1.48 | up |
| ENSRNOG00000047076 | Oas1g | 5.44 | 0.90 | up |
| ENSRNOG00000015674 | Acap1 | 3.12 | 0.52 | up |
| ENSRNOG00000008706 | Tbx3 | 10.01 | 1.72 | up |
| ENSRNOG00000054765 | Renbp | 6.29 | 1.03 | up |
| ENSRNOG00000011081 | Serpina7 | 11.84 | 2.02 | up |
| ENSRNOG00000012640 | Dpp7 | 23.81 | 4.14 | up |
| ENSRNOG00000018669 | Jak3 | 7.88 | 1.38 | up |
| ENSRNOG00000009389 | Ripk2 | 9.18 | 1.60 | up |
| ENSRNOG00000029082 | AABR07005031 | 21.08 | 3.67 | up |
| ENSRNOG00000013668 | Capg | 16.82 | 3.00 | up |
| ENSRNOG00000013895 | Npdc1 | 17.03 | 3.03 | up |
| ENSRNOG00000002730 | Rgs5 | 15.70 | 2.73 | up |
| ENSRNOG00000011329 | Pkm | 28.62 | 5.15 | up |
| ENSRNOG00000007080 | Kif12 | 2.33 | 0.39 | up |
| ENSRNOG00000008369 | Gimap4 | 6.97 | 1.21 | up |
| ENSRNOG00000019728 | Itgam | 1.71 | 0.30 | up |
| ENSRNOG00000001369 | Oas1a | 27.71 | 5.06 | up |
| ENSRNOG00000014870 | Slc13a5 | 7.77 | 1.41 | up |
| ENSRNOG00000001480 | Ncf1 | 7.80 | 1.42 | up |
| ENSRNOG00000058866 | Myo5a | 0.99 | 0.17 | up |
| ENSRNOG00000047213 | Gnpda1 | 10.34 | 1.89 | up |
| ENSRNOG00000005731 | Birc3 | 9.74 | 1.79 | up |
| ENSRNOG00000051404 | AABR07014242 | 13.00 | 2.29 | up |
| ENSRNOG00000023969 | Herc6 | 8.67 | 1.62 | up |
| ENSRNOG00000008602 | Steap4 | 109.30 | 20.99 | up |
| ENSRNOG00000023226 | S100a10 | 45.21 | 8.55 | up |
| ENSRNOG00000010362 | Anxa2 | 49.10 | 9.46 | up |
| ENSRNOG00000053735 | Hebp2 | 5.77 | 1.06 | up |
| ENSRNOG00000017496 | Cnp | 23.38 | 4.52 | up |
| ENSRNOG00000059140 | Myo1g | 3.68 | 0.70 | up |
| ENSRNOG00000008425 | Nav1 | 1.12 | 0.21 | up |
| ENSRNOG00000059406 | Ier5 | 15.22 | 3.02 | up |
| ENSRNOG00000021412 | Slfn13 | 7.61 | 1.52 | up |
| ENSRNOG00000015644 | Ugcg | 8.67 | 1.74 | up |
| ENSRNOG00000016957 | Igfbp2 | 5.28 | 1.02 | up |
| ENSRNOG00000047365 | LOC100360647 | 100.69 | 20.39 | up |
| ENSRNOG00000060329 | Emb | 5.13 | 0.98 | up |
| ENSRNOG00000010208 | Timp1 | 15.12 | 2.98 | up |
| ENSRNOG00000018237 | Gstp1 | 78.25 | 15.91 | up |
| ENSRNOG00000037744 | Oas1i | 4.53 | 0.89 | up |
| ENSRNOG00000005807 | Ptpn7 | 1.56 | 0.30 | up |
| ENSRNOG00000052444 | Samd9 | 3.09 | 0.62 | up |
| ENSRNOG00000001270 | Hvcn1 | 4.26 | 0.82 | up |
| ENSRNOG00000009329 | Nr1d1 | 13.85 | 2.85 | up |
| ENSRNOG00000051952 | Tes | 6.37 | 1.30 | up |
| ENSRNOG00000033051 | Slc22a15 | 3.27 | 0.67 | up |
| ENSRNOG00000047045 | LOC108348111 | 20.15 | 4.16 | up |
| ENSRNOG00000043059 | Aplf | 3.59 | 0.73 | up |
| ENSRNOG00000013452 | Rcn1 | 25.17 | 5.23 | up |
| ENSRNOG00000012543 | Mcm3 | 3.73 | 0.76 | up |
| ENSRNOG00000050148 | AABR07002973 | 19.28 | 3.90 | up |
| ENSRNOG00000011541 | Cygb | 13.49 | 2.82 | up |
| ENSRNOG00000023546 | Hspb1 | 25.84 | 5.39 | up |
| ENSRNOG00000040108 | RGD1565355 | 5.33 | 1.13 | up |
| ENSRNOG00000012960 | Uap1l1 | 7.58 | 1.61 | up |
| ENSRNOG00000017114 | Synj2 | 1.61 | 0.33 | up |
| ENSRNOG00000003217 | Lgals3bp | 58.52 | 12.68 | up |
| ENSRNOG00000046005 | Scd2 | 12.72 | 2.76 | up |
| ENSRNOG00000018824 | Slc7a5 | 4.17 | 0.89 | up |
| ENSRNOG00000016962 | Neu2 | 2.98 | 0.62 | up |
| ENSRNOG00000049829 | AABR07060872 | 48.20 | 10.53 | up |
| ENSRNOG00000014948 | Osgin1 | 165.54 | 36.55 | up |
| ENSRNOG00000019822 | Gadd45b | 15.72 | 3.42 | up |
| ENSRNOG00000018659 | Csf1 | 9.27 | 2.04 | up |
| ENSRNOG00000049282 | Oas2 | 1.82 | 0.38 | up |
| ENSRNOG00000004048 | Lrrk2 | 0.75 | 0.16 | up |
| ENSRNOG00000011913 | Cp | 1913.76 | 430.00 | up |
| ENSRNOG00000020300 | Lsp1 | 18.01 | 4.00 | up |
| ENSRNOG00000012109 | Otulinl | 5.04 | 1.10 | up |
| ENSRNOG00000011774 | Fblim1 | 1.90 | 0.41 | up |
| ENSRNOG00000016420 | Serpinb6b | 4.75 | 1.01 | up |
| ENSRNOG00000015582 | Me2 | 4.60 | 1.03 | up |
| ENSRNOG00000019430 | Coro1a | 25.29 | 5.79 | up |
| ENSRNOG00000015845 | Niban2 | 8.99 | 2.05 | up |
| ENSRNOG00000007650 | Cd63 | 151.45 | 34.93 | up |
| ENSRNOG00000050869 | Cebpd | 31.30 | 7.20 | up |
| ENSRNOG00000007690 | Cmpk2 | 2.76 | 0.62 | up |
| ENSRNOG00000010941 | Tifa | 4.59 | 1.04 | up |
| ENSRNOG00000037380 | AC136661 | 16.47 | 3.73 | up |
| ENSRNOG00000021155 | Ctsk | 6.67 | 1.52 | up |
| ENSRNOG00000018651 | Agtpbp1 | 1.16 | 0.26 | up |
| ENSRNOG00000031475 | Col16a1 | 1.27 | 0.28 | up |
| ENSRNOG00000004249 | Tlr7 | 1.30 | 0.29 | up |
| ENSRNOG00000016117 | Myof | 1.05 | 0.24 | up |
| ENSRNOG00000019741 | Isyna1 | 46.57 | 11.10 | up |
| ENSRNOG00000052795 | Itpr3 | 0.51 | 0.11 | up |
| ENSRNOG00000047606 | Bcl2a1 | 14.44 | 3.39 | up |
| ENSRNOG00000020781 | Tbcb | 14.03 | 3.31 | up |
| ENSRNOG00000023781 | Plec | 4.27 | 1.02 | up |
| ENSRNOG00000037371 | Xaf1 | 10.84 | 2.53 | up |
| ENSRNOG00000026605 | Ifi27l2b | 1487.64 | 358.43 | up |
| ENSRNOG00000004821 | Sntb1 | 2.71 | 0.63 | up |
| ENSRNOG00000020587 | Efemp2 | 6.08 | 1.43 | up |
| ENSRNOG00000001252 | Chst12 | 2.82 | 0.64 | up |
| ENSRNOG00000018257 | Hpx | 19089.65 | 4643.73 | up |
| ENSRNOG00000001908 | Klhl6 | 1.31 | 0.30 | up |
| ENSRNOG00000021750 | Id1 | 11.21 | 2.65 | up |
| ENSRNOG00000010997 | Ednrb | 7.03 | 1.68 | up |
| ENSRNOG00000046968 | Nol8 | 1.52 | 0.36 | up |
| ENSRNOG00000009513 | Akr1b1 | 15.15 | 3.65 | up |
| ENSRNOG00000042929 | LOC100361087 | 4.67 | 1.11 | up |
| ENSRNOG00000029682 | Clic1 | 29.18 | 7.11 | up |
| ENSRNOG00000000768 | Ubd | 114.13 | 28.02 | up |
| ENSRNOG00000054764 | Flt3 | 1.40 | 0.32 | up |
| ENSRNOG00000048771 | RGD1559482 | 6.75 | 1.63 | up |
| ENSRNOG00000011015 | Hivep2 | 0.97 | 0.23 | up |
| ENSRNOG00000003538 | Adamts4 | 0.97 | 0.23 | up |
| ENSRNOG00000020032 | Impdh1 | 3.94 | 0.95 | up |
| ENSRNOG00000001379 | Cyp3a62 | 22.27 | 5.48 | up |
| ENSRNOG00000014336 | Mcm5 | 3.25 | 0.78 | up |
| ENSRNOG00000061403 | AABR07039446 | 1.87 | 0.45 | up |
| ENSRNOG00000009822 | Tlr2 | 2.78 | 0.67 | up |
| ENSRNOG00000021161 | Fermt3 | 16.02 | 3.98 | up |
| ENSRNOG00000002776 | Sell | 2.55 | 0.60 | up |
| ENSRNOG00000025764 | AC128848 | 954.03 | 239.31 | up |
| ENSRNOG00000003486 | Mnda | 10.86 | 2.68 | up |
| ENSRNOG00000025164 | Bhlha15 | 4.29 | 1.06 | up |
| ENSRNOG00000000187 | Csf2rb | 6.35 | 1.58 | up |
| ENSRNOG00000017429 | Lat | 5.05 | 1.22 | up |
| ENSRNOG00000013102 | Entpd2 | 3.23 | 0.78 | up |
| ENSRNOG00000004972 | Upp1 | 4.13 | 1.01 | up |
| ENSRNOG00000000596 | Fyn | 5.72 | 1.43 | up |
| ENSRNOG00000008144 | Irf1 | 19.89 | 4.99 | up |
| ENSRNOG00000010165 | Tnfaip2 | 21.96 | 5.55 | up |
| ENSRNOG00000032703 | Rasgrp3 | 3.40 | 0.85 | up |
| ENSRNOG00000019142 | Fas | 7.48 | 1.88 | up |
| ENSRNOG00000045924 | RT1-T24-3 | 13.95 | 3.51 | up |
| ENSRNOG00000037113 | Slfn2 | 21.79 | 5.50 | up |
| ENSRNOG00000005341 | Upp2 | 4.63 | 1.15 | up |
| ENSRNOG00000005906 | LOC103690020 | 2.60 | 0.64 | up |
| ENSRNOG00000018877 | Zfp629 | 1.85 | 0.46 | up |
| ENSRNOG00000048411 | Uhrf1 | 1.52 | 0.37 | up |
| ENSRNOG00000013736 | C9 | 1954.69 | 499.72 | up |
| ENSRNOG00000004003 | Dusp10 | 1.67 | 0.41 | up |
| ENSRNOG00000024277 | AABR07035470 | 1.37 | 0.33 | up |
| ENSRNOG00000043044 | Cnn2 | 10.69 | 2.67 | up |
| ENSRNOG00000019316 | Sh3bp4 | 4.25 | 1.08 | up |
| ENSRNOG00000018371 | Tubb6 | 12.19 | 3.11 | up |
| ENSRNOG00000006108 | Gngt2 | 19.74 | 4.97 | up |
| ENSRNOG00000013889 | Tmed3 | 21.83 | 5.60 | up |
| ENSRNOG00000008016 | Ckap4 | 5.06 | 1.28 | up |
| ENSRNOG00000031406 | Hps3 | 12.23 | 3.15 | up |
| ENSRNOG00000018413 | Per3 | 1.20 | 0.30 | up |
| ENSRNOG00000010794 | Dennd3 | 1.17 | 0.29 | up |
| ENSRNOG00000003666 | Jchain | 27.54 | 7.09 | up |
| ENSRNOG00000042499 | LOC100364435 | 243.67 | 63.90 | up |
| ENSRNOG00000020465 | Ripk3 | 5.39 | 1.39 | up |
| ENSRNOG00000014964 | Hp | 23245.03 | 6121.94 | up |
| ENSRNOG00000016541 | Enc1 | 18.71 | 4.92 | up |
| ENSRNOG00000010319 | Lcp1 | 19.86 | 5.22 | up |
| ENSRNOG00000024846 | Ier5l | 2.57 | 0.64 | up |
| ENSRNOG00000013954 | Alpl | 13.38 | 3.51 | up |
| ENSRNOG00000004019 | Phlda1 | 17.90 | 4.70 | up |
| ENSRNOG00000019737 | Sema4a | 5.06 | 1.33 | up |
| ENSRNOG00000026702 | Jaml | 1.75 | 0.44 | up |
| ENSRNOG00000013526 | Rassf4 | 6.81 | 1.78 | up |
| ENSRNOG00000021102 | Scn1b | 9.78 | 2.56 | up |
| ENSRNOG00000042344 | Smim22 | 8.02 | 2.05 | up |
| ENSRNOG00000008301 | Tagln2 | 80.79 | 21.58 | up |
| ENSRNOG00000019311 | Nfkb2 | 12.10 | 3.22 | up |
| ENSRNOG00000007300 | C1qtnf6 | 1.38 | 0.35 | up |
| ENSRNOG00000030118 | Msn | 21.65 | 5.78 | up |
| ENSRNOG00000021104 | Emp3 | 12.37 | 3.23 | up |
| ENSRNOG00000051977 | Mmrn2 | 5.40 | 1.43 | up |
| ENSRNOG00000013176 | Far1 | 1.54 | 0.40 | up |
| ENSRNOG00000004660 | Fzd6 | 2.23 | 0.59 | up |
| ENSRNOG00000014333 | Vcam1 | 6.37 | 1.70 | up |
| ENSRNOG00000013970 | Cdt1 | 1.72 | 0.43 | up |
| ENSRNOG00000013727 | Ndc80 | 1.13 | 0.29 | up |
| ENSRNOG00000004111 | Soat1 | 3.14 | 0.81 | up |
| ENSRNOG00000018092 | Cd83 | 2.94 | 0.77 | up |
| ENSRNOG00000010922 | Ppp2r1b | 3.17 | 0.84 | up |
| ENSRNOG00000019550 | Slc11a2 | 17.40 | 4.72 | up |
| ENSRNOG00000051986 | Plcg2 | 2.08 | 0.55 | up |
| ENSRNOG00000015941 | Fkbp10 | 1.70 | 0.44 | up |
| ENSRNOG00000016163 | Slc1a3 | 1.41 | 0.37 | up |
| ENSRNOG00000007178 | Cd8a | 4.99 | 1.32 | up |
| ENSRNOG00000008415 | Nab2 | 5.15 | 1.41 | up |
| ENSRNOG00000011821 | S100a4 | 11.95 | 3.19 | up |
| ENSRNOG00000004424 | RGD1563962 | 2.36 | 0.63 | up |
| ENSRNOG00000043182 | Septin6 | 1.37 | 0.36 | up |
| ENSRNOG00000061231 | Selenom | 9.33 | 2.49 | up |
| ENSRNOG00000005929 | Them6 | 12.72 | 3.48 | up |
| ENSRNOG00000050430 | Vav1 | 3.90 | 1.05 | up |
| ENSRNOG00000015078 | Ifitm3 | 361.14 | 100.10 | up |
| ENSRNOG00000013805 | Tnip2 | 5.65 | 1.53 | up |
| ENSRNOG00000007726 | Mcam | 5.77 | 1.58 | up |
| ENSRNOG00000019810 | Des | 5.68 | 1.55 | up |
| ENSRNOG00000046535 | Ppm1m | 1.78 | 0.46 | up |
| ENSRNOG00000046393 | Fau | 97.36 | 26.97 | up |
| ENSRNOG00000013313 | Nceh1 | 7.92 | 2.20 | up |
| ENSRNOG00000017512 | Aldh3b1 | 1.78 | 0.47 | up |
| ENSRNOG00000010111 | Exoc3l4 | 1.48 | 0.40 | up |
| ENSRNOG00000030930 | Samsn1 | 3.77 | 1.02 | up |
| ENSRNOG00000000763 | RT1-M3-1 | 4.48 | 1.22 | up |
| ENSRNOG00000016378 | Map3k8 | 3.08 | 0.85 | up |
| ENSRNOG00000013121 | Mier3 | 1.82 | 0.50 | up |
| ENSRNOG00000033361 | Slc39a5 | 2.57 | 0.71 | up |
| ENSRNOG00000020679 | Icam1 | 36.11 | 10.29 | up |
| ENSRNOG00000030027 | Fbxw17 | 2.13 | 0.57 | up |
| ENSRNOG00000004322 | Sh3kbp1 | 1.55 | 0.43 | up |
| ENSRNOG00000012630 | Rhoc | 8.78 | 2.48 | up |
| ENSRNOG00000017680 | Dennd2d | 2.41 | 0.66 | up |
| ENSRNOG00000033772 | Serpinb9 | 30.05 | 8.62 | up |
| ENSRNOG00000011296 | Cenpn | 1.50 | 0.40 | up |
| ENSRNOG00000048402 | Igh-6 | 22.22 | 6.35 | up |
| ENSRNOG00000014509 | Sacs | 0.53 | 0.15 | up |
| ENSRNOG00000022533 | Micall2 | 1.16 | 0.31 | up |
| ENSRNOG00000015668 | Ccl19 | 4.85 | 1.32 | up |
| ENSRNOG00000017445 | Tubb2b | 3.82 | 1.07 | up |
| ENSRNOG00000019943 | Slc7a6 | 1.44 | 0.39 | up |
| ENSRNOG00000011723 | Slc44a3 | 2.77 | 0.76 | up |
| ENSRNOG00000007164 | Cln6 | 5.81 | 1.65 | up |
| ENSRNOG00000053691 | Lama5 | 0.67 | 0.19 | up |
| ENSRNOG00000009920 | LOC680121 | 67.88 | 19.68 | up |
| ENSRNOG00000002385 | Prg4 | 75.46 | 21.90 | up |
| ENSRNOG00000034200 | Atp8a1 | 1.82 | 0.51 | up |
| ENSRNOG00000019118 | Slc13a3 | 34.61 | 10.07 | up |
| ENSRNOG00000051291 | Dnhd1 | 1.09 | 0.31 | up |
| ENSRNOG00000008539 | Lgals2 | 9.61 | 2.69 | up |
| ENSRNOG00000004040 | Abca8 | 4.21 | 1.21 | up |
| ENSRNOG00000047657 | C4a | 137.88 | 40.21 | up |
| ENSRNOG00000030154 | Cyp4a2 | 146.16 | 42.61 | up |
| ENSRNOG00000016257 | Cotl1 | 29.64 | 8.61 | up |
| ENSRNOG00000045829 | Thbs1 | 3.05 | 0.88 | up |
| ENSRNOG00000013220 | Arhgap45 | 8.82 | 2.56 | up |
| ENSRNOG00000019058 | Gstm3l | 14.98 | 4.26 | up |
| ENSRNOG00000034198 | Tceal9 | 22.87 | 6.63 | up |
| ENSRNOG00000025584 | Agap2 | 1.10 | 0.31 | up |
| ENSRNOG00000003464 | Hid1 | 4.50 | 1.30 | up |
| ENSRNOG00000010549 | Tspo | 20.47 | 5.93 | up |
| ENSRNOG00000009341 | Hivep3 | 0.33 | 0.09 | up |
| ENSRNOG00000013194 | Rps6ka2 | 1.05 | 0.30 | up |
| ENSRNOG00000019662 | Tm6sf1 | 1.31 | 0.37 | up |
| ENSRNOG00000018087 | Vim | 52.10 | 15.42 | up |
| ENSRNOG00000057125 | Ddr1 | 0.96 | 0.27 | up |
| ENSRNOG00000005608 | Tead4 | 1.08 | 0.31 | up |
| ENSRNOG00000008409 | Myo1f | 5.06 | 1.49 | up |
| ENSRNOG00000033608 | Cd276 | 4.07 | 1.20 | up |
| ENSRNOG00000002403 | Niban1 | 1.21 | 0.35 | up |
| ENSRNOG00000027722 | H1f10 | 14.18 | 4.14 | up |
| ENSRNOG00000010646 | Tmem229b | 2.35 | 0.69 | up |
| ENSRNOG00000002396 | Serpinb8 | 1.93 | 0.57 | up |
| ENSRNOG00000006116 | Hk2 | 1.13 | 0.33 | up |
| ENSRNOG00000049047 | LOC690468 | 21.37 | 6.20 | up |
| ENSRNOG00000021724 | Ptprcap | 5.38 | 1.57 | up |
| ENSRNOG00000008628 | Ica1 | 3.07 | 0.90 | up |
| ENSRNOG00000003546 | Tnfrsf12a | 10.57 | 3.12 | up |
| ENSRNOG00000003657 | Pkmyt1 | 3.22 | 0.96 | up |
| ENSRNOG00000031233 | Mapk12 | 3.39 | 0.99 | up |
| ENSRNOG00000000655 | Ptprc | 6.55 | 1.97 | up |
| ENSRNOG00000015157 | Smtnl2 | 2.03 | 0.59 | up |
| ENSRNOG00000019556 | Cd9 | 4.10 | 1.20 | up |
| ENSRNOG00000008048 | Plscr1 | 3.59 | 1.06 | up |
| ENSRNOG00000020652 | Tgfb1 | 15.39 | 4.66 | up |
| ENSRNOG00000000787 | AABR07044364 | 3.89 | 1.11 | up |
| ENSRNOG00000025843 | Ccdc102a | 1.94 | 0.56 | up |
| ENSRNOG00000015442 | Sfxn3 | 2.99 | 0.89 | up |
| ENSRNOG00000002217 | Plac8 | 37.03 | 11.22 | up |
| ENSRNOG00000007765 | Frzb | 2.01 | 0.59 | up |
| ENSRNOG00000009385 | Pik3cg | 0.87 | 0.25 | up |
| ENSRNOG00000009331 | Hck | 10.46 | 3.17 | up |
| ENSRNOG00000038955 | MGC105567 | 2.75 | 0.82 | up |
| ENSRNOG00000004192 | Arhgap30 | 3.22 | 0.97 | up |
| ENSRNOG00000002930 | Ppl | 0.92 | 0.27 | up |
| ENSRNOG00000039848 | Ak6 | 15.80 | 4.83 | up |
| ENSRNOG00000012294 | Heph | 0.78 | 0.22 | up |
| ENSRNOG00000045997 | Chst14 | 1.79 | 0.53 | up |
| ENSRNOG00000011647 | S100a6 | 20.25 | 6.15 | up |
| ENSRNOG00000023143 | Nlrp1a | 1.18 | 0.36 | up |
| ENSRNOG00000005190 | Nipal2 | 9.06 | 2.80 | up |
| ENSRNOG00000019661 | Gdf15 | 37.15 | 11.49 | up |
| ENSRNOG00000042137 | Sting1 | 2.32 | 0.70 | up |
| ENSRNOG00000005126 | Slc66a3 | 2.36 | 0.70 | up |
| ENSRNOG00000012860 | Tmem184c | 2.70 | 0.82 | up |
| ENSRNOG00000014237 | Zfp503 | 2.92 | 0.89 | up |
| ENSRNOG00000018536 | Pck2 | 10.70 | 3.31 | up |
| ENSRNOG00000016343 | Dkk3 | 1.22 | 0.37 | up |
| ENSRNOG00000018716 | Dennd2c | 0.69 | 0.21 | up |
| ENSRNOG00000012820 | Add3 | 4.66 | 1.45 | up |
| ENSRNOG00000021735 | Akr1c15 | 13.84 | 4.32 | up |
| ENSRNOG00000003915 | Pacc1 | 2.74 | 0.82 | up |
| ENSRNOG00000029377 | Rpl38 | 19.90 | 6.01 | up |
| ENSRNOG00000046601 | Unc5cl | 3.32 | 1.04 | up |
| ENSRNOG00000001302 | Adora2a | 6.43 | 2.01 | up |
| ENSRNOG00000016225 | Fgd3 | 1.53 | 0.47 | up |
| ENSRNOG00000020415 | Ramp2 | 19.04 | 5.98 | up |
| ENSRNOG00000016756 | Ptgir | 1.00 | 0.30 | up |
| ENSRNOG00000062084 | LOC680491 | 2.11 | 0.64 | up |
| ENSRNOG00000038035 | Kif4a | 0.94 | 0.29 | up |
| ENSRNOG00000006557 | Cyfip2 | 2.00 | 0.63 | up |
| ENSRNOG00000045560 | Gvin1 | 0.60 | 0.18 | up |
| ENSRNOG00000013250 | Pdcd5 | 9.91 | 3.07 | up |
| ENSRNOG00000022910 | Emcn | 8.14 | 2.58 | up |
| ENSRNOG00000010219 | Ralgds | 3.65 | 1.16 | up |
| ENSRNOG00000020310 | Grik5 | 1.06 | 0.32 | up |
| ENSRNOG00000021919 | Rhoj | 3.22 | 1.02 | up |
| ENSRNOG00000052802 | Aldoa | 96.24 | 31.02 | up |
| ENSRNOG00000060052 | Tdrd15 | 1.21 | 0.38 | up |
| ENSRNOG00000012181 | Lpl | 6.52 | 2.09 | up |
| ENSRNOG00000014504 | Il1r1 | 33.80 | 10.91 | up |
| ENSRNOG00000016071 | P3h3 | 7.04 | 2.25 | up |
| ENSRNOG00000001652 | Erg | 4.72 | 1.51 | up |
| ENSRNOG00000011559 | Cnn3 | 110.98 | 35.97 | up |
| ENSRNOG00000037509 | Chek2 | 2.46 | 0.77 | up |
| ENSRNOG00000017021 | Galnt18 | 2.18 | 0.69 | up |
| ENSRNOG00000001976 | Tmprss2 | 3.64 | 1.17 | up |
| ENSRNOG00000001250 | Lfng | 2.55 | 0.80 | up |
| ENSRNOG00000022636 | Alpk1 | 1.54 | 0.49 | up |
| ENSRNOG00000001201 | Cstb | 73.37 | 23.88 | up |
| ENSRNOG00000015670 | Stx7 | 6.62 | 2.14 | up |
| ENSRNOG00000017416 | Ppic | 2.97 | 0.92 | up |
| ENSRNOG00000043094 | Oxct1 | 2.77 | 0.89 | up |
| ENSRNOG00000013251 | Trim24 | 2.65 | 0.84 | up |
| ENSRNOG00000008283 | B4galt5 | 4.17 | 1.35 | up |
| ENSRNOG00000002599 | Grap | 3.78 | 1.20 | up |
| ENSRNOG00000007906 | Bub1b | 0.95 | 0.30 | up |
| ENSRNOG00000020845 | Tyrobp | 40.75 | 13.31 | up |
| ENSRNOG00000012513 | Pdk3 | 1.47 | 0.46 | up |
| ENSRNOG00000019698 | Ssbp4 | 9.04 | 2.92 | up |
| ENSRNOG00000013917 | Igsf10 | 1.38 | 0.45 | up |
| ENSRNOG00000014251 | Capn5 | 2.66 | 0.86 | up |
| ENSRNOG00000042607 | Rhof | 2.92 | 0.94 | up |
| ENSRNOG00000057092 | Slfn4 | 26.26 | 8.67 | up |
| ENSRNOG00000002440 | Ralb | 11.16 | 3.67 | up |
| ENSRNOG00000015242 | Fbxl8 | 1.83 | 0.58 | up |
| ENSRNOG00000051922 | AABR07059663 | 3.74 | 1.23 | up |
| ENSRNOG00000058739 | Snn | 2.04 | 0.66 | up |
| ENSRNOG00000006231 | Ptpro | 0.84 | 0.27 | up |
| ENSRNOG00000010872 | Ckb | 19.77 | 6.54 | up |
| ENSRNOG00000021029 | Hamp | 1881.32 | 626.36 | up |
| ENSRNOG00000016012 | Spats2l | 2.30 | 0.75 | up |
| ENSRNOG00000046050 | Dennd1c | 1.51 | 0.48 | up |
| ENSRNOG00000011321 | Rftn1 | 1.97 | 0.63 | up |
| ENSRNOG00000032585 | AABR07048992 | 35.20 | 11.73 | up |
| ENSRNOG00000029370 | Abhd3 | 5.43 | 1.79 | up |
| ENSRNOG00000006984 | Mapk11 | 1.87 | 0.60 | up |
| ENSRNOG00000008481 | Reep1 | 0.95 | 0.30 | up |
| ENSRNOG00000014137 | Fbln1 | 1.55 | 0.50 | up |
| ENSRNOG00000016281 | Col4a1 | 24.16 | 8.10 | up |
| ENSRNOG00000052734 | AABR07051310 | 5.55 | 1.77 | up |
| ENSRNOG00000000699 | Selplg | 8.73 | 2.88 | up |
| ENSRNOG00000020531 | Fen1 | 4.30 | 1.43 | up |
| ENSRNOG00000055010 | Axin2 | 4.43 | 1.48 | up |
| ENSRNOG00000010265 | Ada | 5.76 | 1.90 | up |
| ENSRNOG00000011589 | Camk2d | 2.04 | 0.67 | up |
| ENSRNOG00000021510 | Tbc1d10c | 1.79 | 0.57 | up |
| ENSRNOG00000003259 | C1qtnf1 | 8.93 | 3.01 | up |
| ENSRNOG00000047800 | C5ar1 | 3.75 | 1.23 | up |
| ENSRNOG00000025810 | Grcc10 | 23.78 | 7.97 | up |
| ENSRNOG00000018781 | Map1s | 2.21 | 0.73 | up |
| ENSRNOG00000000827 | Ier3 | 34.35 | 11.60 | up |
| ENSRNOG00000010753 | Aig1 | 3.99 | 1.31 | up |
| ENSRNOG00000010392 | Nrg1 | 1.21 | 0.40 | up |
| ENSRNOG00000018729 | Rad9a | 3.50 | 1.13 | up |
| ENSRNOG00000050885 | LOC100910526 | 2.08 | 0.69 | up |
| ENSRNOG00000059705 | Elmo1 | 1.42 | 0.46 | up |
| ENSRNOG00000026748 | Dennd2a | 7.42 | 2.51 | up |
| ENSRNOG00000057335 | Clec1b | 6.07 | 2.02 | up |
| ENSRNOG00000033747 | Sp110 | 12.63 | 4.28 | up |
| ENSRNOG00000028548 | Ccl9 | 1075.31 | 367.48 | up |
| ENSRNOG00000036701 | Actg1 | 270.82 | 92.91 | up |
| ENSRNOG00000023972 | Col4a2 | 15.02 | 5.15 | up |
| ENSRNOG00000004409 | Sash3 | 2.55 | 0.85 | up |
| ENSRNOG00000016148 | Gtse1 | 1.74 | 0.58 | up |
| ENSRNOG00000019831 | Samd4b | 4.57 | 1.55 | up |
| ENSRNOG00000008050 | Stac3 | 2.20 | 142.29 | down |
| ENSRNOG00000001388 | Sds | 3.70 | 82.38 | down |
| ENSRNOG00000038132 | Vsig4 | 0.90 | 17.14 | down |
| ENSRNOG00000019358 | Esr1 | 0.26 | 3.97 | down |
| ENSRNOG00000013137 | Clec4f | 4.62 | 65.82 | down |
| ENSRNOG00000042721 | Gimd1 | 0.79 | 9.26 | down |
| ENSRNOG00000014387 | Chac1 | 2.07 | 23.36 | down |
| ENSRNOG00000045877 | AC098547 | 0.86 | 8.09 | down |
| ENSRNOG00000011420 | Mtmr7 | 0.35 | 3.49 | down |
| ENSRNOG00000003634 | Zfp354a | 2.97 | 26.12 | down |
| ENSRNOG00000055909 | Apoa4 | 82.30 | 704.07 | down |
| ENSRNOG00000010253 | Cd163 | 0.86 | 7.14 | down |
| ENSRNOG00000047356 | LOC100909750 | 0.27 | 2.26 | down |
| ENSRNOG00000016222 | Ppp1r3g | 1.65 | 13.73 | down |
| ENSRNOG00000010799 | Noct | 1.37 | 11.03 | down |
| ENSRNOG00000013552 | Scd | 68.08 | 524.16 | down |
| ENSRNOG00000003244 | Ltc4s | 2.78 | 20.47 | down |
| ENSRNOG00000051081 | AABR07048439 | 144.47 | 991.75 | down |
| ENSRNOG00000054489 | AABR07042821 | 10.66 | 71.33 | down |
| ENSRNOG00000011200 | Bhmt | 266.95 | 1581.68 | down |
| ENSRNOG00000017063 | Fcna | 3.28 | 18.75 | down |
| ENSRNOG00000004502 | Hal | 16.53 | 92.23 | down |
| ENSRNOG00000025648 | Dhrs7l1 | 57.42 | 313.88 | down |
| ENSRNOG00000052810 | Cyp2c11 | 247.16 | 1325.12 | down |
| ENSRNOG00000029980 | Zbtb16 | 0.67 | 3.70 | down |
| ENSRNOG00000014338 | Slc25a25 | 31.57 | 163.71 | down |
| ENSRNOG00000010079 | Ca3 | 421.00 | 2127.96 | down |
| ENSRNOG00000045743 | Etnppl | 5.64 | 28.20 | down |
| ENSRNOG00000048194 | LOC100912380 | 10.42 | 52.52 | down |
| ENSRNOG00000038610 | AABR07034445 | 10.69 | 53.13 | down |
| ENSRNOG00000054930 | AABR07004572 | 1.69 | 8.47 | down |
| ENSRNOG00000015858 | Hyal1 | 3.24 | 16.05 | down |
| ENSRNOG00000005266 | Amdhd1 | 3.80 | 18.62 | down |
| ENSRNOG00000027784 | Tsku | 39.81 | 190.69 | down |
| ENSRNOG00000046643 | Cyp3a9 | 2.47 | 12.03 | down |
| ENSRNOG00000016037 | Mafb | 7.71 | 36.57 | down |
| ENSRNOG00000023068 | Cd5l | 9.66 | 45.53 | down |
| ENSRNOG00000029670 | RGD1564606 | 8.18 | 39.27 | down |
| ENSRNOG00000017693 | Slc2a5 | 0.72 | 3.45 | down |
| ENSRNOG00000037188 | Mug1 | 335.74 | 1510.00 | down |
| ENSRNOG00000018287 | RGD1307603 | 97.17 | 437.07 | down |
| ENSRNOG00000001113 | Mmd2 | 0.60 | 2.84 | down |
| ENSRNOG00000049303 | Marco | 5.70 | 25.15 | down |
| ENSRNOG00000000561 | Pald1 | 4.52 | 19.39 | down |
| ENSRNOG00000020467 | Nrep | 20.20 | 87.13 | down |
| ENSRNOG00000014008 | Mfsd2a | 15.10 | 64.09 | down |
| ENSRNOG00000004377 | Lpin1 | 8.21 | 33.95 | down |
| ENSRNOG00000007607 | Nr4a1 | 3.56 | 14.61 | down |
| ENSRNOG00000013408 | Npas2 | 1.46 | 6.03 | down |
| ENSRNOG00000056135 | Tsc22d3 | 22.97 | 92.49 | down |
| ENSRNOG00000013090 | Gadd45g | 33.04 | 132.00 | down |
| ENSRNOG00000014678 | Fzd5 | 0.53 | 2.22 | down |
| ENSRNOG00000049489 | LOC102552166 | 0.48 | 1.97 | down |
| ENSRNOG00000061527 | Gck | 5.86 | 22.40 | down |
| ENSRNOG00000061821 | AC109891 | 0.87 | 3.38 | down |
| ENSRNOG00000011635 | Ces2e | 6.41 | 24.41 | down |
| ENSRNOG00000038970 | AABR07037410 | 0.25 | 1.02 | down |
| ENSRNOG00000043131 | LOC100360095 | 697.88 | 2632.36 | down |
| ENSRNOG00000009921 | Serpina3m | 60.27 | 224.71 | down |
| ENSRNOG00000033057 | AABR07025010 | 9.07 | 33.27 | down |
| ENSRNOG00000002212 | Hsd17b13 | 37.43 | 134.52 | down |
| ENSRNOG00000020704 | Tkfc | 27.47 | 98.68 | down |
| ENSRNOG00000056894 | St6galnac3 | 0.34 | 1.32 | down |
| ENSRNOG00000045967 | AABR07064061 | 2.64 | 9.62 | down |
| ENSRNOG00000004403 | Slc25a32 | 4.00 | 14.11 | down |
| ENSRNOG00000002345 | Rasgef1b | 4.55 | 15.90 | down |
| ENSRNOG00000006331 | Elovl5 | 59.01 | 202.71 | down |
| ENSRNOG00000030021 | Ccl6 | 20.73 | 70.84 | down |
| ENSRNOG00000019659 | Aspa | 1.23 | 4.30 | down |
| ENSRNOG00000013925 | Nox4 | 3.08 | 10.34 | down |
| ENSRNOG00000018606 | Olr59 | 3.69 | 12.28 | down |
| ENSRNOG00000061876 | Tas1r2 | 16.25 | 53.57 | down |
| ENSRNOG00000022268 | Pnpla3 | 2.24 | 7.46 | down |
| ENSRNOG00000018494 | Ppp1r3c | 23.90 | 78.28 | down |
| ENSRNOG00000021130 | Abcc8 | 0.34 | 1.16 | down |
| ENSRNOG00000048623 | LOC100911564 | 24.42 | 79.90 | down |
| ENSRNOG00000033245 | Mug2 | 49.15 | 159.77 | down |
| ENSRNOG00000010325 | Ptger3 | 2.46 | 8.05 | down |
| ENSRNOG00000012807 | C1qa | 23.13 | 74.49 | down |
| ENSRNOG00000017601 | Srd5a1 | 38.32 | 120.96 | down |
| ENSRNOG00000010625 | Dnmt3b | 0.46 | 1.50 | down |
| ENSRNOG00000029478 | Cyp4f39 | 4.16 | 13.01 | down |
| ENSRNOG00000005420 | Abcg8 | 0.58 | 1.85 | down |
| ENSRNOG00000004327 | Ddc | 5.93 | 18.40 | down |
| ENSRNOG00000012274 | Ddi2 | 8.24 | 25.54 | down |
| ENSRNOG00000033915 | Gpt | 15.09 | 46.73 | down |
| ENSRNOG00000032374 | Paqr9 | 27.51 | 84.19 | down |
| ENSRNOG00000014456 | Coq10b | 13.37 | 40.70 | down |
| ENSRNOG00000003977 | Dusp1 | 72.07 | 217.89 | down |
| ENSRNOG00000024907 | Tnrc6b | 0.63 | 1.91 | down |
| ENSRNOG00000054459 | Mboat7 | 1.77 | 5.46 | down |
| ENSRNOG00000011820 | Acpp | 1.55 | 4.67 | down |
| ENSRNOG00000013771 | Clca2 | 1.32 | 3.94 | down |
| ENSRNOG00000023657 | Gprin3 | 1.11 | 3.34 | down |
| ENSRNOG00000062125 | Aox3 | 7.09 | 20.87 | down |
| ENSRNOG00000031612 | Gls2 | 21.37 | 62.84 | down |
| ENSRNOG00000017311 | Me3 | 1.53 | 4.59 | down |
| ENSRNOG00000007410 | Dab1 | 0.63 | 1.89 | down |
| ENSRNOG00000004100 | Trib1 | 7.22 | 21.22 | down |
| ENSRNOG00000051171 | G6pc | 183.52 | 535.68 | down |
| ENSRNOG00000010188 | Satb2 | 0.21 | 0.64 | down |
| ENSRNOG00000011815 | Sgk1 | 9.12 | 26.43 | down |
| ENSRNOG00000020444 | Hcn3 | 1.65 | 4.77 | down |
| ENSRNOG00000019319 | Fchsd2 | 3.39 | 9.78 | down |
| ENSRNOG00000007591 | Slc45a3 | 2.96 | 8.51 | down |
| ENSRNOG00000046727 | Abcc2 | 33.53 | 95.12 | down |
| ENSRNOG00000001052 | Slc25a30 | 2.65 | 7.62 | down |
| ENSRNOG00000032805 | Cyp2f4 | 10.84 | 30.70 | down |
| ENSRNOG00000011250 | Inmt | 2.12 | 6.14 | down |
| ENSRNOG00000036318 | AC119336 | 57.22 | 161.28 | down |
| ENSRNOG00000006859 | Insig1 | 138.79 | 386.13 | down |
| ENSRNOG00000060946 | Cask | 0.68 | 1.94 | down |
| ENSRNOG00000012843 | Aspg | 14.20 | 39.48 | down |
| ENSRNOG00000017100 | Yme1l1 | 2.99 | 8.38 | down |
| ENSRNOG00000031979 | Mt-atp6 | 3622.21 | 10002.28 | down |
| ENSRNOG00000016488 | Pltp | 5.23 | 14.56 | down |
| ENSRNOG00000006972 | Zfp189 | 4.88 | 13.52 | down |
| ENSRNOG00000005758 | Btbd11 | 0.53 | 1.52 | down |
| ENSRNOG00000011474 | Ppp1r3b | 19.28 | 52.72 | down |
| ENSRNOG00000059519 | Bbox1 | 12.23 | 33.45 | down |
| ENSRNOG00000007964 | Tp53inp1 | 2.96 | 8.22 | down |
| ENSRNOG00000009438 | Serpina6 | 126.87 | 344.33 | down |
| ENSRNOG00000004245 | Mgst3 | 38.71 | 105.31 | down |
| ENSRNOG00000013950 | Aadac | 19.43 | 52.50 | down |
| ENSRNOG00000049471 | Steap3 | 5.53 | 14.94 | down |
| ENSRNOG00000014508 | Mgll | 7.34 | 19.76 | down |
| ENSRNOG00000011016 | Slc7a2 | 14.78 | 39.29 | down |
| ENSRNOG00000000503 | Ppard | 3.43 | 9.11 | down |
| ENSRNOG00000043404 | Uroc1 | 43.28 | 114.48 | down |
| ENSRNOG00000062247 | AABR07031521 | 4.83 | 12.87 | down |
| ENSRNOG00000057089 | LOC103691744 | 60.12 | 158.82 | down |
| ENSRNOG00000040289 | Xkr9 | 3.20 | 8.52 | down |
| ENSRNOG00000013570 | Rad54l2 | 0.82 | 2.17 | down |
| ENSRNOG00000014900 | Crem | 2.62 | 6.88 | down |
| ENSRNOG00000014090 | Retsat | 119.97 | 312.64 | down |
| ENSRNOG00000049848 | Rpl21 | 4.53 | 12.15 | down |
| ENSRNOG00000012098 | Adcyap1r1 | 0.52 | 1.37 | down |
| ENSRNOG00000016348 | Tat | 305.46 | 788.83 | down |
| ENSRNOG00000016356 | Got1 | 47.85 | 123.41 | down |
| ENSRNOG00000060348 | Zdhhc23 | 1.78 | 4.62 | down |
| ENSRNOG00000030719 | Csmd1 | 0.23 | 0.62 | down |
| ENSRNOG00000046254 | Adgre1 | 1.87 | 4.85 | down |
| ENSRNOG00000006305 | Slc38a2 | 22.64 | 57.87 | down |
| ENSRNOG00000051232 | Gbe1 | 33.94 | 86.61 | down |
| ENSRNOG00000042543 | RGD1566134 | 1377.23 | 3504.50 | down |
| ENSRNOG00000008638 | Angptl3 | 150.77 | 383.31 | down |
| ENSRNOG00000058327 | AABR07052458 | 2.64 | 6.93 | down |
| ENSRNOG00000040195 | RGD1559960 | 5.88 | 14.93 | down |
| ENSRNOG00000003370 | Otc | 93.99 | 235.74 | down |
| ENSRNOG00000009322 | Ccdc126 | 2.69 | 6.82 | down |
| ENSRNOG00000014426 | Lox | 0.94 | 2.37 | down |
| ENSRNOG00000000521 | Cdkn1a | 13.04 | 32.63 | down |
| ENSRNOG00000019098 | Ca5a | 12.29 | 30.73 | down |
| ENSRNOG00000000201 | Gsta5 | 412.02 | 1025.37 | down |
| ENSRNOG00000015124 | Gpam | 10.97 | 27.32 | down |
| ENSRNOG00000042785 | Sesn2 | 7.87 | 19.59 | down |
| ENSRNOG00000030269 | Atp2b2 | 1.05 | 2.62 | down |
| ENSRNOG00000006180 | Pum2 | 4.00 | 9.93 | down |
| ENSRNOG00000006675 | Fabp1 | 1736.37 | 4291.38 | down |
| ENSRNOG00000008095 | Onecut1 | 1.22 | 3.06 | down |
| ENSRNOG00000020440 | Fads2 | 111.55 | 273.99 | down |
| ENSRNOG00000010887 | RGD1309534 | 12.90 | 31.69 | down |
| ENSRNOG00000009019 | Slc6a6 | 2.17 | 5.37 | down |
| ENSRNOG00000012040 | Slc25a48 | 1.33 | 3.44 | down |
| ENSRNOG00000009170 | Dmxl2 | 1.33 | 3.26 | down |
| ENSRNOG00000001030 | Tsc22d1 | 17.52 | 42.66 | down |
| ENSRNOG00000015036 | Ccn2 | 2.06 | 5.07 | down |
| ENSRNOG00000003749 | Xk | 0.72 | 1.76 | down |
| ENSRNOG00000001621 | C2cd2 | 4.99 | 12.08 | down |
| ENSRNOG00000046468 | Ptgfr | 1.36 | 3.34 | down |
| ENSRNOG00000032394 | Tymp | 68.47 | 164.34 | down |
| ENSRNOG00000000070 | LOC100910207 | 3.69 | 8.90 | down |
| ENSRNOG00000037230 | Ppidl1 | 1.49 | 3.68 | down |
| ENSRNOG00000034116 | Gk | 34.78 | 82.93 | down |
| ENSRNOG00000052814 | Ankrd27 | 7.47 | 17.82 | down |
| ENSRNOG00000016275 | Ttr | 4757.02 | 11266.72 | down |
| ENSRNOG00000033130 | Mup4 | 73.71 | 174.50 | down |
| ENSRNOG00000002176 | Nectin3 | 7.18 | 17.02 | down |
| ENSRNOG00000007601 | Inhbe | 23.94 | 56.52 | down |
| ENSRNOG00000012749 | C1qb | 18.04 | 42.52 | down |
| ENSRNOG00000004500 | Myc | 7.92 | 18.60 | down |
| ENSRNOG00000010633 | Acsl1 | 237.18 | 554.67 | down |
| ENSRNOG00000009273 | LOC259244 | 2444.20 | 5703.85 | down |
| ENSRNOG00000049811 | LOC684871 | 16.26 | 38.43 | down |
| ENSRNOG00000060310 | LOC688815 | 1.98 | 4.83 | down |
| ENSRNOG00000016924 | Acly | 38.91 | 90.66 | down |
| ENSRNOG00000018086 | Slc22a8 | 68.29 | 158.87 | down |
| ENSRNOG00000029386 | RT1-N2 | 1.77 | 4.17 | down |
| ENSRNOG00000020497 | Plekha1 | 1.45 | 3.42 | down |
| ENSRNOG00000014320 | Inhba | 1.80 | 4.24 | down |
| ENSRNOG00000007990 | Adipor2 | 24.63 | 57.26 | down |
| ENSRNOG00000001926 | Cldn1 | 8.01 | 18.63 | down |
| ENSRNOG00000050595 | Mup5 | 633.02 | 1467.36 | down |
| ENSRNOG00000019716 | Ntf3 | 1.06 | 2.57 | down |
| ENSRNOG00000036984 | Slco1a1 | 38.41 | 88.84 | down |
| ENSRNOG00000006653 | Slc38a4 | 82.52 | 190.75 | down |
| ENSRNOG00000002636 | Abat | 21.97 | 50.82 | down |
| ENSRNOG00000020420 | Pklr | 25.89 | 59.83 | down |
| ENSRNOG00000038607 | Tmem86b | 78.49 | 181.21 | down |
| ENSRNOG00000023587 | Dhtkd1 | 41.53 | 95.82 | down |
| ENSRNOG00000006779 | Crot | 23.84 | 54.98 | down |
| ENSRNOG00000053915 | Setdb2 | 1.17 | 2.87 | down |
| ENSRNOG00000060051 | AABR07049038 | 2.92 | 7.14 | down |
| ENSRNOG00000005931 | Cpq | 63.38 | 144.78 | down |
| ENSRNOG00000025327 | Tert | 1.32 | 3.04 | down |
| ENSRNOG00000050499 | Mup4 | 48.69 | 110.89 | down |
| ENSRNOG00000013397 | Foxo1 | 3.23 | 7.42 | down |
| ENSRNOG00000016516 | Mbp | 1.03 | 2.39 | down |
| ENSRNOG00000002878 | Afm | 282.70 | 641.06 | down |
| ENSRNOG00000029993 | Kynu | 66.07 | 149.67 | down |
| ENSRNOG00000007779 | Kank4 | 0.56 | 1.29 | down |
| ENSRNOG00000025088 | Tmc3 | 1.37 | 3.12 | down |
| ENSRNOG00000011184 | Slc13a4 | 13.11 | 29.45 | down |
| ENSRNOG00000019014 | Ndst1 | 5.95 | 13.37 | down |
| ENSRNOG00000043400 | LOC100910678 | 15.55 | 35.80 | down |
| ENSRNOG00000018322 | Picalm | 10.67 | 23.91 | down |
| ENSRNOG00000008079 | Ugp2 | 52.75 | 117.99 | down |
| ENSRNOG00000031136 | Ntng1 | 0.39 | 0.91 | down |
| ENSRNOG00000009239 | Entpd8 | 2.61 | 5.85 | down |
| ENSRNOG00000007947 | Fam13a | 2.07 | 4.66 | down |
| ENSRNOG00000007139 | Ttpa | 98.00 | 217.69 | down |
| ENSRNOG00000019422 | Egr1 | 41.52 | 92.10 | down |
| ENSRNOG00000056688 | Acnat1 | 24.91 | 55.33 | down |
| ENSRNOG00000011881 | Slc25a15 | 62.74 | 139.09 | down |
| ENSRNOG00000002911 | Alb | 24531.96 | 54360.85 | down |
| ENSRNOG00000033466 | Apon | 77.44 | 171.70 | down |
| ENSRNOG00000007370 | Rnf144a | 0.41 | 0.95 | down |
| ENSRNOG00000054063 | Naa80 | 13.16 | 29.17 | down |
| ENSRNOG00000058186 | Errfi1 | 255.63 | 564.12 | down |
| ENSRNOG00000013589 | Cxcl12 | 23.77 | 52.34 | down |
| ENSRNOG00000033619 | Apof | 150.29 | 330.58 | down |
| ENSRNOG00000023509 | Irs2 | 4.00 | 8.82 | down |
| ENSRNOG00000019318 | Syt3 | 0.48 | 1.11 | down |
| ENSRNOG00000012804 | C1qc | 25.13 | 55.16 | down |
| ENSRNOG00000012404 | Thrsp | 194.16 | 425.13 | down |
| ENSRNOG00000059956 | Bcl6b | 3.62 | 7.97 | down |
| ENSRNOG00000049232 | Tcf7l2 | 2.76 | 6.08 | down |
| ENSRNOG00000007927 | Mettl7b | 142.91 | 312.61 | down |
| ENSRNOG00000009350 | Sez6 | 22.14 | 48.34 | down |
| ENSRNOG00000045553 | Proser2 | 6.16 | 13.52 | down |
| ENSRNOG00000020342 | Samd11 | 3.82 | 8.40 | down |
| ENSRNOG00000016975 | Pxmp4 | 21.79 | 47.49 | down |
| ENSRNOG00000056279 | AC115369 | 0.70 | 1.62 | down |
| ENSRNOG00000048874 | Gckr | 52.56 | 113.83 | down |
| ENSRNOG00000018160 | Zswim5 | 0.32 | 0.74 | down |
| ENSRNOG00000021405 | Cyp2c7 | 324.45 | 702.24 | down |
| ENSRNOG00000019120 | Hmgcs2 | 382.10 | 826.68 | down |
| ENSRNOG00000029988 | AABR07007675 | 4.71 | 10.62 | down |
| ENSRNOG00000011363 | Napepld | 0.97 | 2.19 | down |
| ENSRNOG00000015292 | Cul2 | 4.89 | 10.59 | down |
| ENSRNOG00000021474 | Siglec5 | 0.80 | 1.78 | down |
| ENSRNOG00000021440 | Pptc7 | 3.87 | 8.35 | down |
| ENSRNOG00000059601 | AC127076 | 15.20 | 32.73 | down |
| ENSRNOG00000007387 | Per1 | 5.02 | 10.80 | down |
| ENSRNOG00000025151 | Ildr2 | 0.64 | 1.44 | down |
| ENSRNOG00000018903 | Pik3r1 | 7.04 | 15.12 | down |
| ENSRNOG00000039278 | Mcart1 | 1.87 | 4.04 | down |
| ENSRNOG00000059330 | AABR07004549 | 374.27 | 801.53 | down |
| ENSRNOG00000050949 | Ttc39c | 6.76 | 14.55 | down |
| ENSRNOG00000014297 | Sdc4 | 82.39 | 176.41 | down |
| ENSRNOG00000009470 | Flnb | 2.00 | 4.30 | down |
| ENSRNOG00000047493 | Slco1a4 | 24.74 | 52.96 | down |
| ENSRNOG00000000641 | Nrbf2 | 16.47 | 35.27 | down |
| ENSRNOG00000013291 | Cyp2c23 | 368.10 | 786.19 | down |
| ENSRNOG00000042228 | AABR07061950 | 72.78 | 155.01 | down |
| ENSRNOG00000013704 | Cps1 | 258.54 | 550.13 | down |
| ENSRNOG00000061519 | Asap2 | 1.12 | 2.44 | down |
| ENSRNOG00000054077 | AABR07024870 | 140.51 | 297.51 | down |
| ENSRNOG00000003709 | Kmo | 26.28 | 55.69 | down |
| ENSRNOG00000007529 | Bmf | 6.89 | 14.62 | down |
| ENSRNOG00000022500 | Rwdd4 | 4.94 | 10.51 | down |
| ENSRNOG00000015654 | Ghr | 66.58 | 140.74 | down |
| ENSRNOG00000030776 | Sytl2 | 0.89 | 1.97 | down |
| ENSRNOG00000016349 | Gnmt | 62.92 | 132.95 | down |
| ENSRNOG00000004786 | Cyp4f1 | 195.16 | 409.10 | down |
| ENSRNOG00000019982 | Ethe1 | 26.90 | 56.49 | down |
| ENSRNOG00000019977 | Ptprf | 20.35 | 42.58 | down |
| ENSRNOG00000011367 | Cyp2r1 | 1.52 | 3.27 | down |
| ENSRNOG00000011775 | Mfap3l | 0.82 | 1.78 | down |
| ENSRNOG00000007197 | Nr1h4 | 33.22 | 39.66 | down |
| ENSRNOG00000002906 | Nr1i2 | 42.02 | 55.82 | down |
| ENSRNOG00000002948 | Abcc3 | 0.54 | 0.28 | down |
| ENSRNOG00000010064 | Abcc4 | 1.50 | 1.37 | down |
